# Finite Element Model Updating Combined with Multi-Response Optimization for Hyper-Elastic Materials Characterization

**DOI:** 10.3390/ma12071019

**Published:** 2019-03-27

**Authors:** Saúl Íñiguez-Macedo, Rubén Lostado-Lorza, Rubén Escribano-García, María Ángeles Martínez-Calvo

**Affiliations:** 1 Department of Mechanical Engineering, University of La Rioja, 26004 Logroño, La Rioja, Spain; saul.iniguez@unirioja.es (S.Í.-M.); marian.martinez@unirioja.es (M.Á.M.-C.); 2 IK4-LORTEK, 20240 Ordizia, 20240 Guipuzcoa, Spain; ruben.escribanogarcia@gmail.com

**Keywords:** hyperelastic materials, finite element method, multi-response optimization, model updating

## Abstract

The experimental stress-strain curves from the standardized tests of Tensile, Plane Stress, Compression, Volumetric Compression, and Shear, are normally used to obtain the invariant λi and constants of material C_i_ that will define the behavior elastomers. Obtaining these experimental curves requires the use of expensive and complex experimental equipment. For years, a direct method called model updating, which is based on the combination of parameterized finite element (FE) models and experimental force-displacement curves, which are simpler and more economical than stress-strain curves, has been used to obtain the C_i_ constants. Model updating has the disadvantage of requiring a high computational cost when it is used without the support of any known optimization method or when the number of standardized tests and required C_i_ constants is high. This paper proposes a methodology that combines the model updating method, the mentioned standardized tests and the multi-response surface method (MRS) with desirability functions to automatically determine the most appropriate C_i_ constants for modeling the behavior of a group of elastomers. For each standardized test, quadratic regression models were generated for modeling the error functions (ER), which represent the distance between the force-displacement curves that were obtained experimentally and those that were obtained by means of the parameterized FE models. The process of adjusting each C_i_ constant was carried out with desirability functions, considering the same value of importance for all of the standardized tests. As a practical example, the proposed methodology was validated with the following elastomers: nitrile butadiene rubber (NBR), ethylene-vinyl acetate (EVA), styrene butadiene rubber (SBR) and polyurethane (PUR). Mooney–Rivlin, Ogden, Arruda–Boyce and Gent were considered as the hyper-elastic models for modeling the mechanical behavior of the mentioned elastomers. The validation results, after the C_i_ parameters were adjusted, showed that the Mooney–Rivlin model was the hyper-elastic model that has the least error of all materials studied (MAEnorm = 0.054 for NBR, MAEnorm = 0.127 for NBR, MAEnorm = 0.116 for EVA and MAEnorm = 0.061 for NBR). The small error obtained in the adjustment of the C_i_ constants, as well as the computational cost of new materials, suggests that the methodology that this paper proposes could be a simpler and more economical alternative to use to obtain the optimal C_i_ constants of any type of elastomer than other more sophisticated methods.

## 1. Introduction

An elastomer material is a polymer that has a viscoelastic behavior and very weak intermolecular forces, which cause it to have a much lower Young’s modulus (E) than that of other materials. This reduced E causes this material to possess a great capacity for deformation when proportionally low loads are applied. There are many types of elastomers, but they are classified essentially as unsaturated rubbers that can be cured by sulfur vulcanization, and saturated rubbers that cannot be cured by this process [1]. The strain-stress curves for elastomers have a notable non-linear behavior, which is why Hooke’s law is not applicable. Nevertheless, for small deformations, Young’s modulus (E) can be approximated as the tangent to the strain-stress curve. The values of E that are obtained are of the order of E = 1 N/mm^2^, which is something that makes clear the difference between elastomers and metals, crystals or glasses. Their macroscopic behavior is complex and depends on the time of load application, the temperature, the kind of vulcanizing, the historic loads and the state of deformation. Nevertheless, it is possible in general to achieve large deformations of the order of 100-700%, without entering the plastic region [2]. All those material characteristics make it very difficult to define a model that is able to define their complete behavior. Over the years, several theoretical models have been proposed, and more are being proposed currently. However, there are a few that are used most commonly. These include Mooney–Rivlin [3,4], Arruda–Boyce [5], Gent [6] and Ogden [7]. All of these explain the material behavior macroscopically. The theoretical models are different expressions of the deformation energy functions that are used in the large deformation theory that are determined by a series of invariant λ_i_ values and constants of material C_i_ [8]. That is, due to the complexity of the material, a simple explanation has not been found despite attempts by many authors. No matter what material is selected for a theoretical model, it is necessary to test the elastomer in as many tensional configurations as required. The experimental characterization of these C_i_ constants requires the use of several standardized tests for: Tensile stress [9], compression [10], plane stress, volumetric compression and shear [11]. Currently, there are several methods for measuring the stress-strain field by these tests to obtain the constants of material C_i_. For example, there are those that are known as direct methods [12]: The Moiré method [13,14], electronic speckle pattern interferometry method [15], grid methods [16] and digital image correlation method (DIC) [17,18]. Normally, these methods require expensive and complex experimental equipment to correctly determine the relationship between stresses and strains. Obtaining these elastic constants C_i_ directly from the stress-strain curves is usually achieved by a direct process that is known as curve-fitting. Another classical method that is known as "model updating" is based on inverse problems that are applied to numerical simulations of the finite element method (FEM) [19,20,21]. Model updating bases its operating principle on the hyper-elastic models and their corresponding constants C_i_ that are implemented in the theory of FEM. The implementation of theoretical models with hyper-elastic behavior in the FEM makes it possible to conduct a very precise finite element (FE) analysis in which the same standardized tests that are mentioned above can be modeled. Model updating uses the experimental results (e.g., load-displacement data) for comparison with those load-displacement data that are obtained from the FE simulations. The force-displacement experimental curves that are obtained from standardized tests do not require equipment that is as expensive and complex as that required to obtain the stress-strain experimental curves of these same standardized tests. [16,22,23,24]. If the data that are obtained experimentally and those that have been obtained from the FEM differ significantly, it means that hyper-elastic model that was considered and its corresponding constants C_i_ are inappropriate for simulation of the behavior of the elastomer that is being studied. The theoretical hyper-elastic model and its corresponding constants C_i_ will be varied iteratively. The adjustment will be completed when the difference between the experimentally obtained data and the data obtained from the FEM do not differ significantly. Although this method of adjustment is well-known and used, it usually involves a high computational cost. This problem is amplified when the model of behavior that is being considered is more complex or when the number of standardized tests that are used to adjust the behavior of the studied elastomer is high. For this reason, model updating has never been used individually to adjust the constants C_i_ on the basis of the standardized tests mentioned above. In recent years, researchers have used several techniques to adjust the constants or parameters that define the behavior of FE models on the basis of experimental data. For example, the bio-inspired soft computing techniques and multi-response surface method (MRS) are some of the most frequently used [25,26,27,28]. These techniques usually reduce considerably the number of simulations required for the constants of the FE models that must be adjusted. Other researchers have used the model updating method or a similar procedure to find the parameters that best define the mechanical behavior of a given elastomer. However, in their adjustment, they have used a relatively small number of standardized tests [29,30]. This paper proposes a method to determine automatically the theoretical hyper-elastic material behavior models (Mooney–Rivlin, Ogden, Arruda–Boyce and Gent) and the corresponding C_i_ constants that are most appropriate for modeling the behavior of a group of elastomers. The proposed adjustment method is based on the experimental force-displacement data from the standardized tests, parameterized FE models based on the standardized tests and the multi-response surface method (MRS) with desirability functions. This combination of methods has never been used previously to determine automatically the theoretical hyper-elastic material behavior models and the corresponding C_i_ constants that are most appropriate for modeling the behavior of a group of elastomers using all standardized tests mentioned above. The proposed method was developed as follows: Firstly, several standardized tests (tensile, plane stress, compression, volumetric compression, and shear) were developed in order to obtain the experimental force-displacement curves of each standardized test. Then, each standardized test was replicated with respective FE models so that the Mooney–Rivlin, Ogden, Arruda–Boyce and Gent hyper-elastic models and their corresponding C_i_ constants were parameterized. From these parameterized FE models, the force-displacement curves were obtained. A Box–Behnken design of experiments (DoE) was developed in order to generate, for each of the theoretical hyper-elastic models, a design matrix with which to vary each corresponding C_i_ of the parameterized FE models. Using the force-displacement curves that were obtained from the FE models, and the MRS, quadratic regression models were generated for each standardized test for modeling an error function (ER). The latter will be minimized by applying it to each of the standardized tests. The EF represented the distance between the force-displacement curves that were obtained experimentally and those that were obtained by means of the parameterized FE models. This enabled us to mathematically model the distance between both force-displacement curves according to the C_i_ proposed in the design matrix. The best hyper-elastic materials models and their corresponding C_i_ constants were found when the ER functions defined for each of the standardized tests were minimized. This adjustment process was undertaken using desirability functions. As a practical example, the proposed methodology is validated with nitrile butadiene rubber (NBR), ethylene-vinyl acetate (EVA), styrene butadiene rubber (SBR) and polyurethane (PUR) elastomers. Agreement between the force-displacement curves that were obtained by the most appropriate FE models and those that were obtained experimentally demonstrates that the proposed methodology may be valid for determining automatically the most appropriate hyper-elastic model and its corresponding constants C_i_ to use to correctly define the hyper-elastic behavior of several elastomers. Also, the method that is proposed can be applied quickly for the optimal search of the C_i_ constants for a variety of elastomers. That is, only the experimental phase to obtain the force-displacement curve and the quadratic regression that is used to obtain the ER and its minimization by means of desirability functions would be the new phases that are required to adjust the constants C_i_ of the new elastomer. This means that the method that this paper proposes could be a faster, simpler and more economical way to obtain the C_i_ constants of any type of elastomer than other more sophisticated methods.

## 2. Testing Process

In order to conduct the standardized tests, it was necessary to prepare the test samples according to the standards established for the use of these tests. In this section, the standards, regulations, procedures, and methods that were followed to conduct the tests are provided. The samples were manufactured at the normalized temperature according to ISO 471:1995 [31] for at least three hours before being cut and remained at that temperature while they were stored. Also, for each of the materials studied in this work, three samples corresponding to each of the standardized tests were tested in order to obtain three different force-displacement curves. The force-displacement curves obtained from each test were averaged so that a single force-displacement curve was obtained for each material that was studied and for each standardized test. This procedure was followed to reduce uncertainty in the testing process. Tensile tests were performed according to standard ISO 37:2017 [9]. A type 2 test specimen was used, instead of type 3 and type 4, due to the experience of researchers with these types of specimens in previous studies. Also, the direction of the laminate was considered for each specimen. In this case, it was decided to use two specimens in the laminate direction and two in the perpendicular direction and, then, to average the force-displacement curves obtained from the four specimens tested. The dimensions of the specimens were: 20 mm long for the straight section, 4 mm wide and 2 mm thick (See Figure S1).There are no formal procedures for the plane stress test. However, the commonly accepted testing procedure can be established on the basis of experience and various authors [16,22,24]. In this case, the test specimen was a band of 290 × 35 × 2 mm The compression test was developed according to standard ISO 7743:2017 [10]. In this case, the specimen consists of a cylinder that is 29 ± 0.5 mm in diameter and 12.5 ± 0.5 mm in height that is located between two parallel metal plates. As in the case of the plane stress test, there is no associated standard procedure, and the most commonly used method must be established according to the bibliography [22]. The specimens were cylinders that were 20 mm in diameter and 29 mm in height, they were confined in a chamber. The test began with a preload of 50 N to fix the assembly. It continued with material stabilization by 20 cycles of between 0 and 0.5 mm of displacement. When the specimen was preloaded, the testing machine began to compress at a constant speed of 5 mm/min until a force of 9 kN was applied. To apply the pressure, a cylindrical pusher of 20 mm diameter was fixed to a clamp that permitted attachment to the testing machine by a pin system. Finally, the shear test was developed and regulated by the standard ISO 1827:2007 [11]. The specimens were composed of four identical rubber parallel parts with dimensions according to the standard of 4 ± 1 mm × 20 ± 5 mm × 25 ± 5 mm. The specimens were glued to four steel plates, which served to transmit the shear stress to the specimens, as well as holding them in the testing machine. The steel plates were manufactured according to the standard guidelines as follows: two lateral plates of 65 mm × 20 mm and two central plates of 25 mm × 20 mm with a hole of Ø 12 mm to anchor the assembly to the testing machine by means of a pin of Ø 12 mm. The configuration of each of these standardized tests can be found in the Additional Material that is attached to this work (See Section S1).

## 3. Finite Element Models Proposed

**Figure 1:**
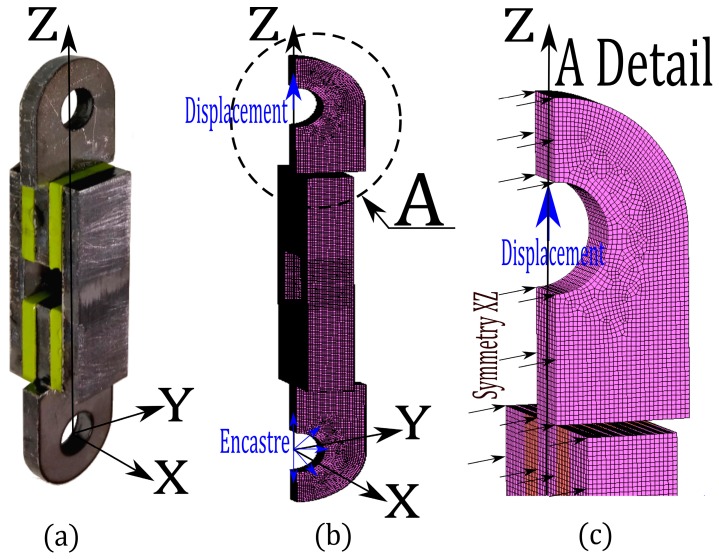
(**a**) Specimen mounted on the steel sheets designed for the shear test. (**b**) Proposed parameterized symmetric finite element (FE) model. (**c**) Details of the proposed FE model in which the nodes of the upper hole in which displacement was imposed are visible.

All the standardized tests were replicated in detail using parameterized three-dimensional FE models with the MSC Mentat-Marc software (MSC, Santa Ana, CA, USA) [32]. The parametrization of the FE models considered the hyper-elastic models (Mooney–Rivlin, Ogden, Arruda–Boyce and Gent), as well as their corresponding C_i_ for the elastomers that were studied. For steel parts, an isotropic model was considered with an elastic modulus and Poisson ratio (E = 210000 MPa and μ = 0.3) respectively. Although some of the proposed FE models could be simplified by flat elastic conditions in order to reduce the simulation time because of its large size (i.e., plane stress test), all parameterized FE models in this paper were configured as three-dimensional FE models. The parameterized FE model that was proposed for the tensile test was based on the type 2 test specimen according to ISO 37:2017 [9]. In this case, hexahedral elements with linear formulation were selected for modeling the specimen. Also, full integration with Herrmann formulation was undertaken. In order to guarantee the stability of the FE model and facilitate its convergence, the movement of the central nodes in the direction perpendicular to the axis of the testing machine was restricted (Z axle). This restriction of the displacement of the central nodes of the FE model represented a symmetry condition in the simulation. In the lower part of the FE model, a group of nodes had their entire displacement restricted in order to create the conditions of the pneumatic fixing system of the jaw. In the upper part of the FE model, a displacement ramp that will deform a specimen to a maximum value of 400 mm was applied to a group of nodes in order to cause the displacement of the mobile jaw. Also, due to the size of the required dimensions of the specimen together with the steel plates for the planar stress test, and to reduce the computational cost of the numerical simulations, a parameterized symmetric FE model was proposed for this test. This FE model considers the steel plates but does not consider the screws and nuts, since they are not relevant for the FE simulation. The entire set was built in a single block, thereby eliminating the need to impose contact conditions between the steel plates and the elastomers to be tested. The symmetry condition in the FE model was modeled by imposing null displacement in a direction that is perpendicular to the axis of the testing machine (Z axle) for the central nodes. This condition of symmetry (in the Y axle), while it reduced the computational cost of the FE model, also reduced the instabilities of the FE model, facilitating its convergence. Also, the nodes that correspond to the lower hole were fixed and a displacement ramp was applied on those nodes that correspond to the upper hole. This displaced the FE model to a maximum value of 50 mm. In practice, the cylindrical elastomer material was glued to a 50 × 50 mm steel sheets for the compression test. For the parameterized FE model, a square steel plate was considered to be a circular sheet with a diameter of 50 mm. This simplification was carried out on the parameterized FE model, mainly because the areas near the corners of the steel square did not influence the results that were obtained from the force-displacement curves. Also, a quarter of the FE model was created, and symmetry conditions for the XZ and YZ planes were imposed, whereas the nodes were clamped in the lower plane of the FE model. In the upper plane, movement was permitted in the direction of compression and a maximum displacement of -3.9 mm was undertaken. The volumetric compression test is similar to the compression test. In this case, the cylindrical elastomer was deformed inside a cylindrical chamber. Therefore, its displacement in the radial direction, as well as in its base (Z direction), was restricted. In order to reduce computational cost and improve the convergence of the proposed FE model, a quarter of the specimen with symmetry conditions in the XZ and YZ planes was proposed to model the volumetric compression test. Also, displacement in the radial direction was restricted for the periphery nodes, whereas displacement of the lower nodes was fixed with a null value and the upper nodes were displaced by a maximum value of -0.6 mm. Finally, the proposed parameterized FE model for the shear test is similar to the one that was proposed for planar stress due to the similarity in the arrangement of the elastomer parallelepipeds to the steel parts (Figure 1a). In this case, the four steel plates and the four elastomer parallelepipeds are modeled by symmetry (XZ) along the longitudinal direction of the assembly to be tested (Figure 1b). The boundary conditions, in this case, consisted of the imposition of zero displacement in the direction perpendicular to the plane of symmetry XZ. The nodes of the lower hole were fixed and a displacement ramp was added to those nodes that correspond to the upper hole that deformed the FE model by a maximum value of 10 mm (Figure 1c). The configuration of the remaining parameterized FE models that are proposed according to the other standardized tests can be found in the Additional Material that is attached to this work (See Section S2).

### Mesh Sensitivity Analysis

**Figure 2:**
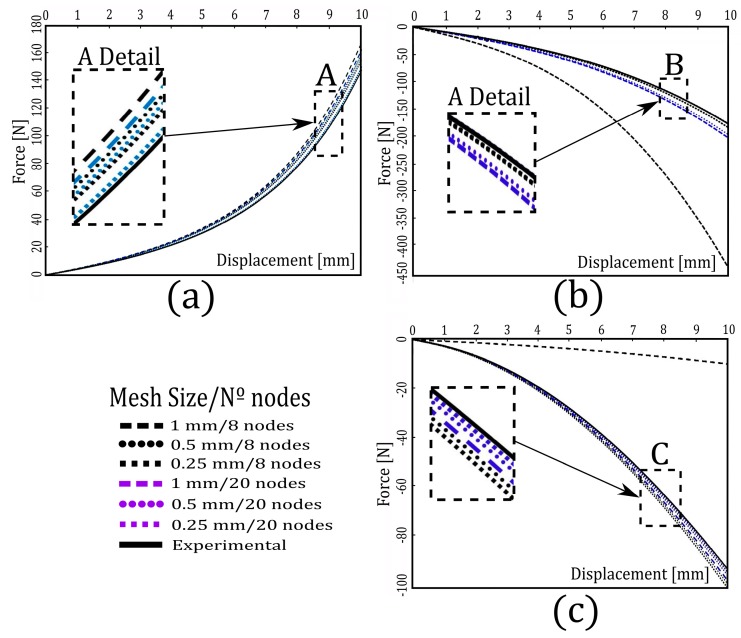
Comparison of the force-displacement curves that were obtained from the FE models and experimentally at different element sizes and formulations (linear/quadratic) for: (**a**) the compression test, (**b**) the shear test, and (**c**) the tensile test.

After each of the parameterized FE models was generated, an analysis of sensibility for all standardized tests was conducted. The reason for this analysis was to determine the correct size of the elements, as well as the type of element and its numerical formulation used to ensure good results at the lowest computational cost. For all parameterized FE models, element sizes of 0.25, 0.5 and 1 2 mm and linear and quadratic formulation (8 and 20 nodes), respectively, were used. The force-displacement values that were obtained from the parameterized FE models for each of the standardized tests were compared to known experimental values. In this case, the experimental values corresponded to an elastomer that shared C_i_ constants with the Mooney–Rivlin model (C_10_ = 0.030747, C_01_ = 0.042667 and C_11_ = 0.027110). The correct size of the elements, as well as the type of element and its numerical formulation, correspond to those parameterized FE models whose force-displacement curves obtained do not differ significantly from those curves that were obtained experimentally at the lowest computational cost. Figure 2 shows, for each of the standardized tests, the force-displacement curves that were obtained from the parameterized FE models with element sizes of 0.25, 0.5 and 1 mm and linear and quadratic formulation (8 and 20 nodes), respectively, and the curves that were obtained experimentally. As a general rule, these figures show that, as the size of the element decreases and its number of nodes increases (20 nodes and quadratic formulation), the difference between the force-displacement curves that were obtained from the FE models and those that were obtained experimentally is smaller. For example, Figure 2a shows the force-displacement curves from the FE model for the compression test and the one obtained experimentally when the displacement was the maximum (10 mm). In this case, the forces obtained from the FE models for this displacement of 10 mm was 167.05, 159.41, and 154.58 N. for linear formulation with element sizes of 0.25, 0.5 and 1 mm, respectively, and was 161.96, 157.10 and 149.25 N., respectively, for the quadratic formulation with the same element size. For the same displacement, the force that was obtained experimentally was 147.01 N. Figure 2b shows the force-displacement curves for the shear test. In this case, the force obtained from the FE models was -441.28, -184.33 and -184.24 N. for linear formulation with element sizes of 0.25, 0.5 and 1 mm respectively, whereas the force was -203.39, -197.28 and -177.36 N. for the quadratic formulation with the same element sizes. The force value that corresponds to a displacement of 10 mm, in this case, was -177.94 N. Finally, Figure 2c shows the force-displacement curves for the tensile test. In this case, the force that was obtained from the FE models was -9.92, -101.31 and -99.68 N. for linear formulation with element sizes of 0.25, 0.5 and 1 mm respectively, but was -98.27, -96.19and -94.84 N. for the quadratic formulation with the same element sizes. The force value that corresponds to a displacement of 10 mm, in this case, was -93.70 N.

Table 1 was created from these curves and those that correspond to the planar stress and volumetric compression tests, as well as the proposed parameterized FE models. This table shows for each standardized test, mesh size (1 mm, 0.5 mm, 0.25 mm) and formulation studied (linear/quadratic), the number of elements and nodes of the proposed FE model, the computational cost and the absolute mean error (MAE). The MAE was calculated for each of the standardized tests from the force-displacement curves that were obtained from the FE simulations and those force-displacement curves that were obtained experimentally. The MAE was defined according to Equation (1).

(1) $$MAE=\frac{1}{m}\overset{m}{\underset{k=1}{\sum }}\left|Y_{k\;EXP}-Y_{k\;FEM}\right|$$

where *Y_EXP_* are the forces that were obtained experimentally for a value of displacements *k*, and *Y_FEM_* are those forces that were obtained from the FE simulations for the corresponding values of displacement *k* and m is the number of force-displacement values that were used to make the adjustment. Table 1 shows that, as the size of the elements decreased and when the proposed FE models had a quadratic formulation (20 nodes), the computational cost increased for all standardized tests that were conducted. The table also shows that, as the size of the elements decreased, the MAE also decreased for both FE models with a linear and quadratic formulation. The smallest MAEs obtained were for mesh sizes of 0.25 mm and quadratic formulation.

To characterize the hyperelastic materials, as proposed in this paper by the use of FE models according to standardized tests, a mesh size of 0.5 mm and a linear formulation were selected. This decision was made because the values of MAE that were obtained, corresponding to a mesh size of 0.5 mm with linear formulation, are reduced (5.94 for tensile test, 8.62 for planar stress, 8.45 for compression, 3.36 for volumetric compression and 5.17 for shear test), whereas their computational costs are acceptable (6 min for the tensile test, 143 min for the planar stress, 201 min for compression, 52 min for volumetric compression and 400 min for the shear test). All FE models proposed were simulated in eight computers with Intel Xeon processor, CPU 2.2GHz (two processors) and 128.00 GB (random access memory (RAM)).

## 4. Modeling with the RSM

The multi-response surface (MRS) is used to optimize objective functions. It is used occasionally to find the minimum or maximum cost, or simply to learn which combination of input variables provide the most suitable output. Box and Wilson introduced this method in 1951 [33] to process experimentally-generated data and create optimum response models. The chemical industry has used MRS methodology extensively because its design facilitates the modeling of experimental responses. It has been used recently, along with other techniques, to optimize industrial processes and products. In effect, MRS is a combination of statistical techniques based on low-degree polynomial regression models.

(2) $$Y=f\left(x_{1},x_{2},x_{3}\ldots x_{k}\right)+e$$

where *Y* is the response function of the *X_n_* factors and an error coefficient *e*. The functions that are used most frequently are linear or quadratic. Cross products among the variables being studied provide the basis of the model. The quadratic model appears below.

(3) $$Y=b_{0}+\overset{n}{\underset{i=1}{\sum }}b_{i}X_{i}+\overset{n}{\underset{i=1}{\sum }}b_{ii}X_{i}^{2}+\overset{n-1}{\underset{i=1}{\sum }}\overset{n}{\underset{j=i+1}{\sum }}b_{ij}X_{i}X_{j}+e$$

The first linear term above is followed by a quadratic one. Then come the cross products of the different variables. However, these functions do not always give satisfactory results for complex problems that have many nonlinearities and inputs. The reason is that, as continuous functions, they are defined by polynomials and, when there are insufficient data, cannot be adjusted. The polynomic coefficients are adjusted by regression analysis. After this adjustment, the p-value (or Prob. > F) indicator is used to select the terms. The indicator determines the probability of obtaining a result that equals or exceeds what has been observed. The indicator can be acquired by using variance analysis (ANOVA). Models from that selection can be simplified by deleting terms that have no effect on the predictions. If the model’s Prob. > F and no term exceeds the level of significance (e.g., α = 0.05), one can consider the model to be acceptable within a (1-α) confidence interval. A problem that has more than one output is termed a multi-response problem. In such cases, the responses normally conflict with each other. Thus, the responses may have differing optimums. In cases of multiple responses, it is desirable to create a regression model for each variable. This provides several processes to determine the range where values are optimum. A widely used method, which Harrington presented, involves desirability functions to find a compromise among the outputs. The procedure requires defining a desirability function for each output and, also, an overall desirability function that is related to the geometric mean of the variable’s desirability functions.

To maximize, they use the following:

(4) $$d_{r}^{máx}=\{\begin{array}{c} \;0\;If\;f_{r}\left(X\right)<L\;\\ {\left(\frac{f_{r}\left(X\right)-L}{T-L}\right)}^{S}\;If\;L\leq f_{r}\left(X\right)\leq T\;\\ 1\;If\;f_{r}\left(X\right)>T \end{array}$$

While minimizing, the function looks like:

(5) $$d_{r}^{mín}=\{\begin{array}{c} \;1\;If\;f_{r}\left(X\right)<T\;\\ {\left(\frac{U-f_{r}\left(X\right)}{U-T}\right)}^{S}\;If\;T\leq f_{r}\left(X\right)\leq U\;\\ 0\;If\;f_{r}\left(X\right)>U \end{array}$$

For a concrete value, use the following:

(6) $$d_{r}^{obj}=\{\begin{array}{c} 0\;If\;f_{r}\left(X\right)<L\\ {\left(\frac{f_{r}\left(X\right)-L}{T-L}\right)}^{S}\;If\;L\leq f_{r}\left(X\right)\leq T\\ {\left(\frac{U-f_{r}\left(X\right)}{U-T}\right)}^{S}\;If\;T\leq f_{r}\left(X\right)\leq L\\ 0\;If\;f_{r}\left(X\right)>U \end{array}$$

The overall desirability function is created by multiplying all other functions to the power of the inverse of *R*, where *R* is the number of functions.

(7) $$D={\left(\overset{R}{\underset{r=1}{\prod }}d_{r}\right)}^{1/R}$$

where *L* is the lower value and *U* is the upper value, *T* is the target value and *S* is the exponent that determines how import of attaining the desired value. *X* is the input vector. The applied functions are only models used for the prediction. Applying the desirability functions requires that the responses be transformed into non-dimensional values of between 0 and 1. The closer to one that the value is, the greater the response is. In the case of a two input and one output, one can represent a three-dimensional space. Doing so helps to determine the location of the maximums and minimums for locating the extreme values. However, it is important to know the approximate area in which the extreme values occur. After the area has been defined, a design of experiments (DoE) is necessary in order to obtain results from this variable range. The number of variables in the problem that is being studied will vary depending on the error function that is selected and the degree of adjustment necessary. The polynomial models will predict the error that will arise between the test data. It also will predict the force of the input values of the energy model constants and the displacement experienced during the test. There must be a polynomial model for each test for use in the objective function to minimize the total error.

## 5. Combining FEM and MRS to Optimize Mechanical Problems

FE model updating has the disadvantage of involving a high computational cost, especially when the process of fitting the FE models and the constants that best define their behavior are based only on FE simulations and trial and error. The adjustment becomes more complicated when the FE model is used to solve non-linear problems, such as hyper-elastic material behavior, plasticity, and mechanical contacts. However, the computational cost can be reduced by using RSM since regression models are generated with the objective of approximating the problem that is modeled by models based on the FEM. These regression models are able to learn from the most representative constants of the FEM models, as well as their simulation results. The ultimate goal of these RSM-based regression models is simply to reduce the number of simulations that are necessary to fit the FE models and the constants that best define their behavior. In this regard, the combination of RSM and FEM has been used widely to optimize many industrial processes [34], to optimize mechanical elements [35] and to adjust the constants that best define the mechanical behavior of biomechanical FE models [27]. To date, no author has used FE model updating combined with multi-response optimization for hyper-elastic materials characterization. In this paper, the method that is proposed is based on the experimental force-displacement data that have been obtained from the standardized tests, parameterized FE models based on the standardized tests and multi-response surface method (MRS). It was developed as follows: Firstly, several standardized tests (tensile, plane stress, compression, volumetric compression, and shear) were developed in order to obtain the experimental force-displacement curves of each test. Subsequently, each of the standardized tests was replicated with parameterized FE models including the Mooney–Rivlin, Ogden, Arruda–Boyce and Gent hyper-elastic models and their corresponding C_i_. The force-displacement curves of these parameterized FE models were obtained. A Box-Behnken Design of Experiments (DoE) was developed in order to generate, for each of the proposed hyper-elastic models, a design matrix with which to vary each of the corresponding C_i_ of the parameterized FE models. The main objective of the DoE is to cover the entire range of possibilities in the most normal way possible and with the least possible data. The best hyper-elastic materials models and their corresponding constants C_i_ were adjusted when the difference between the force-displacement curves that were obtained experimentally and those that were obtained by the FE simulations on the basis of standardized tests did not differ significantly. This adjustment process was performed using desirability functions, and previously required the definition of the error function (*ER*), which will be applied to each of the standardized tests to be minimized. The *EF* represented the distance between the force-displacement curves obtained experimentally from those force-displacement curves that were obtained by means of the parameterized FE models. It was defined as follows:

(8) $$EF_{j}=\overset{m}{\underset{k=1}{\sum }}\left|Y_{jk\;EXP}-Y_{jk\;FEM}\right|$$

In this case, *Y_jk EXP_* are the forces that were obtained experimentally for a value of displacements *k* according to the standardized *j*, and *Y_FEM_* are those forces that were obtained from the FE simulations for the corresponding values of displacement *k* according to the same standardized *j,* whereas *m* is the number of force-displacement values that were used to make the adjustment. Then, using MRS, quadratic regression models were generated for each of the standardized tests in order to model mathematically all *EF_j_*. After each of the *EF_j_* was calculated for each of the standardized tests, it was normalized to adjust the hyper-elastic models and their corresponding C_i_ using desirability functions. The data is usually normalized in statistical processes to ensure that all variables are changed to the same scale (i.e., from 0 to 1). Normalization enables a comparison of mathematical models of outputs with different units. In this case, the normalization consists of subtracting the minimum value from each original value of *EF_j_* and then dividing the result by the range (See Equation (9)).

(9) $$EF_{j\;norm}=\frac{EF_{j}-\min\left(EF_{j}\right)}{\max\left(EF_{j}\right)-\min\left(EF_{j}\right)}$$

where *EF_j norm_* are the normalized errors functions, *EF_j_* are the errors functions that were obtained from the regression models that were developed with RSM and range *(EF)* is the range where the *EF_j_* are defined. The subscript *j* represents each of the standardized tests (tensile, plane stress, compression, volumetric compression, and shear). After the *EF_j norm_* was obtained, the adjustment process was undertaken using desirability functions.

## 6. Case Study

This section presents a case study for modeling hyper-elastic materials behavior models (Mooney–Rivlin, Ogden, Arruda–Boyce and Gent) and their corresponding C_i_ constants that are most appropriate for modeling the behavior of a group of elastomers. As a practical example, the proposed methodology is validated with nitrile butadiene rubber (NBR), ethylene-vinyl acetate (EVA), styrene butadiene rubber (SBR) and polyurethane (PUR) elastomers.

### 6.1. Experimental Results

**Figure 3:**
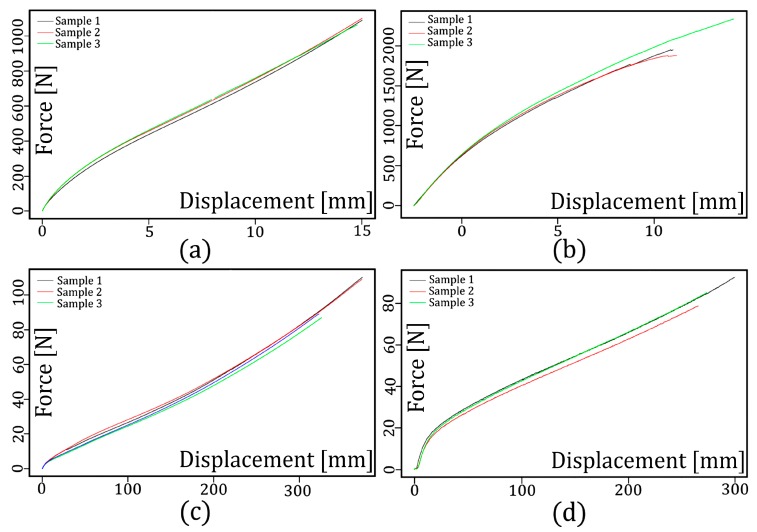
(**a**) Shear test for the nitrile butadiene rubber (NBR) material, (**b**) shear test for the polyurethane (PUR) material, (**c**) tensile test for the NBR material, and (**d**) tensile test for the PUR material.

According to Section 2, each of the studied materials was tested by the standardized tests to obtain the experimental force-displacement data. Figure 3 shows the force-displacement curves of the shear test and the tensile test for some of the elastomers to which the proposed methodology was applied. As shown in this figure, each of these studied materials was tested three times in order to reduce the uncertainty in the testing process. The remaining curves for each of the materials studied, as well as those corresponding to each of the standardized tests, can be found in the Additional Material that is attached to this work (See Section S3). Figure 3a corresponds to the force-displacement curves of the shear test for the NBR material, whereas Figure 3b corresponds to that of the PU material. Figure 3c corresponds to the force-displacement curves of the planar stress test for the NBR material, whereas Figure 3d corresponds to that of the PUR material. A total of twenty force-displacement curves were obtained to apply the proposed methodology for hyper-elastic material characterization. Also, the figures indicate that the behavior of the materials in the different tests differ entirely. This suggests that each of them can be characterized by a different hyper-elastic material behavior model.

### 6.2. Design of Experiments

RSM must create a design of experiments (DoE) [36] to obtain accurate models with the least data for support of the initial hypotheses. DoE has been developed by the use of several methods. However, all require that a design matrix (input constants) be constructed to measure the experimental outputs or responses. In this case, because the proposed hyper-elastic models present a different number of input constants (eight constants for the Mooney–Rivlin, seven constants for the Arruda–Boyce, seven constants for the Gent and seven constants for the Ogden), a combination of 3k and 5k full-factorial design of experiments with three factors and three levels were used to develop the experiment [37]. Table 2 shows the input constants, the notation and the limits of each input constant.

Using the statistical open source software R (r-project) [38], 125 for Mooney–Rivlin, 9 for Arruda–Boyce, 25 for Gent and 25 for Ogden parameterized FE models with their corresponding input constants C_i_ were generated for each of the standardized tests according to the input constants C_i_ and levels that appear in Table 2. After each of the FE models was simulated, a total of 21 results with their corresponding constants C_i_ and their outputs (forces) were obtained for each of the standardized tests, as well as for each of the proposed hyper-elastic models. Thus, for example, the 5k Mooney–Rivlin model had a total of 125 × 21 = 2,625 results and their corresponding constants C_i_ and outputs (forces) (See Table 3). The remaining tables that relate to the materials that were studied can be found in the Additional Material that is attached to this work (See Section S4).

After all, force-displacement curves were obtained from the FE simulations, and with the homologous force-displacement curves that were obtained experimentally, the values of EF_norm_ were calculated for each standardized test according to Equation (9).

### 6.3. Modeling the EF for the Materials Studied According to the Standardized Tests

Considering all *EFs* calculated as output and each constant C_i_ that defined the four proposed hyper-elastic material behaviors as input, a polynomial fitting regression process was developed using the RMS “R” package [38]. The objective of this process was to obtain, for each of the four hyper-elastic material behavior proposals and each of the four materials studied, a second-order polynomial model to model the *EF* according to each of the standardized tests. Equation (10) shows the second-order polynomial model that was obtained for the *EF* corresponding to the Mooney–Rivlin hyper-elastic model for the NBR material in the shear test. The remaining equations that correspond to the materials that were studied, as well as those that correspond to the standardized tests can be found in Section S5 of the Additional Material that is attached to this work.

(10) $$\begin{array}{l} EF_{shear}=183.52116-693.53046\cdot C_{10}+1400.42875\cdot C_{10}{}^{2}-278.43835\cdot C_{10}{}^{4}-\\ 640.16174\cdot C_{01}+3456.7162\cdot C_{10}\cdot C_{01}-2482.67744\cdot C_{10}{}^{2}\cdot C_{01}+609.80965\cdot C_{10}{}^{3}\cdot C_{01}+\\ 1443.64739\cdot C_{01}{}^{2}-2414.77072\cdot C_{10}\cdot C_{01}{}^{2}+802.19476\cdot C_{10}{}^{2}\cdot C_{01}{}^{2}+570.90191\cdot C_{10}\cdot \\ C_{01}{}^{3}-429.79401\cdot C_{01}{}^{4}+2053.13932\cdot C_{10}\cdot C_{11}-882.06251\cdot C_{10}{}^{2}\cdot C_{11}+1163.36567\cdot \\ C_{01}\cdot C_{11}-1288.34095\cdot C_{10}\cdot C_{01}\cdot C_{11}+911.5076\cdot C_{11}{}^{2}-3050.22947\cdot C_{10}\cdot C_{11}{}^{2}+\\ 1662.59232\cdot C_{10}{}^{2}\cdot C_{11}{}^{2}-1734.73345\cdot C_{01}\cdot C_{11}{}^{2}+1520.55779\cdot C_{10}\cdot C_{01}\cdot C_{11}{}^{2}+\\ 559.5721\cdot C_{01}{}^{2}\cdot C_{11}{}^{2} \end{array}$$

In order to determine the generalization capacity of the second-order polynomial regression models that was obtained for modeling the *ER*, the mean absolute error (MAE) and a root mean square error (RMSE) were defined according to Equations (11) and (12).

(11) $$MAE=\frac{1}{m}\sum_{k=1}^{m}\left|EF_{\left(FEM\right)K}-EF_{\left(R\_MODELS\right)K}\right|$$

(12) $$RMSE=\sqrt{\frac{1}{m}\sum_{k=1}^{m}{\left(EF_{\left(FEM\right)K}-EF_{\left(R\_MODELS\right)K}\right)}^{2}}$$

In this case, *EF_(FEM)K_* and *EF_(R_MODELS)K_* were, respectively, each of the error functions (*ER*) that were obtained from the parameterized FE models and from the second-order polynomial regression models when considering each of the constants C_i_ (i.e., from 1 to m) that appear in Table 2. These MAE and RMSE that were calculated directly from the constants C_i_ are shown in Table 3. They were used to generate the regression models and are known as training errors (train.MAE and train.RMSE). In addition, new samples or parameterized FE models that are based on each standardized test that is defined with different constants C_i_ for the hyper-elastic material behavior and each of the four materials that were studied were used to test the regression models obtained and, thus, to obtain the testing errors of said regression models (test.MAE and test.RMSE). Ten parameterized FE models with their corresponding constants C_i_ for Mooney–Rivlin, five for Arruda–Boyce, five for Gent and five for Ogden were generated respectively for each standardized test. The new constants C_i_ for use in defining the parameterized FE models were generated randomly. They were not used previously to generate regression models and differ from those constants C_i_ that appear in Table 2. After these new parameterized FE models were simulated, their force-displacement curves were obtained, and the values of the *ER_(FEM)k_* were recalculated according to Equation (8) for each standardized test. For each of the four materials studied, the new samples with their corresponding constants C_i_ were used to calculate the *ER_(R_MODELS)K_* values with each of the second-order polynomial model Equations. Finally, with the values of *ER_(FEM)k_* and *ER_(R_MODELS)K_* the tests’ errors were calculated (test.MAE and test.RMSE) using Equations (11) and (12). The correlation (Corr), the *p*-value and the MAE and the RMSE for both the training and testing phases were calculated for each polynomial model obtained. All of the polynomial models that were analyzed provided correlation values (Corr) close to “1” whereas most *p*-values were less than 0.01. This indicates that the inputs (or the constants C_i_ that define the hyper-elastic behavior of the elastomers that were studied) that composed the second-order polynomial models are statistically significant. However, most values of MAE and RMSE that were obtained in the training phase (train.MAE and train.RMSE) and testing phase (test.MAE and test.RMSE) are less than 10%. This indicates that the second-order polynomial models have a good predictive capacity. In addition, these results indicate that the Mooney–Rivlin is the hyper-elastic material behavior of minor test errors, whereas the Arruda–Boyce is the material behavior of a major error for each standardized test and each of the four elastomers that were studied. These results of correlation (Corr), *p*-value, MAE and RMSE for each of the polynomial models can be found in Section S6 of the Additional Material that is attached to this work.

### 6.4. Multi-Response Optimization

The search for the combination of C_i_ constants that are most appropriate for modeling the mechanical behavior of NBR, EVA, SBR and PUR materials according to hyper-elastic models (Mooney–Rivlin, Arruda–Boyce, Gent and Ogden) was developed with the use of the RMS “R” package [39] by desirability functions. In this case, the EF_norm_ that was calculated by Equation (9), and was used, instead of EF because it was intended that all standardized tests be of equal importance in the adjustment of the C_i_ constants. Section S7 of the Additional Material that is attached to this work (Tables S25–S28), provides the results of the adjustment of the constants C_i_ of the hyper-elastic models of the studied material. For the NBR material (Table S25), the values of the overall desirability obtained for each of the hyper-elastic models were: 0.951 for the Mooney–Rivlin, 0.753 for the Arruda–Boyce, 0.777 for the Gent model, and 0.759 for the Ogden model. In addition, this table shows that some of the values are very close to the targets that were proposed. For example, the minimum target proposed for the EF_Norm,Shear_ was 96.174 and the optimal value that was obtained was 96.07 with a desirability value of one for the Mooney–Rivlin model. In contrast, for the Arruda–Boyce model, the proposed target for the EF_Norm,Tens_ was 115.47 and the optimal value that was obtained was 292.62 with a desirability value of 0.482. For the PUR material (Table S26), the values of the overall desirability that was obtained in this case for each hyper-elastic model were: 0.821 for the Mooney–Rivlin, 0.532 for the Arruda–Boyce, 0.645 for the Gent and 0.671 for the Ogden. Like the results in Table S25, the results in Table S26 show that some of the values that were obtained are also close to the proposed targets. For example, the minimum target proposed for the EF_Norm,Shear_ was 112.854 and the optimal value that was obtained was 111.625 with a desirability value of one. This was also true for the Mooney–Rivlin model. In contrast, for the Gent, the proposed target for the EF_Norm,ComVol_ was 249.261 and the optimal value that was obtained was 6276.983 with a desirability value of 0.246. For the EVA material (Table S27), the overall desirability values that were obtained in this case for the Hyper-elastic models were: 0.952 for the Mooney–Rivlin, 0.674 for the Arruda–Boyce, 0.644 for the Gent and 0.682 for the Ogden. This table also shows that the minimum target proposed for the EF_Norm,Comp_ was 78.213 and that the optimal value that was obtained was 113.191 with a desirability value of 0.981. These values were the same for the Mooney–Rivlin model. In contrast, for the Gent, the proposed target for the EF_Norm,Comp_ was 13.739 and the optimal value was 331.24 with a desirability value of 0.472. Finally, for the SBR material (Table S28), the values of the overall desirability, in this case, were: 0.949 for the Mooney–Rivlin, 0.747 for the Arruda–Boyce, 0.741 for the Gent and 0.765 for the Ogden. This table also shows that the minimum target proposed for the EF_Norm,Shear_ was 91.287 and that the optimal value that was obtained was 162.006 with a desirability value of 0.959 also for the Mooney–Rivlin. In contrast, for the Arruda–Boyce, the proposed target for the EF_Norm,ComVol_ was 63.944 and the optimal value that was obtained was 2677.111 with a desirability value of 0.501. As a result of the optimization process, it can be concluded that the Mooney–Rivlin is the hyper-elastic model that provides the best fit between the force-displacement curves obtained experimentally and by FEM for the four materials studied because its overall desirability is the highest. Gent and Arruda–Boyce are the hyper-elastic models that provided the worst adjustment between the force-displacement curves because they have the lowest overall desirability. In addition, the shear is the standardized test that achieves the best fit between the force-displacement curves for the NBR and PUR materials, because it has desirability of one. Also, for the EVA and SBR materials, the standard compression and tensile tests best adjust the force-displacement curves, as evidenced by desirability of 0.981 and 0.993, respectively. Table 4 indicates the most appropriate or optimal constants C_i_ to correctly define the hyper-elastic behavior that were adjusted by MRS with desirability functions.

**Figure 4:**
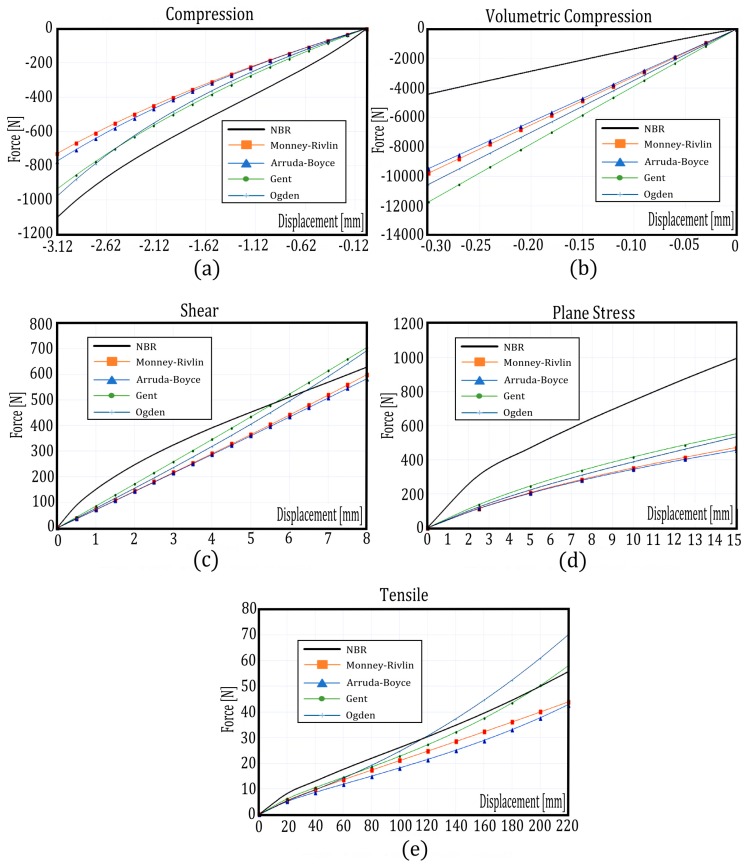
Force-displacement curve obtained from the FE simulations when the optimal constants C_i_ are compared to the force-displacement obtained experimentally for the hyper-elastic Mooney–Rivlin model for NBR material in standardized tests (**a**) compression, (**b**) volumetric compression, (**c**) shear, (**d**) plane stress, and (**e**) tensile.

After the optimal constants C_i_ were obtained, they were validated by comparing the force-displacement curves that were obtained experimentally to the curves that were obtained by the parameterized FE models when constants C_i_ are considered. Figure 4 shows the force-displacement curves for the NBR material and for each of the standardized tests when the optimal constants C_i_ considered are those of Table 4. This figure shows that the distance between the force-displacement curve obtained experimentally and those obtained from the parameterized FE models considering the optimal constants C_i_ is greater for the volumetric compression and planar stress tests (see Figure 4b,d), in which, its desirability values obtained for the hyper-elastic models are for Mooney–Rivlin 0.932 and 0.9, respectively, for Arruda–Boyce 0.513 and 0.482, for Gent 0.573 and 0.497, and finally for Ogden 0.643 and 0.485. For other materials, the adjustment between the force-displacement curves that were obtained experimentally and those that were obtained from the parameterized models is very similar to the NBR material, which can be found in Section S8 of the Additional Material that is attached to this work. Thus for example, for the PUR material (See Figure S14), EVA material (See Figure S15) and SBR material (See Figure S16), the distances between the force-displacement curves obtained experimentally and those obtained from the parameterized FE models is also greater for the volumetric compression and plane stress tests. Table 5 shows the average MAE_norm_ that have been obtained for each of the materials that were studied and for each one of the hyper-elastic models, which were calculated from the force-displacement curves that were obtained experimentally and those that were obtained from the parameterized FE models by use of the optimal constants C_i_ that appear in Table 4. Each of the MAE_norm_ in this table has been calculated as the sum of the MAE_norm_ obtained for each of the standardized tests according to Equations (34) and (51). For all the materials studied, it was observed that the hyper-elastic model that has the least error in adjusting the force-displacement curves is the Mooney–Rivlin model (MAE_norm_ = 0.054 for NBR, MAE_norm_ = 0.127 for NBR, MAE_norm_ = 0.116 for EVA, and MAE_norm_ = 0.061 for NBR). However, this table also shows the total computational cost to obtain the optimal C_i_ constants for each of the materials studied (i.e., the FE simulations, the quadratic regression necessary to obtain the ER functions and its minimization by means of desirability functions). As mentioned previously, only the experimental phase to obtain the force-displacement curve, and the quadratic regression necessary to obtain the ER and its minimization by means of desirability functions, would be the new phases required to adjust the constants C_i_ of any elastomer that differs from those discussed in this paper. This means that the method that is proposed in this paper could be a faster, simpler and more economical way to obtain the C_i_ constants of any type of elastomer than other more sophisticated methods.

## 7. Conclusions

The experimental characterization of the C_i_ constants for correct modeling of the hyper-elastic behavior of elastomers requires the use of several standardized tests (tensile, plane stress, compression, volumetric compression, and shear). These tests require expensive and complex experimental equipment to correctly determine the relationship between stresses and strains. For years, a direct method called model updating, which is based on the combination of parameterized finite element (FE) models and experimental force-displacement curves, which are simpler and more economical than stress-strain curves, has been used to obtain the C_i_ constants. This method has the disadvantage of a high computational cost when it is used without the support of a known optimization method or when the number of standardized tests and required C_i_ constants is high. This paper proposes a methodology that combines the model updating method, the aforementioned standardized tests and the multi-response surface method (MRS) with desirability functions to determine automatically the most appropriate C_i_ constants for modeling the behavior of a group of elastomers. The optimization ascribed the same value of importance to all the standardized tests. An error function (EF) is defined to determine the distance between the force-displacement curves obtained experimentally, and their homolog force-displacement curves obtained from the parameterized FE models that are based on the standardized tests mentioned. Second-order polynomial regression models are generated from these EFs and used to search for the C_i_ constants. As a practical example, the proposed methodology was validated with the following elastomers: nitrile butadiene rubber (NBR), ethylene-vinyl acetate (EVA), styrene butadiene rubber (SBR), and polyurethane (PUR). Mooney–Rivlin, Ogden, Arruda–Boyce and Gent were considered to be the hyper-elastic models for modeling the mechanical behavior of the elastomers. After the optimal C_i_ constants were found by use of the polynomial regression models by MRS with desirability functions, the validation results determined that the Mooney–Rivlin model was the hyper-elastic model with the least error for all materials that were studied (MAE_norm_ = 0.054 for NBR, MAE_norm_ = 0.127 for NBR, MAE_norm_ = 0.116 for EVA and MAE_norm_ = 0.061 for NBR). The optimal C_i_ constants obtained with the Mooney–Rivlin for each of the studied material were: C_10_ = 0.367 MPa, C_01_ = -0.069 MPa, C_11_ = 0.005 MPa for NBR material, C_10_ = 0.982 MPa, C_01_-0.056 MPa, C_11_ = 0.005MPa for PUR material, C_10_ = 0.572 MPa, C_01_ = -0.292MPa, C_11_ = 0.002 MPa for EVA material, and C_10_ = 0.112 MPa, C_01_ = 0.152 MPa, C_11_ = 0.005 MPa for SBR material. From these results, it is observed that the optimal values obtained for C_11_ are in a very small range (0.002 to 0.005). The proposed method can be applied quickly to the optimal search of the C_i_ constants for a variety of elastomers. That is, the experimental phase to obtain the force-displacement curve, as well as the quadratic regression models that are necessary to obtain the ER and its minimization by desirability functions, would be the new phases that are required to adjust the constants C_i_ of the new elastomer. The small error obtained in the adjustment of the C_i_ constants and the computational cost for new elastomers suggest that the proposed methodology in this paper could be a simpler and more economical way to obtain the optimal C_i_ constants of any type of elastomer than more sophisticated methods.

## Figures and Tables

**Table 1:** Element size, number of elements, number of nodes, computational cost and absolute mean error (MAE) corresponding to each of the standardized tests for FE models of a quadratic and linear formulation.

Test	Size [mm]	Nº Elements	N° Nodes	Time [min]	MAE
			(Linear/ Quadratic)	(Linear/ Quadratic)	(Linear/ Quadratic)
Tensile	1	1568	1950/5850	1/5	65.05/3.54
	0.5	11440	7190/21570	**6**/180	**5.94**/1.96
	0.25	91520	28762/86290	76/1728	4.71/0.90
Planar Stress	1	10150	10872/32612	16/106	12.66/8.57
	0.5	81200	43482/130442	**143**/1232	**8.62**/2.08
	0.25	649600	173922/521762	1980/12000	3.35/1.81
Compression	1	4866	2223/6668	13/493	11.95/9.61
	0.5	35904	8480/25438	**201**/1952	**8.45**/6.49
	0.25	281424	33544/100628	1598/9000	6.04/1.44
Volumetric Compression	1	2958	1371/3746	6/28	1968.48/35.14
	0.5	22156	5361/15241	**52**/474	**3.36**/8.97
	0.25	165416	19441/58320	740/14000	2.98/2.55
Shear test	1	20560	8264/24792	36/280	174.20/20.33
	0.5	163928	59496/179328	**400**/4833	**5.17**/15.47
	0.25	1308160	329702/992708	10163/	5.01/0.38

**Table 2:** Independent variables and experimental design levels used with the 3k and 5k full-factorial design for the proposed hyper-elastic models.

Hyper-Elastic Model	DoE	Input Constant	Magnitude	Levels				
				−1	−0.5	0	0.5	1
Mooney–Rivlin	5k	C_10_	MPa	−0.25	0.13	0.5	0.88	1.25
		C_01_	MPa	−0.3	0.03	0.35	0.68	1
		C_11_	MPa	0	0.13	0.25	0.38	0.5
Arruda–Boyce	3k	Nkt	-	0.26	--	0.58	--	0.9
		Chain	-	2	--	13.5	--	25
Gent	5k	E	MPa	0.6	1.375	2.15	2.925	3.7
		inv		63	67.125	71.25	75.375	79.5
Ogden	5k	K1	-	0	0.125	0.25	0.375	0.5
		K2	-	−0.5	−0.3125	−0.125	0.062	0.25

**Table 3:** Design matrix and experiments obtained with a 5k DoE for the hyper-elastic Mooney–Rivlin model of the NBR material used in the shear test.

Inputs					Output
Sample	C_10_	C_01_	C_11_	Displacement	Force
	(MPa)	(MPa)	(MPa)	(mm)	(N)
1	−0.25	0.35	0.125	0.00	0.000
2	−0.25	0.35	0.125	0.50	12.117
3	−0.25	0.35	0.125	1.00	24.695
4	−0.25	0.35	0.125	1.50	38.228
5	−0.25	0.35	0.125	2.00	53.264
6	−0.25	0.35	0.125	2.50	70.419
7	−0.25	0.35	0.125	3.00	90.374
8	−0.25	0.35	0.125	3.50	113.829
9	−0.25	0.35	0.125	4.00	141.470
10	−0.25	0.35	0.125	4.50	173.949
11	−0.25	0.35	0.125	5.00	211.880
…	…	…	…	…	…
2623	1.25	1.00	0.500	9.00	7288.559
2624	1.25	1.00	0.500	9.50	7975.014
2625	1.25	1.00	0.500	10.00	8703.273

**Table 4:** Adjustment of the C_i_ constants of the hyper-elastic models for the materials studied.

Hyper-Elastic Models	C_i_	NBR	PUR	EVA	SBR
**Mooney–Rivlin**	C_10_ (MPa)	0.367	0.982	0.572	0.112
	C_01_ (MPa)	−0.069	−0.056	−0.292	0.152
	C_11_ (MPa)	0.005	0.005	0.002	0.005
**Arruda–Boyce**	Nkt	0.578	0.643	0.567	0.579
	Chain	24.644	3.75	15.054	17.354
**Gent**	E (MPa)	2.144	2.982	2.237	1.899
	inv1	76.465	70.645	63.1	79.46
**Ogden**	k1	0.254	0.329	0.361	0.124
	k2	−0.261	−0.499	−0.119	−0.426

**Table 5:** Average MAE_norm_ obtained for each of the materials and each one of the hyper-elastic models studied.

Hyper-Elastic Models	NBR		PUR		EVA		SBR	
	MAE_norm_	Time (min.)	MAE_norm_	Time (min.)	MAE_norm_	Time (min.)	MAE_norm_	Time (min.)
Mooney–Rivlin	0.054	802	0.127	840	0.116	720	0.061	870
Arruda–Boyce	0.194	668	0.536	715	0.246	621	0.225	742
Gent	0.282	725	0.916	767	0.426	708	0.361	737
Ogden	0.054	905	0.736	963	0.381	893	0.287	926

## Supplementary Materials

The following are available online at https://www.mdpi.com/1996-1944/12/7/1019/s1.

## List of Symbols/Nomenclature

**Symbol**	**Nomenclature**
λi	Invariant
ANOVA	Analysis of the variance
C_10_, C_01_, C_11_	Mooney–Rivlin material constants
Chain	Arruda–Boyce material constant max length of a chain
C_i_	Constants of material
DIC	Digital image correlation method
DoE	Design of experiments
E	Young’s Modulus; Gent material constant
ER	Error function
EVA	Ethylene-vinyl acetate
FE	Finite element
FEM	Finite element method
Inv	Gent material constant, invariant
K1, K2	Ogden material constants
MAE	Mean absolute error
MRS	Multi-response surface method
Nkt	Arruda–Boyce material constant, Number of chains in a material network
NBR	Nitrile butadiene rubber
PUR	Polyurethane
RAM	Random access memory
RMSE	Root mean square error
SBR	Styrene butadiene rubber
