# Supplementary material for: Finite Element Model Updating Combined with Multi-Response Optimization for Hyper-Elastic Materials Characterization
Source: Materials (Basel). 2019 Mar 27;12(7):1019. doi: 10.3390/ma12071019 (PMC6479898; doi:10.3390/ma12071019)
Supplement: Supplementary file 1 [file materials-12-01019-s001.pdf]

materials

Additional Material

Finite Element model updating combined with Multi-Response Optimization for Hyperelastic Materials Characterization

Saúl Íñiguez-Macedo [1], Rubén Lostado-Lorza [1,*] Rubén Escribano-García [2] and María Ángeles Martínez-Calvo [1].

[1] Department of Mechanical Engineering, University of La Rioja, 26004 Logroño, La Rioja, Spain; saul.iniguez@unirioja.es (S.I.M)
[2] IK4-LORTEK, 20240 Ordizia, Guipuzcoa, Spain; ruben.escribanogarcia@gmail.com
* Correspondence: ruben.lostado@unirioja.es; Tel.: +34-941-299-527

Received: date; Accepted: date; Published: date

S1. Standardized tests

S1.1. Tensile test

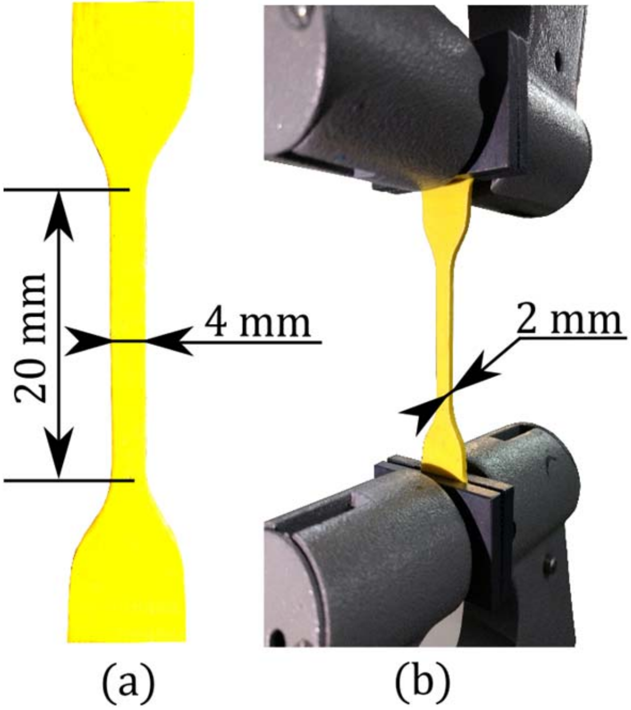

Figure S1. (**a**) Specimens ready for testing. (**b**) Specimen subjected to the tensile test.

S1.2. Plane Stress test

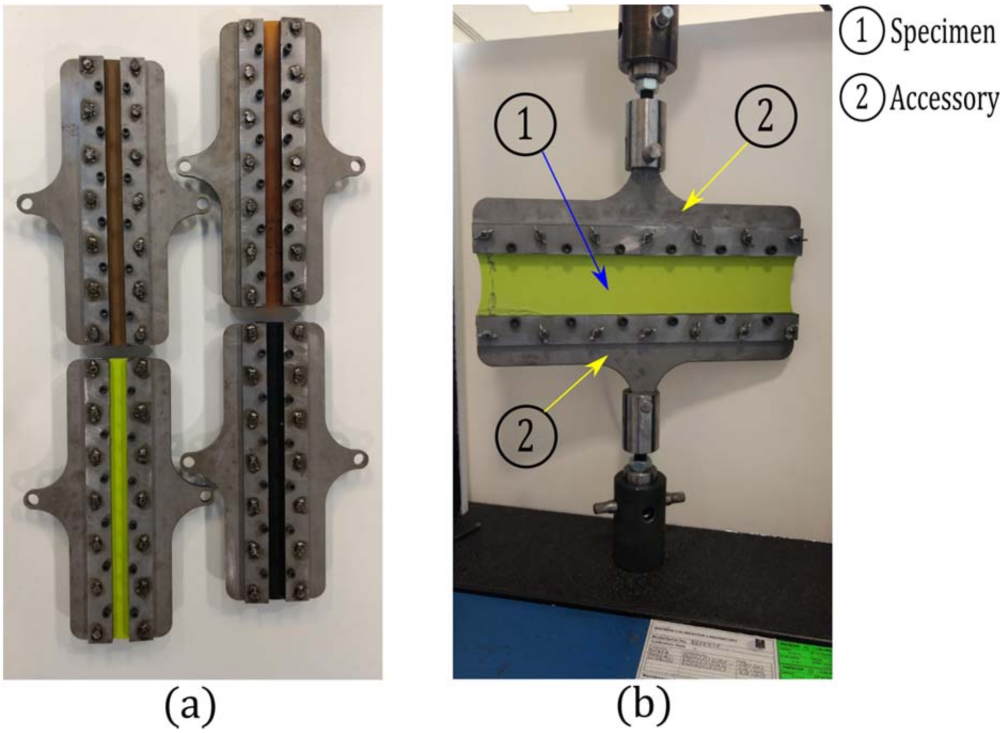

Figure S2. (**a**) Specimens mounted on the accessory designed for the plane stress test. (**b**) Development of this test in the tensile testing machine.

S1.3. Compression test

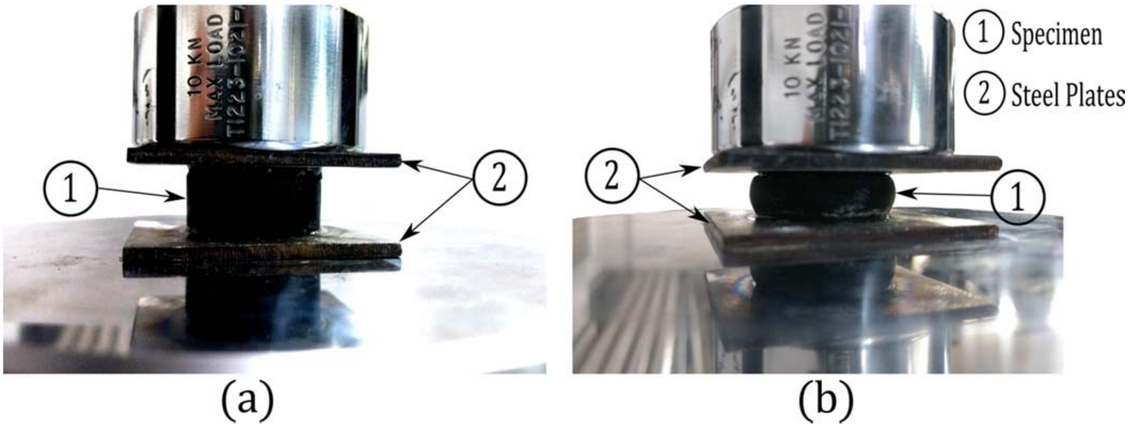

Figure S3. (**a**) Specimen glued to two parallel planes according to Method B. (**b**) Development of the compression test according to Method B in the tensile testing machine.

S1.4. Volumetric compression test

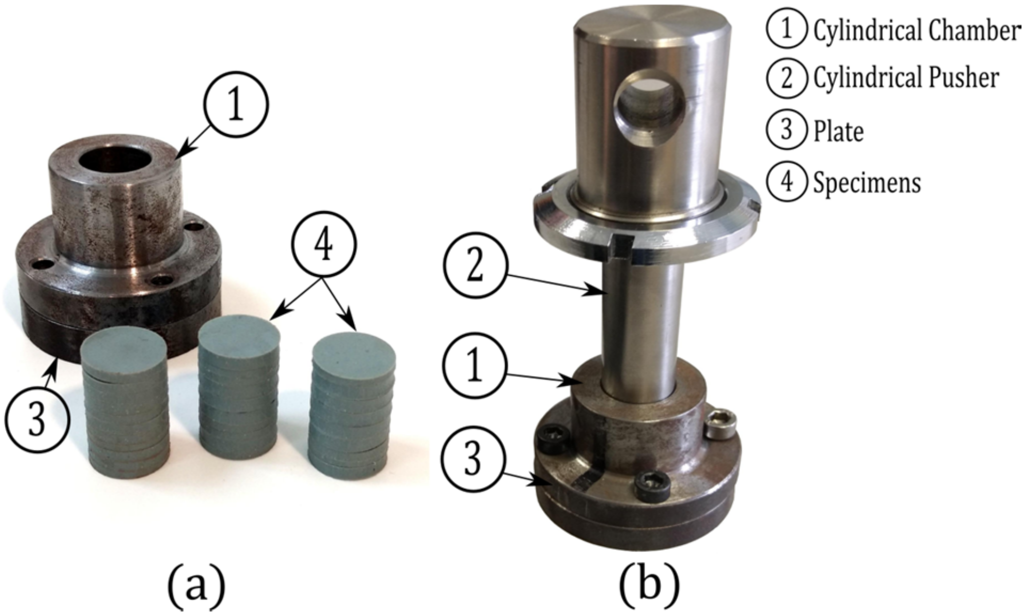

Figure S4. (**a**) Specimens ready for testing in the volumetric compression test and the cylindrical chamber with the plate. (**b**) Cylindrical chamber, the plate, the cylindrical pusher and the clamp.

S1.5. Shear test

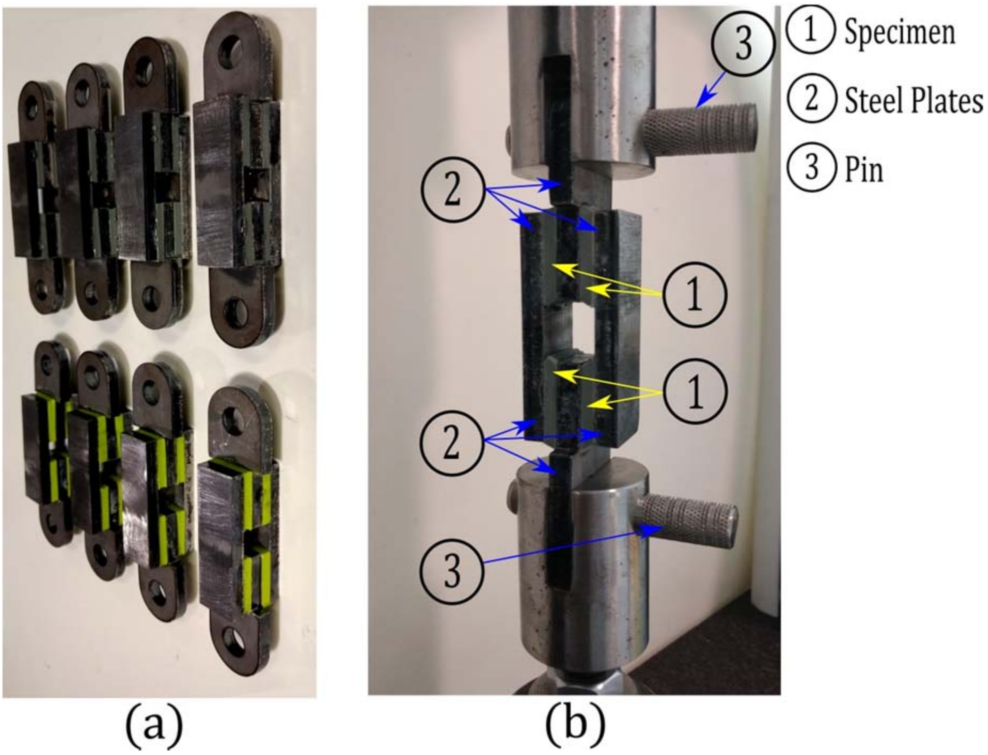

Figure S5. (**a**) Specimens ready for testing in the Shear test. Detail of the four steel plates and the four rubber parallelepipeds mounted on the testing machine.

S2. Finite Element Models Proposed

S2.1. Tensile test parameterized FE model

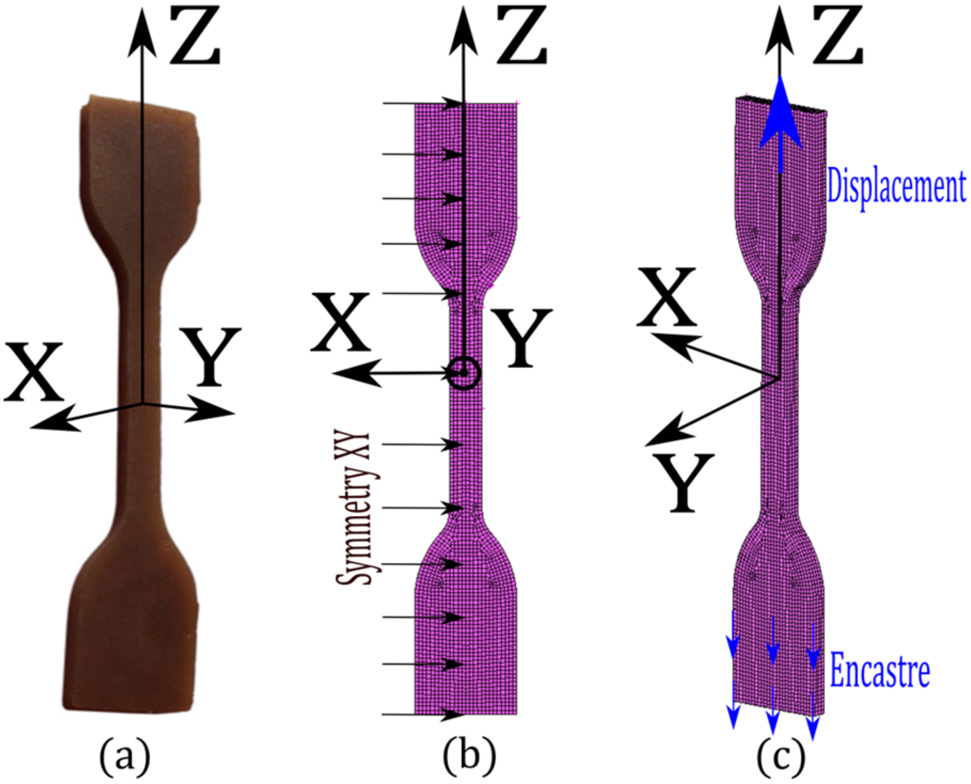

Figure S6. (**a**) Type 2 test specimen according to ISO 37:2017 [9]. (**b**) 3D FE model proposed with the symmetry condition imposed to facilitate its convergence. (**c**) Boundary conditions corresponding to the pneumatic fixing system of the jaws.

S2.2. Planar stress test parameterized FE model

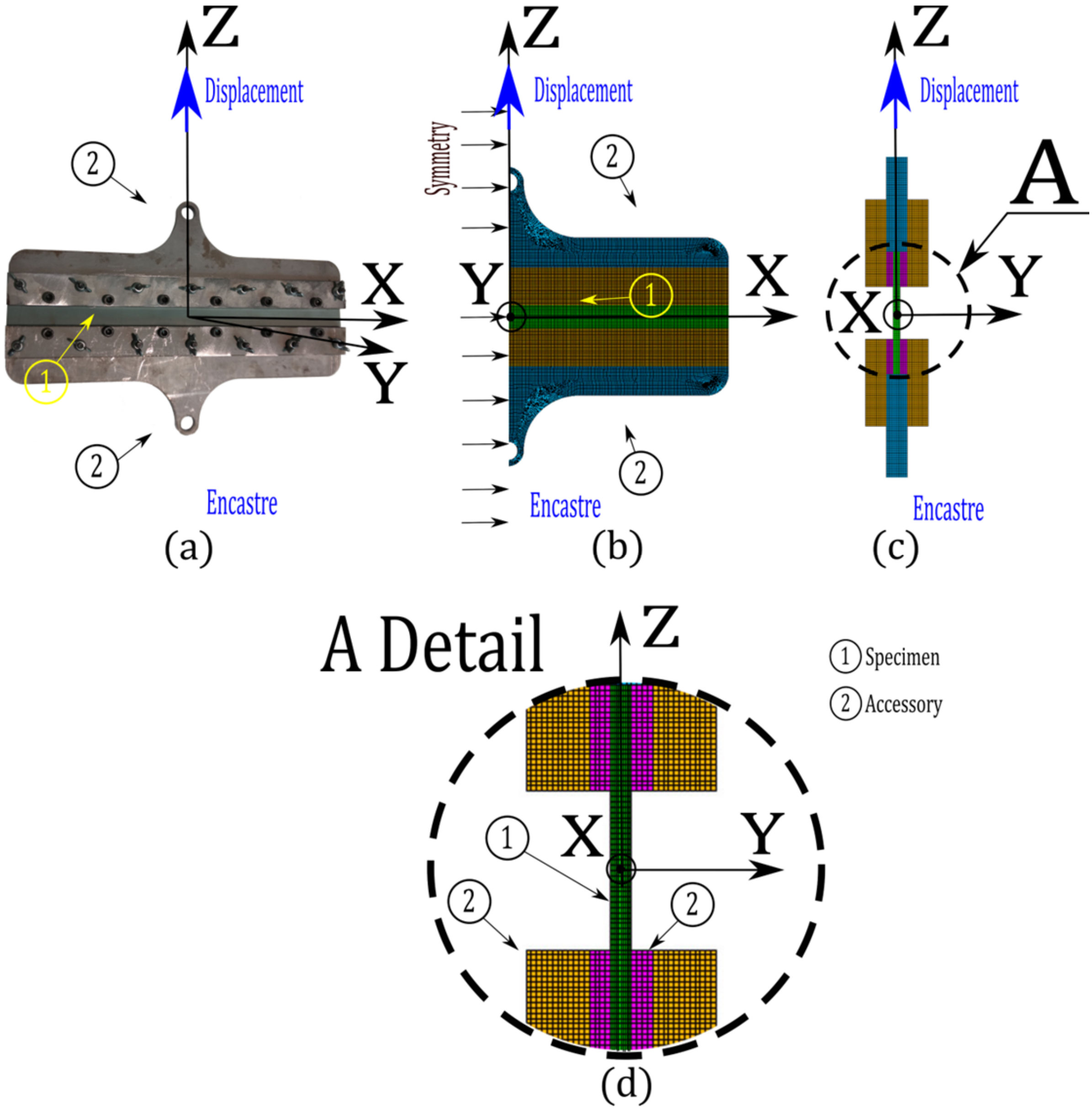

Figure S7. (**a**) Specimen mounted on the accessory designed to conduct the plane stress test. (**b**) Proposed parameterized symmetric FE model. (**c**) Displacement and symmetry conditions applied to the proposed FE model. (**d**) Detail of the specimen holder within the designed accessory.

S2.3. Compression test of the parameterized FE model

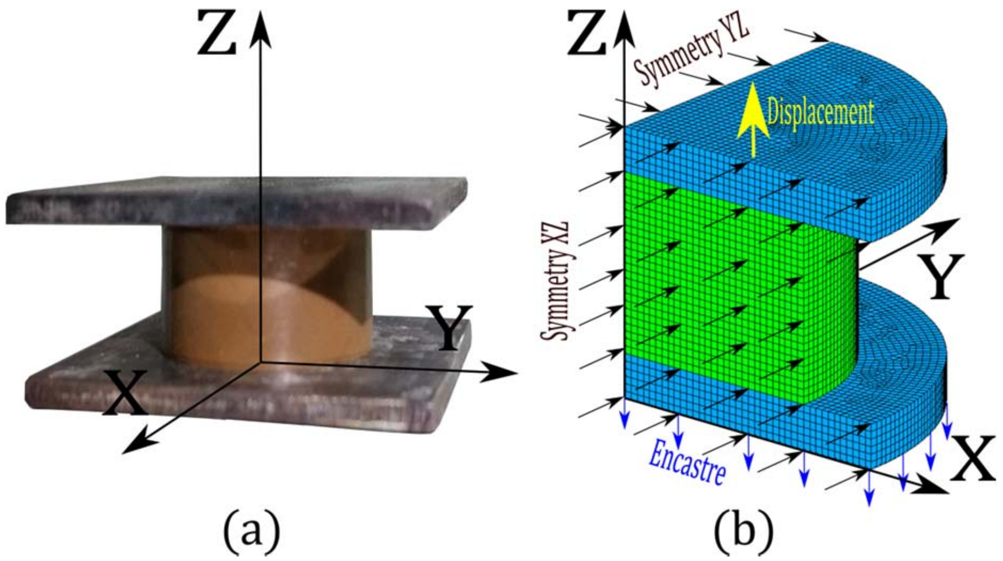

Figure S8. (**a**) Specimen mounted on the steel sheets designed for the compression test. (**b**) A quarter of the FE Parameterized symmetric proposed FE model.

S2.4. Volumetric compression test parameterized FE model

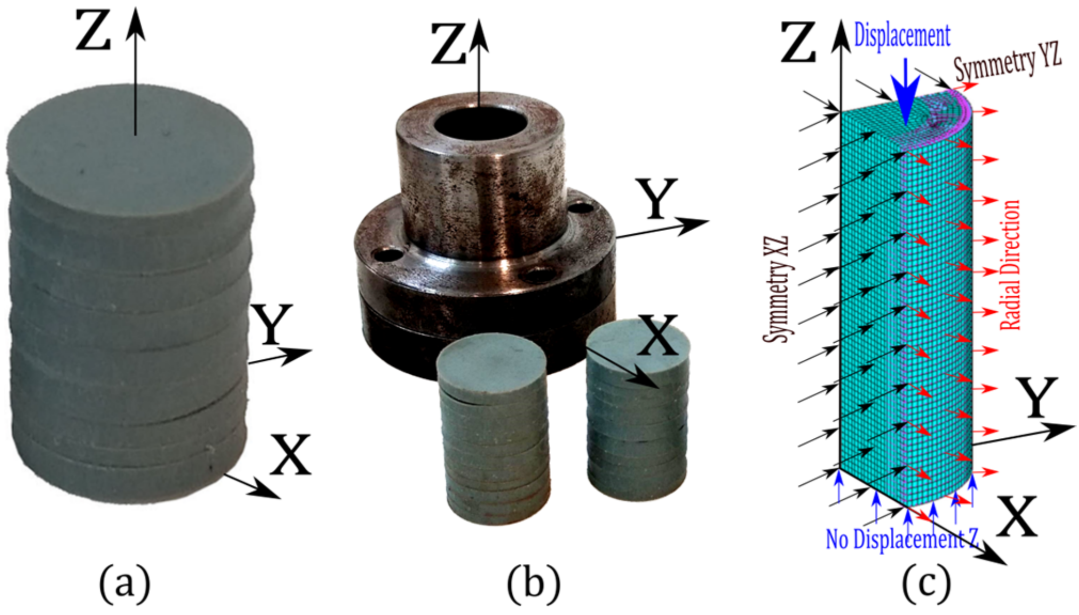

Figure S9. (**a**) Specimen prepared for mounting on the cylindrical chamber. (**b**) Cylindrical chamber and specimens for testing. (**c**) The proposed parameterized symmetric FE model.

S3. Experimental Results

S3.1. NBR Material

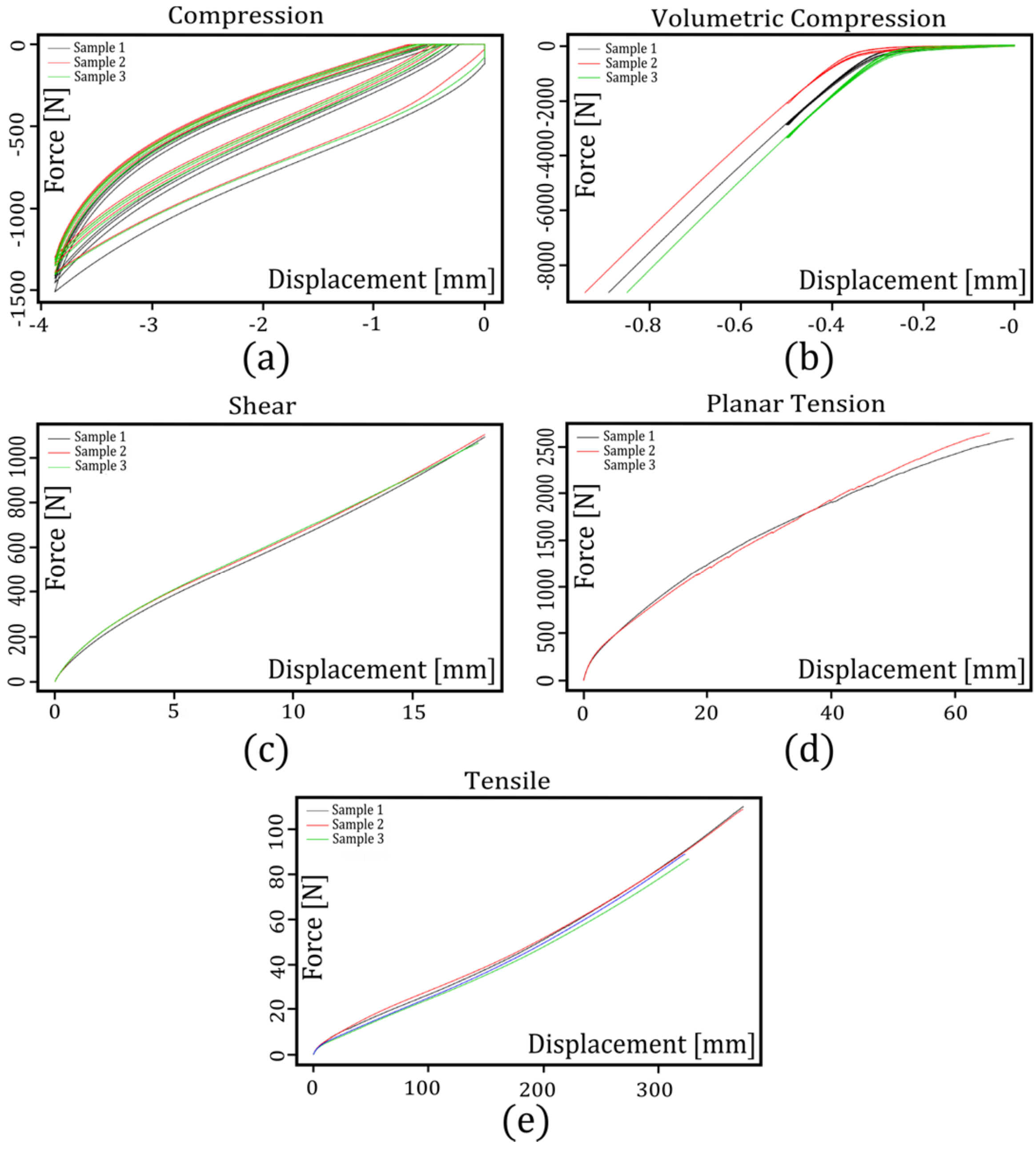

Figure S10. (**a**) Compression test for the NBR material; (**b**) Volumetric Compression test for the NBR material; (**c**) Shear test for the NBR material; (**d**) Planar Tension test for the NBR material, and (**e**) Tensile test for the NBR material.

S3.2. PUR Material

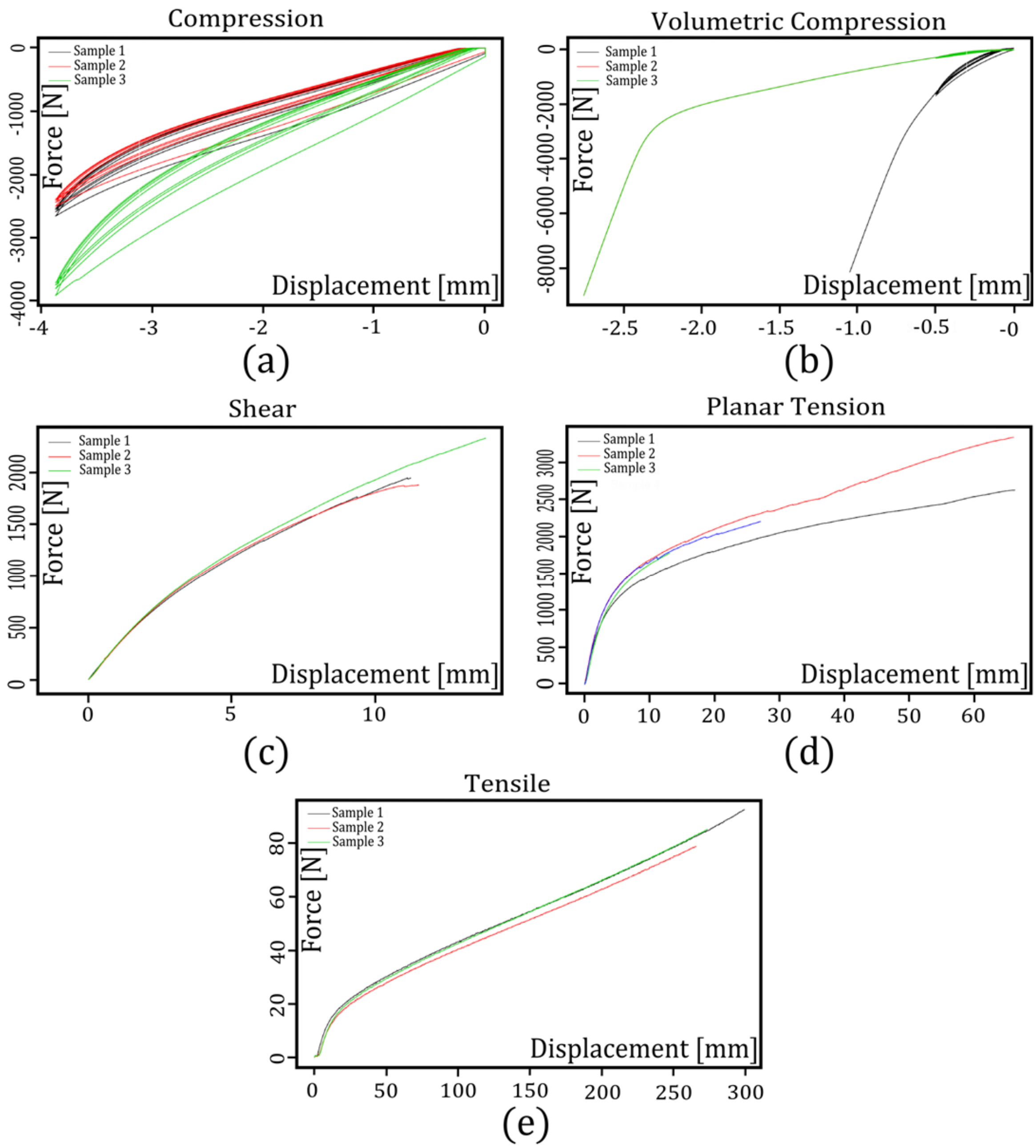

Figure S11. (**a**) Compression test for the PUR material; (**b**) Volumetric Compression test for the PUR material; (**c**) Shear test for the PUR material; (**d**) Planar Tension test for the PUR material, and (**e**) Tensile test for the PUR material.

S3.3. EVA Material

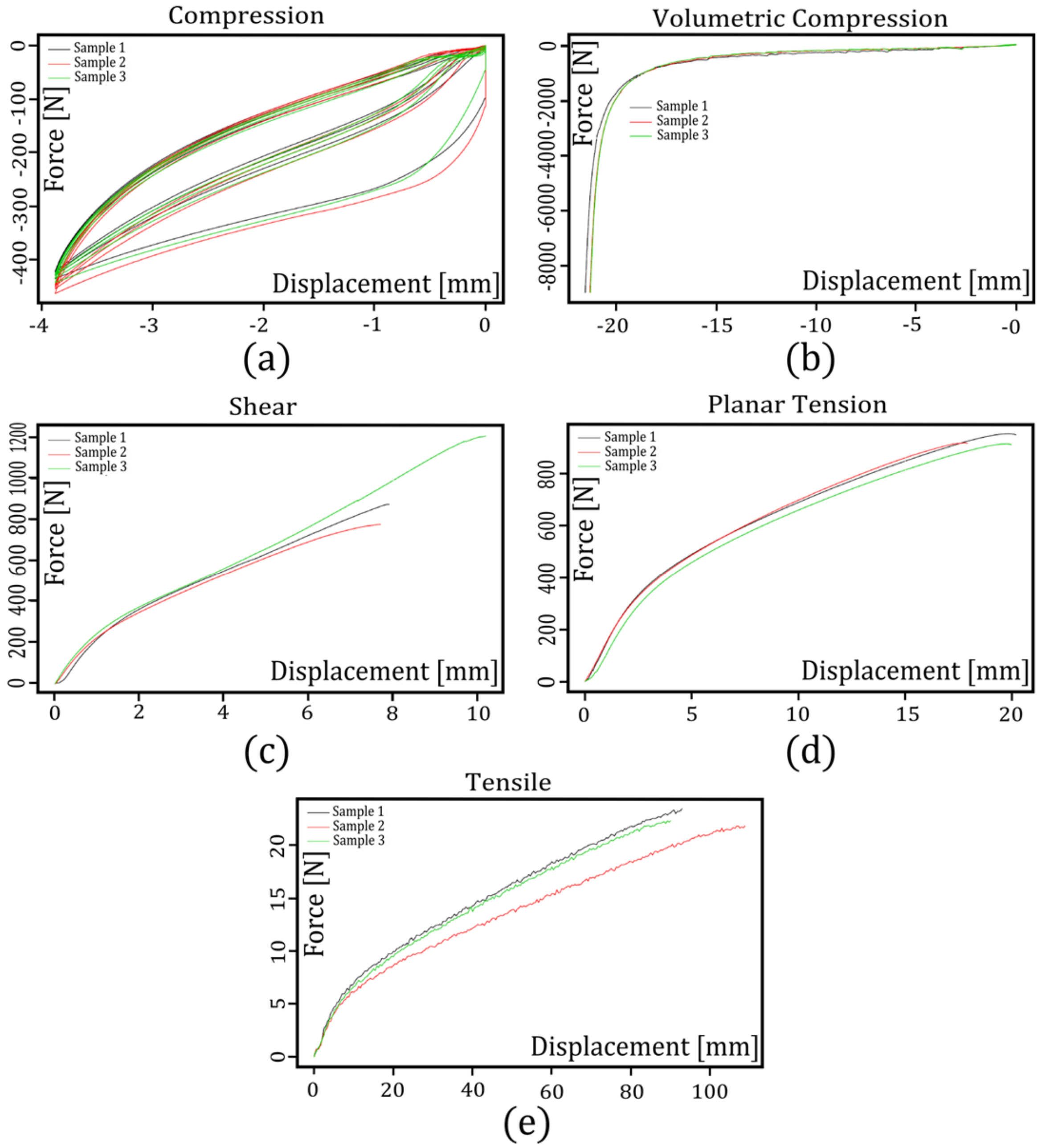

Figure S12. (**a**) Compression test for the EVA material; (**b**) Volumetric Compression test for the EVA material; (**c**) Shear test for the EVA material; (**d**) Planar Tension test for the EVA material, and (**e**) Tensile test for the EVA material.

S3.4.SBR Material

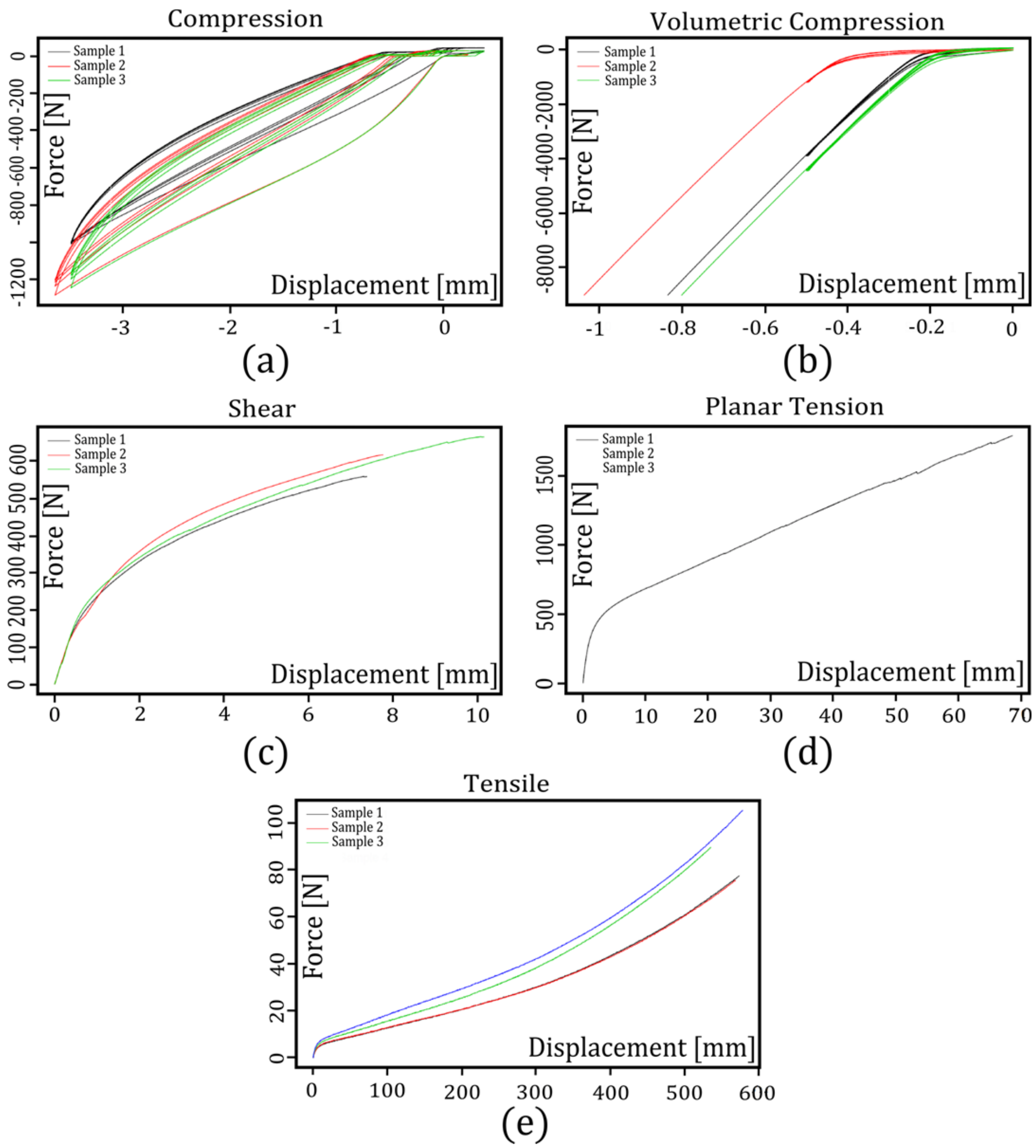

Figure S13. (**a**) Compression test for the SBR material; (**b**) Volumetric Compression test for the SBR material; (**c**) Shear test for the SBR material; (**d**) Planar Tension test for the SBR material, and (**e**) Tensile test for the SBR material.

S4. Design matrix and experiments

S4.1. NBR Material

Table S1. Design matrix and experiments obtained with a 5k DoE for the hyper-elastic Mooney-Rivlin model of the NBR material used in the Shear test.

	Inputs				*Output*
Sample	*C10* (x)	*C01* (x)	*C11* (x)	*Displacement* (mm)	*Force* (N)
1	-0.25	0.35	0.125	0.00	0.000
2	-0.25	0.35	0.125	0.50	12.117
3	-0.25	0.35	0.125	1.00	24.695
4	-0.25	0.35	0.125	1.50	38.228
5	-0.25	0.35	0.125	2.00	53.264
6	-0.25	0.35	0.125	2.50	70.419
7	-0.25	0.35	0.125	3.00	90.374
8	-0.25	0.35	0.125	3.50	113.829
9	-0.25	0.35	0.125	4.00	141.470
10	-0.25	0.35	0.125	4.50	173.949
11	-0.25	0.35	0.125	5.00	211.880
…	…	…	…	…	…
2623	1.25	1.00	0.500	9.00	7288.559
2624	1.25	1.00	0.500	9.50	7975.014
2625	1.25	1.00	0.500	10.00	8703.273

Table S2. Design matrix and experiments obtained with a 5k DoE for the hyper-elastic Mooney-Rivlin model of the NBR material used in the Volumetric Compression test.

	Inputs				*Output*
Sample	*C10* (x)	*C01* (x)	*C11* (x)	*Displacement* (mm)	*Force* (N)
1	-0.25	0.35	0.125	0.00	0.000
2	-0.25	0.35	0.125	-0.03	-325.281
3	-0.25	0.35	0.125	-0.06	-651.241
4	-0.25	0.35	0.125	-0.09	-977.877
5	-0.25	0.35	0.125	-0.12	-1305.190
6	-0.25	0.35	0.125	-0.15	-1633.190
7	-0.25	0.35	0.125	-0.18	-1961.870
8	-0.25	0.35	0.125	-0.21	-2291.230
9	-0.25	0.35	0.125	-0.24	-2621.280
10	-0.25	0.35	0.125	-0.27	-2952.030
11	-0.25	0.35	0.125	-0.30	-3283.460
…	…	…	…	…	…
2623	1.25	1.00	0.500	-0.54	-134101.000
2624	1.25	1.00	0.500	-0.57	-141699.000
2625	1.25	1.00	0.500	-0.60	-149314.000

Table S3. Design matrix and experiments obtained with a 5k DoE for the hyper-elastic Mooney-Rivlin model of the NBR material used in the Compression test.

	Inputs				***Output***
Sample	***C10* (x)**	***C01* (x)**	***C11* (x)**	***Displacement* (mm)**	***Force* (N)**
1	-0.25	0.35	0.125	0.000	0.000
2	-0.25	0.35	0.125	-0.195	-12.333
3	-0.25	0.35	0.125	-0.390	-27.024
4	-0.25	0.35	0.125	-0.585	-44.620
5	-0.25	0.35	0.125	-0.780	-65.800
6	-0.25	0.35	0.125	-0.975	-91.351
7	-0.25	0.35	0.125	-1.170	-122.190
8	-0.25	0.35	0.125	-1.365	-159.394
9	-0.25	0.35	0.125	-1.560	-204.234
10	-0.25	0.35	0.125	-1.755	-258.219
11	-0.25	0.35	0.125	-1.950	-323.140
…	…	…	…	…	…
2623	1.25	1.00	0.500	-3.510	-11696.200
2624	1.25	1.00	0.500	-3.705	-13446.200
2625	1.25	1.00	0.500	-3.900	-15478.300

Table S4. Design matrix and experiments obtained with a 5k DoE for the hyper-elastic Mooney-Rivlin model of the NBR material used in the Tensile test.

	Inputs				***Output***
Sample	***C10* (x)**	***C01* (x)**	***C11* (x)**	***Displacement* (mm)**	***Force* (N)**
1	-0.25	0.35	0.125	0	0.000
2	-0.25	0.35	0.125	20	1.317
3	-0.25	0.35	0.125	40	3.873
4	-0.25	0.35	0.125	60	8.745
5	-0.25	0.35	0.125	80	15.937
6	-0.25	0.35	0.125	100	25.338
7	-0.25	0.35	0.125	120	36.852
8	-0.25	0.35	0.125	140	50.402
9	-0.25	0.35	0.125	160	65.915
10	-0.25	0.35	0.125	180	83.320
11	-0.25	0.35	0.125	200	102.542
…	…	…	…	…	…
2623	1.25	1.00	0.500	360	1693.519
2624	1.25	1.00	0.500	380	1856.357
2625	1.25	1.00	0.500	400	2025.644

Table S5. Design matrix and experiments obtained with a 5k DoE for the hyper-elastic Mooney-Rivlin model of the NBR material used in the Planar Tension test.

	Inputs				***Output***
Sample	***C10*** **(x)**	***C01*** **(x)**	***C11*** **(x)**	***Displacement*** **(mm)**	***Force*** **(N)**
1	-0.25	0.35	0.125	0.00	0.000
2	-0.25	0.35	0.125	2.50	38.682
3	-0.25	0.35	0.125	5.00	76.905
4	-0.25	0.35	0.125	7.50	127.032
5	-0.25	0.35	0.125	10.00	196.563
6	-0.25	0.35	0.125	12.50	287.190
7	-0.25	0.35	0.125	15.00	399.426
8	-0.25	0.35	0.125	17.50	533.686
9	-0.25	0.35	0.125	20.00	690.317
10	-0.25	0.35	0.125	22.50	868.825
11	-0.25	0.35	0.125	25.00	1067.408
…	…	…	…	…	…
2623	1.25	1.00	0.500	45.00	21331.368
2624	1.25	1.00	0.500	47.50	23336.122
2625	1.25	1.00	0.500	50.00	25362.509

S4.2. PUR Material

Table S6. Design matrix and experiments obtained with a 5k DoE the hyper-elastic Ogden model of the PUR material used in the Shear test.

	Inputs			***Output***
Sample	***K1*** **(x)**	***K2*** **(x)**	***Displacement*** **(mm)**	***Force*** **(N)**
1	0.0	-0.50	0.00	0.000
2	0.0	-0.50	0.50	30.074
3	0.0	-0.50	1.00	60.111
4	0.0	-0.50	1.50	90.074
5	0.0	-0.50	2.00	119.923
6	0.0	-0.50	2.50	149.614
7	0.0	-0.50	3.00	179.101
8	0.0	-0.50	3.50	208.333
9	0.0	-0.50	4.00	237.252
10	0.0	-0.50	4.50	265.799
11	0.0	-0.50	5.00	293.908
…	…	…	…	…
523	0.5	0.0625	9.00	946.066
524	0.5	0.0625	9.50	1020.619
525	0.5	0.0625	10.00	1098.280

Table S7. Design matrix and experiments obtained with a 5k DoE the hyper-elastic Ogden model of the PUR material used in the Volumetric Compression test.

	Inputs			***Output***
Sample	***K1*** **(x)**	***K2*** **(x)**	***Displacement*** **(mm)**	***Force*** **(N)**
1	0.0	-0.50	0.00	0.000
2	0.0	-0.50	-0.03	-813.201
3	0.0	-0.50	-0.06	-1628.100
4	0.0	-0.50	-0.09	-2444.690
5	0.0	-0.50	-0.12	-3262.976
6	0.0	-0.50	-0.15	-4082.964
7	0.0	-0.50	-0.18	-4904.660
8	0.0	-0.50	-0.21	-5728.064
9	0.0	-0.50	-0.24	-6553.186
10	0.0	-0.50	-0.27	-7380.034
11	0.0	-0.50	-0.30	-8208.603
…	…	…	…	…
523	0.5	0.25	-0.54	-29795.848
524	0.5	0.25	-0.57	-31484.312
525	0.5	0.25	-0.60	-33176.336

Table S8. Design matrix and experiments obtained with a 5k DoE the hyper-elastic Ogden model of the PUR material used in the Compression test.

	Inputs			***Output***
Sample	***K1*** **(x)**	***K2*** **(x)**	***Displacement*** **(mm)**	***Force*** **(N)**
1	0.0	-0.50	0.000	0.000
2	0.0	-0.50	-0.195	-29.510
3	0.0	-0.50	-0.390	-61.337
4	0.0	-0.50	-0.585	-95.602
5	0.0	-0.50	-0.780	-132.479
6	0.0	-0.50	-0.975	-172.164
7	0.0	-0.50	-1.170	-214.874
8	0.0	-0.50	-1.365	-260.856
9	0.0	-0.50	-1.560	-310.397
10	0.0	-0.50	-1.755	-363.829
11	0.0	-0.50	-1.950	-421.540
…	…	…	…	…
523	0.5	0.25	-3.510	-652.503
524	0.5	0.25	-3.705	-710.302
525	0.5	0.25	-3.900	-772.318

Table S9. Design matrix and experiments obtained with a 5k DoE the hyper-elastic Ogden model of the PUR material used in the Tensile test.

	Inputs			***Output***
Sample	***K1*** **(x)**	***K2*** **(x)**	***Displacement*** **(mm)**	***Force*** **(N)**
1	0.0	-0.50	0	0.000
2	0.0	-0.50	20	2.987
3	0.0	-0.50	40	3.651
4	0.0	-0.50	60	3.850
5	0.0	-0.50	80	3.924
6	0.0	-0.50	100	3.957
7	0.0	-0.50	120	3.974
8	0.0	-0.50	140	3.983
9	0.0	-0.50	160	3.988
10	0.0	-0.50	180	3.991
11	0.0	-0.50	200	3.993
…	…	…	…	…
523	0.5	-0.125	360	291.280
524	0.5	-0.125	380	318.681
525	0.5	-0.125	400	347.177

Table S10. Design matrix and experiments obtained with a 5k DoE the hyper-elastic Ogden model of the PUR material used in the Planar Tension test.

	Inputs			***Output***
Sample	***K1* (x)**	***K2* (x)**	***Displacement* (mm)**	***Force* (N)**
1	0.0	-0.50	0.00	0
2	0.0	-0.50	2.50	94.471
3	0.0	-0.50	5.00	167.033
4	0.0	-0.50	7.50	224.882
5	0.0	-0.50	10.00	272.226
6	0.0	-0.50	12.50	311.708
7	0.0	-0.50	15.00	345.031
8	0.0	-0.50	17.50	373.317
9	0.0	-0.50	20.00	397.313
10	0.0	-0.50	22.50	414.668
11	0.0	-0.50	25.00	426.365
…	…	…	…	…
523	0.5	0.0625	45.00	2081.657
524	0.5	0.0625	47.50	2235.548
525	0.5	0.0625	50.00	2394.748

S4.3. SBR Material

Table S11. Design matrix and experiments obtained with a 5k DoE for the hyper-elastic Gent model of the SBR material used in the Shear test.

	Inputs			*Output*
Sample	***E*** **(x)**	***Inv*** **(x)**	***Displacement*** **(mm)**	***Force*** **(N)**
1	0.6	63.0	0.00	0.000
2	0.6	63.0	0.50	12.051
3	0.6	63.0	1.00	24.112
4	0.6	63.0	1.50	36.194
5	0.6	63.0	2.00	48.307
6	0.6	63.0	2.50	60.461
7	0.6	63.0	3.00	72.665
8	0.6	63.0	3.50	84.925
9	0.6	63.0	4.00	97.250
10	0.6	63.0	4.50	109.645
11	0.6	63.0	5.00	122.117
…	…	…	…	…
523	3.7	79.5	9.00	1375.126
524	3.7	79.5	9.50	1455.766
525	3.7	79.5	10.00	1536.961

Table S12. Design matrix and experiments obtained with a 5k DoE for the hyper-elastic Gent model of the SBR material used in the Volumetric Compression test.

	Inputs			*Output*
Sample	***E*** **(x)**	***Inv*** **(x)**	***Displacement*** **(mm)**	***Force*** **(N)**
1	0.6	63.0	0.00	0.000
2	0.6	63.0	-0.03	-325.280
3	0.6	63.0	-0.06	-651.240
4	0.6	63.0	-0.09	-977.875
5	0.6	63.0	-0.12	-1305.190
6	0.6	63.0	-0.15	-1633.184
7	0.6	63.0	-0.18	-1961.862
8	0.6	63.0	-0.21	-2291.223
9	0.6	63.0	-0.24	-2621.271
10	0.6	63.0	-0.27	-2952.011
11	0.6	63.0	-0.30	-3283.435
…	…	…	…	…
523	3.7	79.5	-0.54	-36753.288
524	3.7	79.5	-0.57	-38835.905
525	3.7	79.5	-0.60	-40923.022

Table S13. Design matrix and experiments obtained with a 5k DoE for the hyper-elastic Gent model of the SBR material used in the Compression test.

	Inputs			*Output*
Sample	*E* **(x)**	*Inv* **(x)**	*Displacement* **(mm)**	*Force* **(N)**
1	0.6	63.0	0.000	0.000
2	0.6	63.0	-0.195	-11.611
3	0.6	63.0	-0.390	-23.709
4	0.6	63.0	-0.585	-36.304
5	0.6	63.0	-0.780	-49.419
6	0.6	63.0	-0.975	-63.075
7	0.6	63.0	-1.170	-77.300
8	0.6	63.0	-1.365	-92.124
9	0.6	63.0	-1.560	-107.587
10	0.6	63.0	-1.755	-123.738
11	0.6	63.0	-1.950	-140.637
…	…	…	…	…
523	3.7	79.5	-3.510	-1943.177
524	3.7	79.5	-3.705	-2122.925
525	3.7	79.5	-3.900	-2323.115

Table S14. Design matrix and experiments obtained with a 5k DoE for the hyper-elastic Gent model of the SBR material used in the Tensile test.

	Inputs			*Output*
Sample	*E* **(x)**	*Inv* **(x)**	*Displacement* **(mm)**	*Force* **(N)**
1	0.6	63.0	0	0.000
2	0.6	63.0	20	1.729
3	0.6	63.0	40	2.965
4	0.6	63.0	60	4.112
5	0.6	63.0	80	5.282
6	0.6	63.0	100	6.530
7	0.6	63.0	120	7.891
8	0.6	63.0	140	9.401
9	0.6	63.0	160	11.100
10	0.6	63.0	180	13.036
11	0.6	63.0	200	15.274
…	…	…	…	…
523	3.7	79.5	360	288.649
524	3.7	79.5	380	354.913
525	3.7	79.5	400	510.715

Table S15. Design matrix and experiments obtained with a 5k DoE for the hyper-elastic Gent model of the SBR material used in the Planar Tension test.

	Inputs			***Output***
Sample	***E*** **(x)**	***Inv*** **(x)**	***Displacement*** **(mm)**	***Force*** **(N)**
1	0.6	63.0	0.00	0.000
2	0.6	63.0	2.50	38.367
3	0.6	63.0	5.00	68.960
4	0.6	63.0	7.50	94.539
5	0.6	63.0	10.00	116.749
6	0.6	63.0	12.50	136.654
7	0.6	63.0	15.00	154.962
8	0.6	63.0	17.50	172.163
9	0.6	63.0	20.00	188.600
10	0.6	63.0	22.50	204.523
11	0.6	63.0	25.00	220.119
…	…	…	…	…
523	3.7	79.5	45.00	2083.990
524	3.7	79.5	47.50	2179.894
525	3.7	79.5	50.00	2277.445

S4.4. EVA Material

Table S16. Design matrix and experiments obtained with a 3k DoE for the hyper-elastic Arruda-Boyce model of the EVA material used in the Shear test.

	Inputs			*Output*
Sample	*Nkt* **(x)**	*Chain* **(x)**	*Displacement* **(mm)**	*Force* **(N)**
1	0.26	2.0	0.00	0.000
2	0.26	2.0	0.50	24.332
3	0.26	2.0	1.00	48.792
4	0.26	2.0	1.50	73.511
5	0.26	2.0	2.00	98.624
6	0.26	2.0	2.50	124.269
7	0.26	2.0	3.00	150.593
8	0.26	2.0	3.50	177.753
9	0.26	2.0	4.00	205.918
10	0.26	2.0	4.50	235.267
…	…	…	…	…
187	0.90	25.0	9.00	1023.925
188	0.90	25.0	9.50	1083.246
189	0.90	25.0	10.00	1142.861

Table S17. Design matrix and experiments obtained with a 3k DoE for the hyper-elastic Arruda-Boyce model of the EVA material used in the Volumetric Compression test.

	Inputs			*Output*
Sample	*Nkt* **(x)**	*Chain* **(x)**	*Displacement* **(mm)**	*Force* **(N)**
1	0.26	2.0	0.00	0.000
2	0.26	2.0	-0.03	-422.927
3	0.26	2.0	-0.06	-846.737
4	0.26	2.0	-0.09	-1271.426
5	0.26	2.0	-0.12	-1696.997
6	0.26	2.0	-0.15	-2123.453
7	0.26	2.0	-0.18	-2550.796
8	0.26	2.0	-0.21	-2979.030
9	0.26	2.0	-0.24	-3408.156
10	0.26	2.0	-0.27	-3838.179
11	0.26	2.0	-0.30	-4269.097
…	…	…	…	…
187	0.90	25.0	-0.54	-26820.067
188	0.90	25.0	-0.57	-28340.198
189	0.90	25.0	-0.60	-29862.945

Table S18. Design matrix and experiments obtained with a 3k DoE for the hyper-elastic Arruda-Boyce model of the EVA material used in the Compression test.

	Inputs			***Output***
Sample	***Nkt* (x)**	***Chain* (x)**	***Displacement* (mm)**	***Force* (N)**
1	0.26	2.0	0.000	0.000
2	0.26	2.0	-0.195	-23.452
3	0.26	2.0	-0.390	-47.937
4	0.26	2.0	-0.585	-73.535
5	0.26	2.0	-0.780	-100.354
6	0.26	2.0	-0.975	-128.520
7	0.26	2.0	-1.170	-158.181
8	0.26	2.0	-1.365	-189.515
9	0.26	2.0	-1.560	-222.735
10	0.26	2.0	-1.755	-258.107
11	0.26	2.0	-1.950	-295.949
…	…	…	…	…
187	0.90	25.0	-3.510	-1448.051
188	0.90	25.0	-3.705	-1581.354
189	0.90	25.0	-3.900	-1729.455

Table S19. Design matrix and experiments obtained with a 3k DoE for the hyper-elastic Arruda-Boyce model of the EVA material used in the Tensile test.

	Inputs			***Output***
Sample	***Nkt* (x)**	***Chain* (x)**	***Displacement* (mm)**	***Force* (N)**
1	0.26	2.0	0	0.000
2	0.26	2.0	20	3.786
3	0.26	2.0	40	8.093
4	0.26	2.0	60	14.827
5	0.26	2.0	80	26.400
6	0.26	2.0	100	48.206
7	0.26	2.0	120	90.647
8	0.26	2.0	140	171.542
9	0.26	2.0	160	317.378
10	0.26	2.0	180	559.306
11	0.26	2.0	200	920.480
…	…	…	…	…
187	0.90	25.0	360	158.135
188	0.90	25.0	380	180.585
189	0.90	25.0	400	207.152

Table S20. Design matrix and experiments obtained with a 3k DoE for the hyper-elastic Arruda-Boyce model of the EVA material used in the Planar Tension test.

	Inputs			***Output***
Sample	***Nkt* (x)**	***Chain* (x)**	***Displacement* (mm)**	***Force* (N)**
1	0.26	2.0	0.00	0.000
2	0.26	2.0	2.50	77.970
3	0.26	2.0	5.00	142.607
4	0.26	2.0	7.50	200.861
5	0.26	2.0	10.00	257.125
6	0.26	2.0	12.50	314.693
7	0.26	2.0	15.00	376.396
8	0.26	2.0	17.50	444.990
9	0.26	2.0	20.00	523.425
10	0.26	2.0	22.50	615.058
11	0.26	2.0	25.00	723.855
…	…	…	…	…
187	0.90	25.0	45.00	1517.838
188	0.90	25.0	47.50	1583.657
189	0.90	25.0	50.00	1650.160

S5. Modeling the *EF* for the materials studied according to the standardized tests

S5.1 Gent hyper-elastic model for NBR.

$$EF_{shear} = 131.63192 + 573.60191 \cdot E - 727.3464 \cdot E^2 + 274.78272 \cdot E^3 - 31.8021 \cdot E^4 - 0.09809 \cdot E^2 \cdot I_1 + 0.0144 \cdot E^3 \cdot I_1 + 0.00179 \cdot E \cdot {I_1}^2 - 1e^{-5} \cdot E \cdot {I_1}^3$$

$$EF_{comVol} = 1691.91316 - 4218.6483 \cdot E + 4467.51432 \cdot E^2 - 1221.19525 \cdot E^3 + 120.3148 \cdot E^4 - 4e^{-5} \cdot E \cdot I_1$$

$$EF_{comp} = 162.59816 + 506.39271 \cdot E - 745.52302 \cdot E^2 + 306.56328 \cdot E^3 - 37.36569 \cdot E^4 - 0.07396 \cdot E^2 \cdot I_1 + 0.01082 \cdot E^3 \cdot I_1 + 0.00135 \cdot E \cdot {I_1}^2$$

$$EF_{trac} = -0.04142 + 42.93096 \cdot E - 52.92906 \cdot E^2 + 23.18558 \cdot E^3 - 2.98271 \cdot E^4 + 0.3083 \cdot I_1 - 0.21959 \cdot E^2 \cdot I_1 + 0.03988 \cdot E^3 \cdot I_1 - 0.00331 \cdot {I_1}^2 + 0.00336 \cdot E \cdot {I_1}^2 - 0.00042 \cdot E^2 \cdot {I_1}^2$$

$$EF_{tens} = 570.06975 - 148.80814 \cdot E + 0.16335 \cdot I_1 + 0.10503 \cdot E \cdot I_1 - 0.00346 \cdot {I_1}^2 - 0.00101 \cdot E \cdot {I_1}^2 + 3e^{-5} \cdot {I_1}^3$$

S5.2 Gent hyper-elastic model for EVA.

$$EF_{shear} = 573.42844 - 384.8863 \cdot E + 239.92652 \cdot E^2 - 100.16773 \cdot E^3 + 14.66798 \cdot E^4 + 0.06727 \cdot E \cdot I_1 - 0.0274 \cdot E^2 \cdot I_1 - 0.00018 \cdot {I_1}^2 + 5e^{-5} \cdot E^2 \cdot {I_1}^2$$

$$EF_{comVol} = 707.60358 - 1888.22615 \cdot E + 2264.06588 \cdot E^2 - 618.88252 \cdot E^3 + 60.97365 \cdot E^4$$

$$EF_{comp} = -84.84631 + 197.34822 \cdot E - 0.00027 \cdot E^3 - 0.99611 \cdot I_1 - 0.10962 \cdot E \cdot I_1 - 2e^{-5} \cdot E^2 \cdot I_1 + 0.0211 \cdot {I_1}^2 + 0.00113 \cdot E \cdot {I_1}^2 - 2e^{-4} \cdot {I_1}^3$$

$$EF_{trac} = 8.39199 + 5.45142 \cdot E - 10.56627 \cdot E^2 + 3.88764 \cdot E^3 - 0.40133 \cdot E^4 + 0.03933 \cdot E \cdot I_1 - 0.01962 \cdot E^2 \cdot I_1 + 0.00245 \cdot E^3 \cdot I_1 - 0.00011 \cdot {I_1}^2$$

$$EF_{tens} = 562.03356 - 266.68752 \cdot E + 121.89898 \cdot E^2 - 50.84421 \cdot E^3 + 7.40561 \cdot E^4 + 0.0892 \cdot E \cdot I_1 + 0.04214 \cdot E^2 \cdot I_1 - 0.01916 \cdot E^3 \cdot I_1 - 0.00153 \cdot E \cdot {I_1}^2 + 0.00039 \cdot E^2 \cdot {I_1}^2$$

S5.3 Gent hyper-elastic model for PUR.

$$EF_{shear} = 952.07301 - 167.72434 \cdot E + 0.29917 \cdot E^2 - 0.0081 \cdot E^3 + 0.00019 \cdot E^4 + 0.19236 \cdot I_1 + 0.12239 \cdot E \cdot I_1 - 0.00033 \cdot E^2 \cdot I_1 - 0.00406 \cdot {I_1}^2 - 0.00117 \cdot E \cdot {I_1}^2 + 4e^{-5} \cdot {I_1}^3$$

$$EF_{comVol} = -131.47184 - 379.4103 \cdot E + 1998.31163 \cdot E^2 - 546.23856 \cdot E^3 + 53.8166 \cdot E^4 - 4e^{-5} \cdot E \cdot I_1$$

$$EF_{comp} = 768.25364 - 211.78034 \cdot E + 13.88159 \cdot E^2 - 4.97277 \cdot E^3 + 0.64769 \cdot E^4 + 0.99605 \cdot I_1 + 0.10973 \cdot E \cdot I_1 - 2e^{-5} \cdot E^3 \cdot I_1 - 0.0211 \cdot {I_1}^2 - 0.00113 \cdot E \cdot {I_1}^2 + 2e^{-4} \cdot {I_1}^3$$

$$EF_{trac} = 44.14595 - 26.35027 \cdot E + 6.04914 \cdot E^2 - 0.93071 \cdot E^3 + 0.29402 \cdot E^4 + 0.20345 \cdot E \cdot I_1 - 0.09803 \cdot E^2 \cdot I_1 - 5e^{-4} \cdot {I_1}^2 + 0.00025 \cdot E^2 \cdot {I_1}^2$$

$$EF_{tens} = 1150.10935 - 128.73997 \cdot E + 0.10502 \cdot I_1 + 0.06796 \cdot E \cdot I_1 - 0.00222 \cdot {I_1}^2 - 0.00065 \cdot E \cdot {I_1}^2 + 2e^{-5} \cdot {I_1}^3$$

S5.4 Gent hyper-elastic model for SBR

$$EF_{shear} = 245.33787 + 315.03982 \cdot E - 440.56609 \cdot E^2 + 159.41681 \cdot E^3 - 17.48769 \cdot E^4 + 0.1115 \cdot E \cdot I_1 - 0.07149 \cdot E^2 \cdot I_1 + 0.0083 \cdot E^3 \cdot I_1 - 0.00028 \cdot {I_1}^2 + 7e^{-5} \cdot E^2 \cdot {I_1}^2$$

$$EF_{comVol} = 1092.51337 - 2967.75314 \cdot E + 3663.00216 \cdot E^2 - 1001.28181 \cdot E^3 + 98.64845 \cdot E^4 - 4e^{-5} \cdot E \cdot I_1$$

$$EF_{comp} = 499.8732 - 768.75451 \cdot E + 420.72193 \cdot E^2 - 76.30303 \cdot E^3 + 4.75934 \cdot E^4 - 0.06616 \cdot E^2 \cdot I_1 + 0.0102 \cdot E^3 \cdot I_1 + 0.00107 \cdot E \cdot {I_1}^2$$

$$EF_{trac} = 13.46596 - 31.84001 \cdot E + 41.66887 \cdot E^2 - 12.0611 \cdot E^3 + 1.22107 \cdot E^4 + 0.39297 \cdot I_1 - 0.82184 \cdot E \cdot I_1 + 0.11071 \cdot E^2 \cdot I_1 - 0.00888 \cdot E^3 \cdot I_1 - 0.00172 \cdot {I_1}^2 + 0.00588 \cdot E \cdot {I_1}^2 - 0.00028 \cdot E^2 \cdot {I_1}^2 - 2e^{-5} \cdot E \cdot {I_1}^3$$

$$EF_{tens} = 602.16882 - 317.01575 \cdot E + 173.83253 \cdot E^2 - 72.20933 \cdot E^3 + 10.43612 \cdot E^4 + 0.0892 \cdot E \cdot I_1 + 0.04214 \cdot E^2 \cdot I_1 - 0.01916 \cdot E^3 \cdot I_1 - 0.00153 \cdot E \cdot {I_1}^2 + 0.00039 \cdot E^2 \cdot {I_1}^2$$

S5.5 Ogden hyper-elastic model for NBR.

$$EF_{shear} = 367.42734 - 5152.96075 \cdot k_1{}^2 + 7890.83881 \cdot k_1{}^3 + 463.85768 \cdot k_2 + 6119.73291 \cdot k_1 \cdot k_2 - 28912.28113 \cdot k_1{}^2 \cdot k_2 + 26531.08548 \cdot k_1{}^3 \cdot k_2 + 6907.1563 \cdot k_1 \cdot k_2{}^2 - 13594.22433 \cdot k_1{}^2 \cdot k_2{}^2$$

$$EF_{comVol} = 2581.62332 - 37573.18872 \cdot k_1 + 170279.87916 \cdot k_1{}^2 - 241796.12966 \cdot k_1{}^3 + 127131.93523 \cdot k_1{}^4 + 15621.60234 \cdot k_2 - 195859.04111 \cdot k_1 \cdot k_2 + 432669.69466 \cdot k_1{}^2 \cdot k_2 - 262089.57722 \cdot k_1{}^3 \cdot k_2 + 28541.94027 \cdot k_2{}^2 - 197711.69582 \cdot k_1 \cdot k_2{}^2 + 290587.69511 \cdot k_1{}^2 \cdot k_2{}^2$$

$$EF_{comp} = 366.68459 - 5078.88291 \cdot k_1{}^2 + 7328.67562 \cdot k_1{}^3 + 690.89965 \cdot k_2 + 8733.43345 \cdot k_1 \cdot k_2 - 36736.94016 \cdot k_1{}^2 \cdot k_2 + 23907.53842 \cdot k_1{}^3 \cdot k_2 + 17779.24803 \cdot k_1 \cdot k_2{}^2 - 38272.40791 \cdot k_1{}^2 \cdot k_2{}^2$$

$$EF_{trac} = 28.5913 - 39.96472 \cdot k_1 - 1108.94844 \cdot k_1{}^2 + 4691.16536 \cdot k_1{}^3 - 4660.30136 \cdot k_1{}^4 + 7.36883 \cdot k_2 + 90.62453 \cdot k_1 \cdot k_2 - 726.94527 \cdot k_1{}^2 \cdot k_2 + 946.88324 \cdot k_1{}^3 \cdot k_2$$

$$EF_{tens} = 573.2217 - 852.02857 \cdot k_1 + 931.70282 \cdot k_1{}^2 - 1982.04384 \cdot k_1{}^3 + 1462.31866 \cdot k_1{}^4 + 405.23114 \cdot k_2 - 537.63144 \cdot k_1 \cdot k_2 + 2806.32998 \cdot k_1{}^2 \cdot k_2 - 2434.71154 \cdot k_1{}^3 \cdot k_2 - 688.13911 \cdot k_1 \cdot k_2{}^2 + 2500.09091 \cdot k_1{}^2 \cdot k_2{}^2$$

S5.6 Ogden hyper-elastic model for EVA.

$$EF_{shear} = 500.26043 - 522.16823 \cdot k_1 - 448.26096 \cdot k_1{}^2 + 428.51384 \cdot k_2 + 1473.1078 \cdot k_1 \cdot k_2 - 5850.38214 \cdot k_1{}^3 \cdot k_2 + 1789.74701 \cdot k_1 \cdot k_2{}^2$$

$$EF_{comVol} = 1616.69292 - 20415.08222 \cdot k_1 + 94735.17245 \cdot k_1{}^2 - 134648.98076 \cdot k_1{}^3 + 71812.79252 \cdot k_1{}^4 + 11290.79012 \cdot k_2 - 109322.1388 \cdot k_1 \cdot k_2 + 241085.77243 \cdot k_1{}^2 \cdot k_2 - 143625.56931 \cdot k_1{}^3 \cdot k_2 + 26557.57604 \cdot k_2{}^2 - 110796.86661 \cdot k_1 \cdot k_2{}^2 + 164143.50291 \cdot k_1{}^2 \cdot k_2{}^2 + 11366.58535 \cdot k_2{}^3$$

$$EF_{comp} = -49.94918 + 327.64699 \cdot k_1 + 1854.38526 \cdot k_1{}^2 - 2647.32065 \cdot k_1{}^3 + 1392.55523 \cdot k_1{}^4 - 455.54137 \cdot k_2 - 2154.13915 \cdot k_1 \cdot k_2 + 4693.37501 \cdot k_1{}^2 \cdot k_2 - 2901.87881 \cdot k_1{}^3 \cdot k_2 + 315.00427 \cdot k_2{}^2 - 2148.82615 \cdot k_1 \cdot k_2{}^2 + 3050.43338 \cdot k_1{}^2 \cdot k_2{}^2$$

$$EF_{trac} = 11.61421 - 323.2176 \cdot {k_1}^2 + 967.3286 \cdot {k_1}^3 - 763.38933 \cdot {k_1}^4 + 66.26786 \cdot k_1 \cdot k_2 - 324.0273 \cdot {k_1}^2 \cdot k_2 + 337.80496 \cdot {k_1}^3 \cdot k_2 - 19.25412 \cdot {k_2}^2 + 19.65498 \cdot k_1 \cdot {k_2}^2 - 19.7614 \cdot {k_2}^3$$

$$EF_{tens} = 509.74359 - 1227.96208 \cdot k_1 + 3648.21224 \cdot {k_1}^2 - 8322.9179 \cdot {k_1}^3 + 6379.72333 \cdot {k_1}^4 + 165.31536 \cdot k_2 - 2404.17253 \cdot k_1 \cdot k_2 + 11705.84823 \cdot {k_1}^2 \cdot k_2 - 11653.71925 \cdot {k_1}^3 \cdot k_2 - 979.87191 \cdot {k_2}^2 - 2817.5445 \cdot k_1 \cdot {k_2}^2 + 9306.58759 \cdot {k_1}^2 \cdot {k_2}^2 - 1127.70583 \cdot {k_2}^3$$

S5.7 Ogden hyper-elastic model for PUR.

$$EF_{shear} = 955.53103 - 824.92775 \cdot k_1 + 135.92408 \cdot {k_1}^2 - 286.42552 \cdot {k_1}^3 + 212.07954 \cdot {k_1}^4 + 466.62242 \cdot k_2 + 96.85461 \cdot k_1 \cdot k_2 - 130.62892 \cdot {k_1}^2 \cdot k_2 + 23.53591 \cdot {k_1}^3 \cdot k_2 + 2.73597 \cdot {k_2}^2 + 77.41648 \cdot k_1 \cdot {k_2}^2 - 112.54282 \cdot {k_1}^2 \cdot {k_2}^2$$

$$EF_{comVol} = 1614.5898 - 24195.9722 \cdot k_1 + 124627.01377 \cdot {k_1}^2 - 176908.8083 \cdot {k_1}^3 + 92525.36384 \cdot {k_1}^4 + 9233.29106 \cdot k_2 - 143176.71591 \cdot k_1 \cdot k_2 + 316490.4907 \cdot {k_1}^2 \cdot k_2 - 192876.43444 \cdot {k_1}^3 \cdot k_2 + 20879.0566 \cdot {k_2}^2 - 144318.69996 \cdot k_1 \cdot {k_2}^2 + 211486.9605 \cdot {k_1}^2 \cdot {k_2}^2$$

$$EF_{comp} = 781.24808 - 1097.85589 \cdot k_1 + 1549.97414 \cdot {k_1}^2 - 3172.75346 \cdot {k_1}^3 + 2263.64834 \cdot {k_1}^4 + 718.03261 \cdot k_2 - 1127.8068 \cdot k_1 \cdot k_2 + 5448.10541 \cdot {k_1}^2 \cdot k_2 - 4734.37263 \cdot {k_1}^3 \cdot k_2 - 1293.98871 \cdot k_1 \cdot {k_2}^2 + 4668.65913 \cdot {k_1}^2 \cdot {k_2}^2$$

$$EF_{trac} = 41.98032 - 27.79486 \cdot k_1 - 1014.51265 \cdot {k_1}^2 + 3444.69074 \cdot {k_1}^3 - 2978.76489 \cdot {k_1}^4 + 6.74623 \cdot k_2 + 88.20951 \cdot k_1 \cdot k_2 - 444.93251 \cdot {k_1}^2 \cdot k_2 + 439.18303 \cdot {k_1}^3 \cdot k_2$$

$$EF_{tens} = 1151.96645 - 620.98687 \cdot k_1 + 65.71428 \cdot {k_1}^2 - 158.74504 \cdot {k_1}^3 + 125.63622 \cdot {k_1}^4 + 356.77436 \cdot k_2 + 56.62993 \cdot k_1 \cdot k_2 - 91.02943 \cdot {k_1}^2 \cdot k_2 + 36.80589 \cdot {k_1}^3 \cdot k_2 + 37.37657 \cdot k_1 \cdot {k_2}^2 - 57.92156 \cdot {k_1}^2 \cdot {k_2}^2$$

S5.8 Ogden hyper-elastic model for SBR.

$$EF_{shear} = 393.17542 - 4029.63965 \cdot {k_1}^2 + 5646.97637 \cdot {k_1}^3 + 404.53928 \cdot k_2 + 4315.4355 \cdot k_1 \cdot k_2 - 18475.87259 \cdot {k_1}^2 \cdot k_2 + 15758.0234 \cdot {k_1}^3 \cdot k_2 + 4396.72026 \cdot k_1 \cdot {k_2}^2 - 7812.45505 \cdot {k_1}^2 \cdot {k_2}^2$$

$$EF_{comVol} = 2283.41882 - 33448.05129 \cdot k_1 + 156201.88741 \cdot {k_1}^2 - 221786.80128 \cdot {k_1}^3 + 116460.29343 \cdot {k_1}^4 + 13651.63632 \cdot k_2 - 179613.37212 \cdot k_1 \cdot k_2 + 396843.46861 \cdot {k_1}^2 \cdot k_2 - 240746.29362 \cdot {k_1}^3 \cdot k_2 + 26178.93387 \cdot {k_2}^2 - 181246.87704 \cdot k_1 \cdot {k_2}^2 + 266195.371 \cdot {k_1}^2 \cdot {k_2}^2$$

$$EF_{comp} = 379.29256 - 2475.54838 \cdot k_1 + 4535.18305 \cdot {k_1}^2 - 2294.96025 \cdot {k_1}^4 + 1654.31068 \cdot k_2 - 8636.14369 \cdot k_1 \cdot k_2 + 7076.52587 \cdot {k_1}^2 \cdot k_2 + 2097.40454 \cdot {k_2}^2 - 4798.09832 \cdot k_1 \cdot {k_2}^2$$

$$EF_{trac} = 15.26002 - 279.76921 \cdot k_1 + 1926.94633 \cdot {k_1}^2 - 4137.57559 \cdot {k_1}^3 + 3194.13256 \cdot {k_1}^4 + 7.12619 \cdot k_2 - 83.52135 \cdot k_1 \cdot k_2 + 165.49685 \cdot {k_1}^2 \cdot k_2 - 149.96712 \cdot {k_1}^3 \cdot k_2 + 42.69854 \cdot k_1 \cdot {k_2}^2 - 92.17532 \cdot {k_1}^2 \cdot {k_2}^2$$

$$EF_{tens} = 549.61218 - 903.07119 \cdot k_1 + 1870.26214 \cdot {k_1}^2 - 4987.2196 \cdot {k_1}^3 + 3921.60778 \cdot {k_1}^4 + 405.05656 \cdot k_2 - 652.1197 \cdot k_1 \cdot k_2 + 6073.58795 \cdot {k_1}^2 \cdot k_2 - 8972.76722 \cdot {k_1}^3 \cdot k_2 - 572.35034 \cdot k_1 \cdot {k_2}^2 + 3938.94766 \cdot {k_1}^2 \cdot {k_2}^2$$

S5.9 Mooney-Rivlin hyper-elastic model for NBR.

$$
\begin{aligned}
EF_{shear} = \; & 183.52116 - 693.53046 \cdot C_{10} + 1400.42875 \cdot {C_{10}}^2 - 278.43835 \cdot {C_{10}}^4 \\
& - 640.16174 \cdot C_{01} + 3456.7162 \cdot C_{10} \cdot C_{01} - 2482.67744 \cdot {C_{10}}^2 \cdot C_{01} \\
& + 609.80965 \cdot {C_{10}}^3 \cdot C_{01} + 1443.64739 \cdot {C_{01}}^2 - 2414.77072 \cdot C_{10} \cdot {C_{01}}^2 \\
& + 802.19476 \cdot {C_{10}}^2 \cdot {C_{01}}^2 + 570.90191 \cdot C_{10} \cdot {C_{01}}^3 - 429.79401 \cdot {C_{01}}^4 \\
& + 2053.13932 \cdot C_{10} \cdot C_{11} - 882.06251 \cdot {C_{10}}^2 \cdot C_{11} + 1163.36567 \cdot C_{01} \\
& \cdot C_{11} - 1288.34095 \cdot C_{10} \cdot C_{01} \cdot C_{11} + 911.5076 \cdot {C_{11}}^2 - 3050.22947 \\
& \cdot C_{10} \cdot {C_{11}}^2 + 1662.59232 \cdot {C_{10}}^2 \cdot {C_{11}}^2 - 1734.73345 \cdot C_{01} \cdot {C_{11}}^2 \\
& + 1520.55779 \cdot C_{10} \cdot C_{01} \cdot {C_{11}}^2 + 559.5721 \cdot {C_{01}}^2 \cdot {C_{11}}^2
\end{aligned}
$$

$$
\begin{aligned}
EF_{comVol} = \; & -1670.36632 + 12736.21437 \cdot C_{10} + 7602.62765 \cdot {C_{10}}^2 - 6175.18798 \\
& \cdot {C_{10}}^3 + 1711.70271 \cdot {C_{10}}^4 + 14977.88218 \cdot C_{01} + 6784.19505 \cdot C_{10} \\
& \cdot C_{01} - 8314.5558 \cdot {C_{10}}^2 \cdot C_{01} + 3142.55075 \cdot {C_{10}}^3 \cdot C_{01} + 1639.17496 \\
& \cdot {C_{01}}^2 - 2722.01456 \cdot C_{10} \cdot {C_{01}}^2 + 1535.43174 \cdot {C_{10}}^2 \cdot {C_{01}}^2 \\
& - 2502.70944 \cdot {C_{01}}^3 + 1707.68537 \cdot {C_{01}}^4
\end{aligned}
$$

$$
\begin{aligned}
EF_{comp} = \; & 103.20989 + 879.48322 \cdot {C_{10}}^2 - 214.64751 \cdot {C_{10}}^3 - 253.99821 \cdot C_{01} \\
& + 2910.10452 \cdot C_{10} \cdot C_{01} - 1551.76327 \cdot {C_{10}}^2 \cdot C_{01} + 245.33878 \cdot {C_{10}}^3 \\
& \cdot C_{01} + 1903.78218 \cdot {C_{01}}^2 - 2151.15598 \cdot C_{10} \cdot {C_{01}}^2 + 580.48796 \cdot {C_{10}}^2 \\
& \cdot {C_{01}}^2 - 700.25323 \cdot {C_{01}}^3 + 471.55589 \cdot C_{10} \cdot {C_{01}}^3 - 698.90049 \cdot C_{11} \\
& + 2372.54385 \cdot C_{10} \cdot C_{11} - 760.1869 \cdot {C_{10}}^2 \cdot C_{11} + 4184.24278 \cdot C_{01} \\
& \cdot C_{11} - 4217.4931 \cdot C_{10} \cdot C_{01} \cdot C_{11} + 924.87521 \cdot {C_{10}}^2 \cdot C_{01} \cdot C_{11} \\
& - 3026.17171 \cdot {C_{01}}^2 \cdot C_{11} + 1482.38309 \cdot C_{10} \cdot {C_{01}}^2 \cdot C_{11} + 619.29957 \\
& \cdot {C_{01}}^3 \cdot C_{11} + 1525.29295 \cdot {C_{11}}^2 - 1444.2328 \cdot C_{10} \cdot {C_{11}}^2 - 3155.93335 \\
& \cdot C_{01} \cdot {C_{11}}^2 + 1841.99624 \cdot C_{10} \cdot C_{01} \cdot {C_{11}}^2 + 1312.51796 \cdot {C_{01}}^2 \cdot {C_{11}}^2
\end{aligned}
$$

$$
\begin{aligned}
EF_{trac} = \; & 19.3254 - 36.88252 \cdot C_{10} + 22.88505 \cdot {C_{10}}^2 + 45.20189 \cdot {C_{10}}^3 - 21.76448 \\
& \cdot {C_{10}}^4 - 25.85826 \cdot C_{01} + 66.5464 \cdot C_{10} \cdot C_{01} - 23.43407 \cdot {C_{10}}^2 \cdot C_{01} \\
& + 13.52604 \cdot {C_{01}}^2 - 12.79691 \cdot C_{10} \cdot {C_{01}}^2 - 46.10194 \cdot C_{11} + 733.56847 \\
& \cdot C_{10} \cdot C_{11} - 256.65739 \cdot {C_{10}}^2 \cdot C_{11} + 305.5666 \cdot C_{01} \cdot C_{11} - 310.44061 \\
& \cdot C_{10} \cdot C_{01} \cdot C_{11} + 79.32516 \cdot {C_{10}}^2 \cdot C_{01} \cdot C_{11} - 53.26243 \cdot {C_{01}}^2 \cdot C_{11} \\
& + 48.06354 \cdot C_{10} \cdot {C_{01}}^2 \cdot C_{11} + 1561.50985 \cdot {C_{11}}^2 - 1585.0807 \cdot C_{10} \\
& \cdot {C_{11}}^2 + 289.82983 \cdot {C_{10}}^2 \cdot {C_{11}}^2 - 507.29949 \cdot C_{01} \cdot {C_{11}}^2 + 206.29549 \\
& \cdot C_{10} \cdot C_{01} \cdot {C_{11}}^2 - 2327.54218 \cdot {C_{11}}^3 + 1199.70524 \cdot C_{10} \cdot {C_{11}}^3 \\
& + 357.56136 \cdot C_{01} \cdot {C_{11}}^3 + 1177.73578 \cdot {C_{11}}^4
\end{aligned}
$$

$$
\begin{aligned}
EF_{tens} = {} & 832.38853 - 2928.96076 \cdot C_{10} + 3625.24508 \cdot {C_{10}}^2 - 1474.22101 \cdot {C_{10}}^3 \\
& + 222.8628 \cdot {C_{10}}^4 - 2756.07978 \cdot C_{01} + 7012.25395 \cdot C_{10} \cdot C_{01} \\
& - 4311.33566 \cdot {C_{10}}^2 \cdot C_{01} + 834.43158 \cdot {C_{10}}^3 \cdot C_{01} + 3232.13812 \cdot {C_{01}}^2 \\
& - 4100.53672 \cdot C_{10} \cdot {C_{01}}^2 + 1221.59319 \cdot {C_{10}}^2 \cdot {C_{01}}^2 - 1261.07643 \\
& \cdot {C_{01}}^3 + 794.24004 \cdot C_{10} \cdot {C_{01}}^3 + 175.17826 \cdot {C_{01}}^4 - 2182.45536 \cdot C_{11} \\
& + 5775.64572 \cdot C_{10} \cdot C_{11} - 3444.18044 \cdot {C_{10}}^2 \cdot C_{11} + 638.65362 \cdot {C_{10}}^3 \\
& \cdot C_{11} + 5064.22906 \cdot C_{01} \cdot C_{11} - 6767.25387 \cdot C_{10} \cdot C_{01} \cdot C_{11} \\
& + 2064.88617 \cdot {C_{10}}^2 \cdot C_{01} \cdot C_{11} - 2433.42779 \cdot {C_{01}}^2 \cdot C_{11} + 1750.19183 \\
& \cdot C_{10} \cdot {C_{01}}^2 \cdot C_{11} + 383.5516 \cdot {C_{01}}^3 \cdot C_{11} + 2132.25963 \cdot {C_{11}}^2 \\
& - 2701.53369 \cdot C_{10} \cdot {C_{11}}^2 + 751.67271 \cdot {C_{10}}^2 \cdot {C_{11}}^2 - 2051.35937 \cdot C_{01} \\
& \cdot {C_{11}}^2 + 1714.98241 \cdot C_{10} \cdot C_{01} \cdot {C_{11}}^2
\end{aligned}
$$

S5.10 Mooney-Rivlin hyper-elastic model for EVA.

$$
\begin{aligned}
EF_{shear} = {} & 595.96368 - 2178.67359 \cdot C_{10} + 2865.80232 \cdot {C_{10}}^2 - 883.96588 \cdot {C_{10}}^3 \\
& - 2098.17268 \cdot C_{01} + 5966.93267 \cdot C_{10} \cdot C_{01} - 3807.74099 \cdot {C_{10}}^2 \cdot C_{01} \\
& + 775.44103 \cdot {C_{10}}^3 \cdot C_{01} + 2701.24867 \cdot {C_{01}}^2 - 3650.48531 \cdot C_{10} \cdot {C_{01}}^2 \\
& + 1120.27509 \cdot {C_{10}}^2 \cdot {C_{01}}^2 - 831.78589 \cdot {C_{01}}^3 + 716.71532 \cdot C_{10} \cdot {C_{01}}^3 \\
& - 1379.9238 \cdot C_{11} + 4679.20347 \cdot C_{10} \cdot C_{11} - 2862.35422 \cdot {C_{10}}^2 \cdot C_{11} \\
& + 472.44967 \cdot {C_{10}}^3 \cdot C_{11} + 4200.79571 \cdot C_{01} \cdot C_{11} - 5431.76083 \cdot C_{10} \\
& \cdot C_{01} \cdot C_{11} + 1635.15355 \cdot {C_{10}}^2 \cdot C_{01} \cdot C_{11} - 1954.13109 \cdot {C_{01}}^2 \cdot C_{11} \\
& + 1355.94807 \cdot C_{10} \cdot {C_{01}}^2 \cdot C_{11} + 1658.44324 \cdot {C_{11}}^2 - 3098.96029 \cdot C_{10} \\
& \cdot {C_{11}}^2 + 1264.53916 \cdot {C_{10}}^2 \cdot {C_{11}}^2 - 2686.18451 \cdot C_{01} \cdot {C_{11}}^2 \\
& + 1886.83392 \cdot C_{10} \cdot C_{01} \cdot {C_{11}}^2 + 996.8374 \cdot {C_{01}}^2 \cdot {C_{11}}^2
\end{aligned}
$$

$$
\begin{aligned}
EF_{comVol} = {} & -996.34559 + 7952.81782 \cdot C_{10} + 3852.90706 \cdot {C_{10}}^2 - 3129.4986 \cdot {C_{10}}^3 \\
& + 867.46646 \cdot {C_{10}}^4 + 9088.86153 \cdot C_{01} + 3438.14364 \cdot C_{10} \cdot C_{01} \\
& - 4213.70757 \cdot {C_{10}}^2 \cdot C_{01} + 1592.60195 \cdot {C_{10}}^3 \cdot C_{01} + 830.72076 \cdot {C_{01}}^2 \\
& - 1379.48519 \cdot C_{10} \cdot {C_{01}}^2 + 778.13975 \cdot {C_{10}}^2 \cdot {C_{01}}^2 - 1268.35574 \cdot {C_{01}}^3 \\
& + 865.44065 \cdot {C_{01}}^4
\end{aligned}
$$

$$
\begin{aligned}
EF_{comp} = {} & -104.02636 + 1163.7845 \cdot C_{10} - 11.39835 \cdot {C_{10}}^2 + 8.05562 \cdot {C_{10}}^3 \\
& - 2.05642 \cdot {C_{10}}^4 + 1451.80406 \cdot C_{01} - 19.85195 \cdot C_{10} \cdot C_{01} + 17.24875 \\
& \cdot {C_{10}}^2 \cdot C_{01} - 5.46286 \cdot {C_{10}}^3 \cdot C_{01} - 28.52999 \cdot {C_{01}}^2 + 22.90205 \cdot C_{10} \\
& \cdot {C_{01}}^2 - 6.84659 \cdot {C_{10}}^2 \cdot {C_{01}}^2 + 21.90531 \cdot {C_{01}}^3 - 6.66201 \cdot C_{10} \cdot {C_{01}}^3 \\
& - 6.37661 \cdot {C_{01}}^4 + 912.21997 \cdot C_{11} + 65.09823 \cdot C_{10} \cdot C_{11} - 26.84801 \\
& \cdot {C_{10}}^2 \cdot C_{11} + 5.79171 \cdot {C_{10}}^3 \cdot C_{11} + 87.65009 \cdot C_{01} \cdot C_{11} - 68.45968 \cdot C_{10} \\
& \cdot C_{01} \cdot C_{11} + 18.16545 \cdot {C_{10}}^2 \cdot C_{01} \cdot C_{11} - 11.94747 \cdot {C_{01}}^2 \cdot C_{11} + 8.75069 \\
& \cdot C_{10} \cdot {C_{01}}^2 \cdot C_{11} - 8.82394 \cdot {C_{01}}^3 \cdot C_{11} - 235.18325 \cdot {C_{11}}^2 - 24.86589 \\
& \cdot C_{10} \cdot {C_{11}}^2 - 61.00259 \cdot C_{01} \cdot {C_{11}}^2 + 33.89034 \cdot C_{10} \cdot C_{01} \cdot {C_{11}}^2 \\
& + 18.62427 \cdot {C_{01}}^2 \cdot {C_{11}}^2 + 451.50186 \cdot {C_{11}}^3 - 314.84447 \cdot {C_{11}}^4
\end{aligned}
$$

$$EF_{trac} = 10.7161 - 38.18766 \cdot C_{10} + 59.85163 \cdot {C_{10}}^2 - 18.26953 \cdot {C_{10}}^3 - 22.45239 \cdot C_{01} + 74.93665 \cdot C_{10} \cdot C_{01} - 49.18618 \cdot {C_{10}}^2 \cdot C_{01} + 10.25661 \cdot {C_{10}}^3 \cdot C_{01} + 13.19616 \cdot {C_{01}}^2 - 20.98564 \cdot C_{10} \cdot {C_{01}}^2 + 7.3664 \cdot {C_{10}}^2 \cdot {C_{01}}^2 - 65.34749 \cdot C_{11} + 264.52875 \cdot C_{10} \cdot C_{11} - 148.04887 \cdot {C_{10}}^2 \cdot C_{11} + 25.48434 \cdot {C_{10}}^3 \cdot C_{11} + 149.48438 \cdot C_{01} \cdot C_{11} - 172.99774 \cdot C_{10} \cdot C_{01} \cdot C_{11} + 49.97172 \cdot {C_{10}}^2 \cdot C_{01} \cdot C_{11} - 20.98999 \cdot {C_{01}}^2 \cdot C_{11} + 19.88664 \cdot C_{10} \cdot {C_{01}}^2 \cdot C_{11} + 267.14785 \cdot {C_{11}}^2 - 385.7986 \cdot C_{10} \cdot {C_{11}}^2 + 102.37209 \cdot {C_{10}}^2 \cdot {C_{11}}^2 - 223.90305 \cdot C_{01} \cdot {C_{11}}^2 + 116.73425 \cdot C_{10} \cdot C_{01} \cdot {C_{11}}^2 - 195.32117 \cdot {C_{11}}^3 + 191.30201 \cdot C_{10} \cdot {C_{11}}^3 + 124.47905 \cdot C_{01} \cdot {C_{11}}^3$$

$$EF_{tens} = 702.044 - 2544.31158 \cdot C_{10} + 3304.55875 \cdot {C_{10}}^2 - 1327.07049 \cdot {C_{10}}^3 + 186.42887 \cdot {C_{10}}^4 - 2409.58568 \cdot C_{01} + 6417.23337 \cdot C_{10} \cdot C_{01} - 4024.92722 \cdot {C_{10}}^2 \cdot C_{01} + 799.34989 \cdot {C_{10}}^3 \cdot C_{01} + 2973.3778 \cdot {C_{01}}^2 - 3767.64131 \cdot C_{10} \cdot {C_{01}}^2 + 1144.90762 \cdot {C_{10}}^2 \cdot {C_{01}}^2 - 1160.06987 \cdot {C_{01}}^3 + 708.41455 \cdot C_{10} \cdot {C_{01}}^3 + 166.62487 \cdot {C_{01}}^4 - 1873.72258 \cdot C_{11} + 5446.58523 \cdot C_{10} \cdot C_{11} - 3295.48509 \cdot {C_{10}}^2 \cdot C_{11} + 583.87953 \cdot {C_{10}}^3 \cdot C_{11} + 4936.99716 \cdot C_{01} \cdot C_{11} - 6444.54512 \cdot C_{10} \cdot C_{01} \cdot C_{11} + 1977.7766 \cdot {C_{10}}^2 \cdot C_{01} \cdot C_{11} - 2593.77736 \cdot {C_{01}}^2 \cdot C_{11} + 1672.55113 \cdot C_{10} \cdot {C_{01}}^2 \cdot C_{11} + 379.73004 \cdot {C_{01}}^3 \cdot C_{11} + 2093.93193 \cdot {C_{11}}^2 - 3007.62806 \cdot C_{10} \cdot {C_{11}}^2 + 974.08201 \cdot {C_{10}}^2 \cdot {C_{11}}^2 - 2596.41312 \cdot C_{01} \cdot {C_{11}}^2 + 1935.16706 \cdot C_{10} \cdot C_{01} \cdot {C_{11}}^2 + 563.60358 \cdot {C_{01}}^2 \cdot {C_{11}}^2$$

S5.11 Mooney-Rivlin hyper-elastic model for PUR.

$$EF_{shear} = 1208.56785 - 2358.43336 \cdot C_{10} + 1635.10817 \cdot {C_{10}}^2 - 676.81596 \cdot {C_{10}}^3 + 314.08539 \cdot {C_{10}}^4 - 2276.18241 \cdot C_{01} + 2875.10359 \cdot C_{10} \cdot C_{01} - 304.12894 \cdot {C_{10}}^3 \cdot C_{01} + 1665.21675 \cdot {C_{01}}^2 - 441.46439 \cdot {C_{10}}^2 \cdot {C_{01}}^2 - 1069.51707 \cdot {C_{01}}^3 - 253.59778 \cdot C_{10} \cdot {C_{01}}^3 + 599.00937 \cdot {C_{01}}^4 - 2577.35671 \cdot C_{11} + 4354.26408 \cdot C_{10} \cdot C_{11} - 1367.01744 \cdot {C_{10}}^2 \cdot C_{11} + 4472.63173 \cdot C_{01} \cdot C_{11} - 4575.43531 \cdot C_{10} \cdot C_{01} \cdot C_{11} + 1140.19828 \cdot {C_{10}}^2 \cdot C_{01} \cdot C_{11} - 2271.6789 \cdot {C_{01}}^2 \cdot C_{11} + 1207.39338 \cdot C_{10} \cdot {C_{01}}^2 \cdot C_{11} + 807.622 \cdot {C_{01}}^3 \cdot C_{11} + 1914.79821 \cdot {C_{11}}^2 - 927.45139 \cdot {C_{10}}^2 \cdot {C_{11}}^2 - 1354.59148 \cdot {C_{01}}^2 \cdot {C_{11}}^2$$

$$EF_{comVol} = -1635.4183 + 14742.42083 \cdot C_{10} + 3400.57868 \cdot {C_{10}}^2 - 2762.10398 \cdot {C_{10}}^3 + 765.63003 \cdot {C_{10}}^4 + 15745.10924 \cdot C_{01} + 3034.48267 \cdot C_{10} \cdot C_{01} - 3719.00015 \cdot {C_{10}}^2 \cdot C_{01} + 1405.62769 \cdot {C_{10}}^3 \cdot C_{01} + 733.17345 \cdot {C_{01}}^2 - 1217.5158 \cdot C_{10} \cdot {C_{01}}^2 + 686.77408 \cdot {C_{10}}^2 \cdot {C_{01}}^2 - 1119.41507 \cdot {C_{01}}^3 + 763.81804 \cdot {C_{01}}^4$$

$$EF_{comp} = 1121.17055 - 3849.54392 \cdot C_{10} + 4643.0158 \cdot {C_{10}}^{2} - 1817.95654 \cdot {C_{10}}^{3} + 263.56871 \cdot {C_{10}}^{4} - 4826.17938 \cdot C_{01} + 11945.94396 \cdot C_{10} \cdot C_{01} - 7077.95078 \cdot {C_{10}}^{2} \cdot C_{01} + 1274.30966 \cdot {C_{10}}^{3} \cdot C_{01} + 7247.11944 \cdot {C_{01}}^{2} - 9000.94792 \cdot C_{10} \cdot {C_{01}}^{2} + 2666.41478 \cdot {C_{10}}^{2} \cdot {C_{01}}^{2} - 3460.55385 \cdot {C_{01}}^{3} + 2113.84232 \cdot C_{10} \cdot {C_{01}}^{3} + 557.68092 \cdot {C_{01}}^{4} - 2062.00744 \cdot C_{11} + 4922.97392 \cdot C_{10} \cdot C_{11} - 2500.83808 \cdot {C_{10}}^{2} \cdot C_{11} + 393.52228 \cdot {C_{10}}^{3} \cdot C_{11} + 6447.0182 \cdot C_{01} \cdot C_{11} - 7624.06867 \cdot C_{10} \cdot C_{01} \cdot C_{11} + 2037.43226 \cdot {C_{10}}^{2} \cdot C_{01} \cdot C_{11} - 4226.83673 \cdot {C_{01}}^{2} \cdot C_{11} + 2743.92478 \cdot C_{10} \cdot {C_{01}}^{2} \cdot C_{11} + 849.54197 \cdot {C_{01}}^{3} \cdot C_{11} + 1450.3687 \cdot {C_{11}}^{2} - 1151.90474 \cdot C_{10} \cdot {C_{11}}^{2} - 1559.48067 \cdot C_{01} \cdot {C_{11}}^{2} + 1050.34018 \cdot C_{10} \cdot C_{01} \cdot {C_{11}}^{2}$$

$$EF_{trac} = 40.48927 - 70.25492 \cdot C_{10} + 78.80384 \cdot {C_{10}}^{3} - 31.1337 \cdot {C_{10}}^{4} - 21.23926 \cdot C_{01} + 51.18467 \cdot {C_{10}}^{2} \cdot C_{01} - 21.10467 \cdot {C_{10}}^{3} \cdot C_{01} + 28.81611 \cdot C_{10} \cdot {C_{01}}^{2} - 23.23655 \cdot {C_{10}}^{2} \cdot {C_{01}}^{2} - 413.06956 \cdot C_{11} + 1119.06742 \cdot C_{10} \cdot C_{11} - 350.86279 \cdot {C_{10}}^{2} \cdot C_{11} + 342.83133 \cdot C_{01} \cdot C_{11} - 254.57283 \cdot C_{10} \cdot C_{01} \cdot C_{11} + 38.96202 \cdot {C_{10}}^{2} \cdot C_{01} \cdot C_{11} - 40.1304 \cdot {C_{01}}^{2} \cdot C_{11} + 28.53367 \cdot C_{10} \cdot {C_{01}}^{2} \cdot C_{11} + 3036.21102 \cdot {C_{11}}^{2} - 2645.75059 \cdot C_{10} \cdot {C_{11}}^{2} + 442.12347 \cdot {C_{10}}^{2} \cdot {C_{11}}^{2} - 637.70026 \cdot C_{01} \cdot {C_{11}}^{2} + 208.43968 \cdot C_{10} \cdot C_{01} \cdot {C_{11}}^{2} - 4802.05427 \cdot {C_{11}}^{3} + 2087.10966 \cdot C_{10} \cdot {C_{11}}^{3} + 470.4058 \cdot C_{01} \cdot {C_{11}}^{3} + 2640.16143 \cdot {C_{11}}^{4}$$

$$EF_{tens} = 1072.40184 - 1572.19641 \cdot {C_{10}}^{2} + 1065.74331 \cdot {C_{10}}^{3} - 198.00613 \cdot {C_{10}}^{4} - 3233.77545 \cdot C_{10} \cdot C_{01} + 3227.42206 \cdot {C_{10}}^{2} \cdot C_{01} - 703.05089 \cdot {C_{10}}^{3} \cdot C_{01} - 1400.7919 \cdot {C_{01}}^{2} + 3068.38116 \cdot C_{10} \cdot {C_{01}}^{2} - 1049.83467 \cdot {C_{10}}^{2} \cdot {C_{01}}^{2} + 903.53428 \cdot {C_{01}}^{3} - 689.34375 \cdot C_{10} \cdot {C_{01}}^{3} - 152.18398 \cdot {C_{01}}^{4} - 379.1367 \cdot C_{11} - 1914.17042 \cdot C_{10} \cdot C_{11} + 2451.43561 \cdot {C_{10}}^{2} \cdot C_{11} - 560.60634 \cdot {C_{10}}^{3} \cdot C_{11} - 1559.48796 \cdot C_{01} \cdot C_{11} + 4926.4631 \cdot C_{10} \cdot C_{01} \cdot C_{11} - 2023.50996 \cdot {C_{10}}^{2} \cdot C_{01} \cdot C_{11} + 1662.69914 \cdot {C_{01}}^{2} \cdot C_{11} - 1701.87461 \cdot C_{10} \cdot {C_{01}}^{2} \cdot C_{11} - 341.45735 \cdot {C_{01}}^{3} \cdot C_{11} + 1146.07752 \cdot C_{10} \cdot {C_{11}}^{2} - 656.19947 \cdot {C_{10}}^{2} \cdot {C_{11}}^{2} + 694.24232 \cdot C_{01} \cdot {C_{11}}^{2} - 1041.67299 \cdot C_{10} \cdot C_{01} \cdot {C_{11}}^{2}$$

S5.12 Mooney-Rivlin hyper-elastic model for SBR.

$$EF_{shear} = 369.23988 - 1239.47992 \cdot C_{10} + 1900.3982 \cdot {C_{10}}^{2} - 576.89576 \cdot {C_{10}}^{3} - 1231.95871 \cdot C_{01} + 4044.94005 \cdot C_{10} \cdot C_{01} - 2689.86999 \cdot {C_{10}}^{2} \cdot C_{01} + 595.3994 \cdot {C_{10}}^{3} \cdot C_{01} + 1656.7623 \cdot {C_{01}}^{2} - 2393.14325 \cdot C_{10} \cdot {C_{01}}^{2} + 766.62795 \cdot {C_{10}}^{2} \cdot {C_{01}}^{2} + 433.67074 \cdot C_{10} \cdot {C_{01}}^{3} - 370.03922 \cdot {C_{01}}^{4} - 631.02847 \cdot C_{11} + 2658.51259 \cdot C_{10} \cdot C_{11} - 1235.27385 \cdot {C_{10}}^{2} \cdot C_{11} + 2549.84281 \cdot C_{01} \cdot C_{11} - 3003.08718 \cdot C_{10} \cdot C_{01} \cdot C_{11} + 824.04635 \cdot {C_{10}}^{2} \cdot C_{01} \cdot C_{11} - 1173.8078 \cdot {C_{01}}^{2} \cdot C_{11} + 616.97518 \cdot C_{10} \cdot {C_{01}}^{2} \cdot C_{11} + 999.23686 \cdot {C_{11}}^{2} - 2276.86829 \cdot C_{10} \cdot {C_{11}}^{2} + 1041.23121 \cdot {C_{10}}^{2} \cdot {C_{11}}^{2} - 2258.3776 \cdot C_{01} \cdot {C_{11}}^{2} + 1432.59858 \cdot C_{10} \cdot C_{01} \cdot {C_{11}}^{2} + 1134.06838 \cdot {C_{01}}^{2} \cdot {C_{11}}^{2}$$

$$EF_{comVol} = -1664.28672 + 13389.87436 \cdot C_{10} + 6233.52066 \cdot {C_{10}}^{2} - 5063.14069 \cdot {C_{10}}^{3} + 1403.45434 \cdot {C_{10}}^{4} + 15227.85926 \cdot C_{01} + 5562.46787 \cdot C_{10} \cdot C_{01} - 6817.23689 \cdot {C_{10}}^{2} \cdot C_{01} + 2576.62838 \cdot {C_{10}}^{3} \cdot C_{01} + 1343.98254 \cdot {C_{01}}^{2} - 2231.82042 \cdot C_{10} \cdot {C_{01}}^{2} + 1258.92303 \cdot {C_{10}}^{2} \cdot {C_{01}}^{2} - 2052.00598 \cdot {C_{01}}^{3} + 1400.15555 \cdot {C_{01}}^{4}$$

$$EF_{comp} = -41.84491 + 618.05442 \cdot C_{10} + 358.88382 \cdot {C_{10}}^{2} - 68.63083 \cdot {C_{10}}^{3} + 655.08806 \cdot C_{01} + 1189.7664 \cdot C_{10} \cdot C_{01} - 566.94943 \cdot {C_{10}}^{2} \cdot C_{01} + 75.1354 \cdot {C_{10}}^{3} \cdot C_{01} + 840.34603 \cdot {C_{01}}^{2} - 823.685 \cdot C_{10} \cdot {C_{01}}^{2} + 201.54243 \cdot {C_{10}}^{2} \cdot {C_{01}}^{2} - 303.47064 \cdot {C_{01}}^{3} + 174.29645 \cdot C_{10} \cdot {C_{01}}^{3} - 627.5097 \cdot C_{11} + 2164.3238 \cdot C_{10} \cdot C_{11} - 748.40826 \cdot {C_{10}}^{2} \cdot C_{11} + 3177.94743 \cdot C_{01} \cdot C_{11} - 2643.51063 \cdot C_{10} \cdot C_{01} \cdot C_{11} + 534.89504 \cdot {C_{10}}^{2} \cdot C_{01} \cdot C_{11} - 1965.44476 \cdot {C_{01}}^{2} \cdot C_{11} + 784.36061 \cdot C_{10} \cdot {C_{01}}^{2} \cdot C_{11} + 374.67661 \cdot {C_{01}}^{3} \cdot C_{11} + 3174.11625 \cdot {C_{11}}^{2} - 3002.56543 \cdot C_{10} \cdot {C_{11}}^{2} + 549.32786 \cdot {C_{10}}^{2} \cdot {C_{11}}^{2} - 4382.7177 \cdot C_{01} \cdot {C_{11}}^{2} + 1642.45221 \cdot C_{10} \cdot C_{01} \cdot {C_{11}}^{2} + 1235.40478 \cdot {C_{01}}^{2} \cdot {C_{11}}^{2} - 2295.59015 \cdot {C_{11}}^{3} + 1379.58005 \cdot C_{10} \cdot {C_{11}}^{3} + 2009.55928 \cdot C_{01} \cdot {C_{11}}^{3}$$

$$EF_{trac} = 0.37636 + 23.65289 \cdot C_{10} + 21.79452 \cdot {C_{10}}^{2} - 2.38894 \cdot {C_{10}}^{4} - 12.34415 \cdot C_{01} + 45.66054 \cdot C_{10} \cdot C_{01} - 16.06698 \cdot {C_{10}}^{2} \cdot C_{01} + 12.70574 \cdot {C_{01}}^{2} - 12.25116 \cdot C_{10} \cdot {C_{01}}^{2} + 306.12097 \cdot C_{11} + 245.21048 \cdot C_{10} \cdot C_{11} - 113.32477 \cdot {C_{10}}^{2} \cdot C_{11} + 18.64686 \cdot {C_{10}}^{3} \cdot C_{11} + 222.06786 \cdot C_{01} \cdot C_{11} - 216.96653 \cdot C_{10} \cdot C_{01} \cdot C_{11} + 61.60931 \cdot {C_{10}}^{2} \cdot C_{01} \cdot C_{11} - 52.95916 \cdot {C_{01}}^{2} \cdot C_{11} + 48.38727 \cdot C_{10} \cdot {C_{01}}^{2} \cdot C_{11} + 162.41135 \cdot {C_{11}}^{2} - 358.33089 \cdot C_{10} \cdot {C_{11}}^{2} + 52.0841 \cdot {C_{10}}^{2} \cdot {C_{11}}^{2} - 358.67911 \cdot C_{01} \cdot {C_{11}}^{2} + 102.56671 \cdot C_{10} \cdot C_{01} \cdot {C_{11}}^{2} + 256.41066 \cdot C_{10} \cdot {C_{11}}^{3} + 298.01128 \cdot C_{01} \cdot {C_{11}}^{3} - 201.4063 \cdot {C_{11}}^{4}$$

$$EF_{tens} = 678.99302 - 2201.94287 \cdot C_{10} + 2714.32629 \cdot {C_{10}}^{2} - 995.29534 \cdot {C_{10}}^{3} + 124.75272 \cdot {C_{10}}^{4} - 2054.46431 \cdot C_{01} + 5163.21263 \cdot C_{10} \cdot C_{01} - 2975.23671 \cdot {C_{10}}^{2} \cdot C_{01} + 548.11557 \cdot {C_{10}}^{3} \cdot C_{01} + 2276.08348 \cdot {C_{01}}^{2} - 2667.91349 \cdot C_{10} \cdot {C_{01}}^{2} + 745.33481 \cdot {C_{10}}^{2} \cdot {C_{01}}^{2} - 628.30578 \cdot {C_{01}}^{3} + 420.77853 \cdot C_{10} \cdot {C_{01}}^{3} - 1704.54052 \cdot C_{11} + 5146.82451 \cdot C_{10} \cdot C_{11} - 3114.62815 \cdot {C_{10}}^{2} \cdot C_{11} + 545.30936 \cdot {C_{10}}^{3} \cdot C_{11} + 4560.05716 \cdot C_{01} \cdot C_{11} - 5961.08725 \cdot C_{10} \cdot C_{01} \cdot C_{11} + 1811.49183 \cdot {C_{10}}^{2} \cdot C_{01} \cdot C_{11} - 2336.74279 \cdot {C_{01}}^{2} \cdot C_{11} + 1530.42535 \cdot C_{10} \cdot {C_{01}}^{2} \cdot C_{11} + 315.74613 \cdot {C_{01}}^{3} \cdot C_{11} + 1977.03494 \cdot {C_{11}}^{2} - 2966.17265 \cdot C_{10} \cdot {C_{11}}^{2} + 1007.68127 \cdot {C_{10}}^{2} \cdot {C_{11}}^{2} - 2442.26545 \cdot C_{01} \cdot {C_{11}}^{2} + 1873.6857 \cdot C_{10} \cdot C_{01} \cdot {C_{11}}^{2} + 517.87698 \cdot {C_{01}}^{2} \cdot {C_{11}}^{2}$$

S5.13 Arruda-Boyce hyper-elastic model for NBR.

$$EF_{shear} = 343.87777 - 870.83475 \cdot Nkt + 1037.17292 \cdot Nkt^{2} - 25.42125 \cdot Nkt \cdot \lambda + 0.39898 \cdot \lambda^{2}$$

$$EF_{comVol} = -1919.42829 + 7508.96782 \cdot Nkt + 456.54146 \cdot Nkt^{2} - 0.08931 \cdot Nkt \cdot \lambda + 0.00112 \cdot \lambda^{2}$$

$$EF_{comp} = 371.23109 - 1126.44011 \cdot Nkt + 1432.93383 \cdot Nkt^{2} - 29.70991 \cdot Nkt \cdot \lambda + 0.43446 \cdot \lambda^{2}$$

$$EF_{trac} = 197.47187 + 1051.58888 \cdot Nkt - 61.52851 \cdot \lambda - 50.61817 \cdot Nkt \cdot \lambda + 2.36001 \cdot \lambda^{2}$$

$$EF_{tens} = 532.62137 - 851.88933 \cdot Nkt + 357.76776 \cdot Nkt^{2} + 13.91383 \cdot \lambda - 0.34594 \cdot \lambda^{2}$$

S5.14 Arruda-Boyce hyper-elastic model for EVA.

$$EF_{shear} = 443.03704 - 815.96357 \cdot Nkt + 573.86517 \cdot Nkt^{2} + 10.01283 \cdot \lambda - 15.063 \cdot Nkt \cdot \lambda$$

$$EF_{comVol} = -1151.42377 + 4636.76594 \cdot Nkt + 175.84276 \cdot Nkt^{2} - 0.02159 \cdot \lambda - 0.04255 \cdot Nkt \cdot \lambda + 0.00117 \cdot \lambda^{2}$$

$$EF_{comp} = -32.04627 + 940.69416 \cdot Nkt - 20.19724 \cdot \lambda - 16.12471 \cdot Nkt \cdot \lambda + 0.74803 \cdot \lambda^{2}$$

$$EF_{trac} = 4.91399 + 25.53328 \cdot Nkt^{2} - 1.95244 \cdot Nkt \cdot \lambda + 0.02786 \cdot \lambda^{2}$$

$$EF_{tens} = 496.09381 - 875.14162 \cdot Nkt + 517.78397 \cdot Nkt^2 + 7.92435 \cdot \lambda - 8.23417 \cdot Nkt \cdot \lambda$$

S5.15 Arruda-Boyce hyper-elastic model for PUR.

$$EF_{shear} = 884.10358 - 807.84821 \cdot Nkt + 18.95852 \cdot \lambda + 14.42898 \cdot Nkt \cdot \lambda - 0.6924 \cdot \lambda^2$$

$$EF_{comVol} = -1853.06435 + 8184.12015 \cdot Nkt - 0.06229 \cdot \lambda - 0.05012 \cdot Nkt \cdot \lambda + 0.00231 \cdot \lambda^2$$

$$EF_{comp} = 714.18438 - 1009.39545 \cdot Nkt + 276.74001 \cdot Nkt^2 + 18.541 \cdot \lambda + 4.47422 \cdot Nkt \cdot \lambda - 0.52967 \cdot \lambda^2$$

$$EF_{trac} = 192.03576 + 1036.39488 \cdot Nkt - 59.06482 \cdot \lambda - 50.614 \cdot Nkt \cdot \lambda + 2.3021 \cdot \lambda^2$$

$$EF_{tens} = 1104.64969 - 620.13626 \cdot Nkt + 13.57893 \cdot \lambda + 10.82928 \cdot Nkt \cdot \lambda - 0.50292 \cdot \lambda^2$$

S5.16 Arruda-Boyce hyper-elastic model for SBR.

$$EF_{shear} = 345.66083 - 758.6238 \cdot Nkt + 721.73296 \cdot Nkt^2 + 8.8817 \cdot \lambda - 17.40945 \cdot Nkt \cdot \lambda$$

$$EF_{comVol} = -2063.42994 + 8184.12015 \cdot Nkt - 0.06229 \cdot \lambda - 0.05012 \cdot Nkt \cdot \lambda + 0.00231 \cdot \lambda^2$$

$$EF_{comp} = 44.81323 + 709.16551 \cdot Nkt^2 - 31.25226 \cdot Nkt \cdot \lambda + 0.42723 \cdot \lambda^2$$

$$EF_{trac} = 210.08771 + 1056.59961 \cdot Nkt - 63.91641 \cdot \lambda - 48.90095 \cdot Nkt \cdot \lambda + 2.38979 \cdot \lambda^2$$

$$EF_{tens} = 510.44981 - 819.16068 \cdot Nkt + 482.55608 \cdot Nkt^2 + 7.95319 \cdot \lambda - 8.94834 \cdot Nkt \cdot \lambda$$

S6. Correlation (Corr), p-value, MAE and RMSE obtained for each of the polynomial models

Table S21. ANOVA values of *"EFs"* second-order polynomial models for the NBR material.

Model	Var.	Corr	*p*-value	train.MAE	train.RMSE	test.MAE	test.RMSE
**Mooney-Rivlin**	*EF_shear*	0.995	5.883E-72	0.0119	0.0242	0.0091	0.0115
	EF_comVol	0.999	4.307E-182	0.0016	0.0021	0.0014	0.0017
	EF _comp	0.999	8.020E-118	0.0035	0.0055	0.0026	0.0028
	EF _trac	0.999	1.190E-108	0.0051	0.0073	0.0069	0.0112
	EF _tens	0.999	2.622E-93	0.0067	0.0092	0.0079	0.0152
**Arruda-boyce**	*EF _shear*	0.931	0.04746	0.095	0.110	0.117	0.154
	EF_comVol	0.999	3.646E-17	2.138E-05	2.410E-05	0.0043	0.0049
	EF _comp	0.952	0.0241	0.071	0.0843	0.113	0.135
	EF _trac	0.978	0.00518	0.055	0.0702	0.105	0.121
	EF _tens	0.980	0.00431	0.063	0.0731	0.0339	0.0419
**Gent**	*EF _shear*	0.999	3.560E-51	8.130E-05	0.0001	0.0341	0.0356
	EF_comVol	1	2.174E-118	1.426E-10	2.047E-10	0.0031	0.00312
	EF _comp	0.999	1.015E-55	4.020E-05	5.292E-05	0.0535	0.0539
	EF _trac	0.999	4.451E-31	0.0012	0.0016	0.0211	0.0240
	EF _tens	1	2.057E-108	3.479E-08	4.773E-08	2.079E-08	2.527E-08
**Ogden**	*EF _shear*	0.997	2.879E-06	0.0142	0.0213	0.0286	0.0353
	EF _comVol	0.999	0.00075	0.01031	0.0124	0.0103	0.0144
	EF _comp	0.993	4.963E-05	0.0250	0.0329	0.0384	0.0442
	EF _trac	0.999	1.40198E-09	0.0052	0.0069	0.0416	0.0455
	EF _tens	0.999	3.27453E-08	0.0015	0.0019	0.0034	0.0051

Table S22. ANOVA values of the *"EFs"* second-order polynomial models for the PUR material.

Model	Var.	Corr	*p*-value	train.MAE	train.RMSE	test.MAE	test.RMSE
**Monney-Rivlin**	*EF _shear*	0.989	1.655E-55	0.0179	0.0325	0.0132	0.0153
	EF_comVol	0.999	2.496E-213	0.00071	0.00094	0.00069	0.0008
	EF _comp	0.999	1.906E-98	0.00660	0.00851	0.0081	0.0088
	EF _trac	0.999	7.564E-103	0.00702	0.0092	0.0106	0.0173
	EF _tens	0.998	2.826E-83	0.0120	0.0149	0.0121	0.0136
**Arruda-boyce**	*EF _shear*	0.995	0.000295	0.0239	0.03031	0.0426	0.0457
	EF_comVol	0.999	2.529E-18	9.521E-06	1.236E-05	1.638E-05	1.755E-05
	EF _comp	0.994	0.00397	0.0306	0.03552	0.0217	0.0352
	EF _trac	0.976	0.00621	0.0566	0.07305	0.1098	0.1251
	EF _tens	0.995	0.000276	0.0230	0.02990	0.0408	0.0438
**Gent**	*EF _shear*	0.999	1.424E-66	3.647E-08	4.974E-08	2.608E-08	3.123E-08
	EF_comVol	0.999	1.080E-118	1.323E-10	1.900E-10	0.00129	0.00129
	EF _comp	0.999	5.124E-66	1.750E-07	2.339E-07	0.00010	0.00011
	EF _trac	0.999	5.722E-28	0.00392	0.00527	0.01562	0.0184
	EF _tens	0.999	1.692E-110	2.582E-08	3.537E-08	1.5341E-08	1.871E-08
**Ogden**	*EF _shear*	0.999	1.448E-11	2.628E-05	3.084E-05	0.00010	0.00016
	EF _comVol	0.999	0.00027	0.00760	0.0090	0.00697	0.0098
	EF _comp	0.999	9.759E-08	0.00196	0.00249	0.00452	0.0068
	EF _trac	0.999	4.859E-10	0.00423	0.00570	0.01847	0.0248
	EF _tens	0.999	9.832E-16	1.866E-05	2.479E-05	6.927E-05	0.00010

285 **Table S23.** ANOVA values of the *"EFs"* second-order polynomial models for the EVA material.

Model	Var.	Corr	*p*-value	train.MAE	train.RMSE	test.MAE	test.RMSE
Monney-Rivlin	EF _shear	0.99	6.743E-90	0.00712	0.01134	0.00548	0.00638
	EF _comVol	0.999	1.504E-188	0.00136	0.00180	0.00122	0.00151
	EF _comp	0.999	1.632E-242	4.566E-05	6.382E-05	3.175E-05	3.791E-05
	EF _trac	0.999	2.041E-97	0.00601	0.00872	0.0071	0.01018
	EF _tens	0.999	3.197E-101	0.00536	0.00682	0.0060	0.00885
Arruda-boyce	EF _shear	0.918	0.065922	0.128	0.145	0.0700	0.0857
	EF _comVol	0.999	3.520E-13	1.679E-05	1.917E-05	0.0027	0.0031
	EF _comp	0.995	0.000256	0.0226	0.029	0.0400	0.0429
	EF _trac	0.923	0.016074	0.094	0.1128	0.0856	0.1029
	EF _tens	0.939	0.038465	0.108	0.125	0.0726	0.0839
Gent	EF _shear	0.999	6.736E-56	7.231E-05	9.531E-05	0.0055	0.0055
	EF _comVol	1	1.806E-115	5.851E-12	7.676E-12	0.0025	0.0025
	EF _comp	1	1.610E-78	1.732E-07	2.307E-07	4.094E-07	5.983E-07
	EF _trac	0.999	3.028E-33	0.0018	0.0024	0.0627	0.0643
	EF _tens	0.999	6.946E-49	6.629E-05	8.829E-05	0.0053	0.0055
Ogden	EF _shear	0.998	2.748E-11	0.0118	0.0147	0.0155	0.01807
	EF _comVol	0.999	0.000654	0.0090	0.011	0.0094	0.0112
	EF _comp	0.999	1.924E-08	0.00121	0.0015	0.0017	0.0037
	EF _trac	0.998	1.114E-06	0.0125	0.017	0.0209	0.0250
	EF _tens	0.999	0.00012	0.00520	0.0065	0.0076	0.0116

286
287

Table S24. ANOVA values of the *"EFs"* second-order polynomial models for the SBR material.

Model	Var.	Corr	*p*-value	train.MAE	train.RMSE	test.MAE	test.RMSE
**Monney-Rivlin**	*EF _shear*	0.998	2.2706E-82	0.00801	0.0152	0.00737	0.0089
	EF_comVol	0.999	8.218E-190	0.00132	0.00175	0.00117	0.00146
	EF _comp	0.999	2.258E-121	0.00218	0.00360	0.00154	0.00182
	EF _trac	0.999	2.542E-143	0.00183	0.00266	0.0017	0.0023
	EF _tens	0.999	1.943E-105	0.00480	0.00617	0.0048	0.0065
**Arruda-boyce**	*EF _shear*	0.919	0.0640	0.1311	0.15819	0.0972	0.1257
	EF_comVol	1	2.529E-18	9.521E-06	1.236E-05	1.638E-05	1.755E-05
	EF _comp	0.956	0.00411	0.0767	0.0871	0.0577	0.0717
	EF _trac	0.980	0.00424	0.0522	0.0669	0.1051	0.1197
	EF _tens	0.943	0.0333	0.1079	0.1237	0.0670	0.0809
**Gent**	*EF _shear*	0.999	1.0974E-55	4.190E-05	5.466E-05	0.00918	0.01058
	EF_comVol	1	1.733E-118	1.391E-10	1.997E-10	0.00248	0.00249
	EF _comp	0.999	7.114E-51	8.067E-05	0.00010	0.01226	0.01327
	EF _trac	0.999	8.838E-33	0.00032	0.00043	0.00710	0.00725
	EF _tens	0.999	1.291E-48	7.112E-05	9.473E-05	0.00813	0.00847
**Ogden**	*EF _shear*	0.998	4.705E-07	0.00978	0.01552	0.01963	0.02516
	EF _comVol	0.999	0.000569	0.00951	0.01139	0.00927	0.01281
	EF _comp	0.995	1.538E-05	0.02249	0.02752	0.01770	0.02218
	EF _trac	0.999	7.265E-09	0.00115	0.00154	0.01208	0.01727
	EF _tens	0.999	1.304E-07	0.00220	0.0029	0.00711	0.00921

S7. Multi-Response Optimization

Tables S25, S26, S27 and S28 show the combination of C_i constants that are most appropriate for modeling the mechanical behaviour of NBR, EVA, SBR and PUR materials according to hyper-elastic models (Mooney-Rivlin, Arruda-Boyce, Gent and Ogden) by desirability functions. The first column in all tables shows hyper-elastic material models that were proposed. The second column shows the C_i constants and the EF_{norm} for each standardized test. The third column shows the goal that was established in the optimization process for both C_i constants and EF_{norm}. The fourth and fifth columns show, respectively, the minimum and maximum values of C_i constants and EF_{norm} (range). The sixth column shows the degree of importance considered in the optimization process, whereas the seventh and eighth columns show, respectively, the optimal values obtained for the C_i constants and the EF_{norm}, and the desirability values.

Table S25. Adjustment of the Ci constants of the Hyper-elastic models for the NBR material.

Model	C_i and $EF_{Norm,k}$	Goal	min	max	Importance	Value	Desirability
Mooney-rivlin	C10	In range	-0.25	1.25	1.0	0.367	1
	C01	In range	-0.3	1	1.0	-0.069	1
	C11	In range	0	0.5	1.0	0.005	1
	$EF_{Norm,Shear}$	Min.	96.174	2241.868	1.0	96.07	1
	$EF_{Norm,ComVol}$	Min.	297.456	34666.017	1.0	2627.096	0.932
	$EF_{Norm,Comp}$	Min.	62.996	2971.651	1.0	173.942	0.962
	$EF_{Norm,Trac}$	Min.	1.753	280.073	1.0	11.888	0.964
	$EF_{Norm,Tens}$	Min.	63.309	1751.024	1.0	232.418	0.9
					Overall Desirability		0.951
Arruda-boyce	Nkt	In range	0.26	0.9	1.0	0.578	1
	Chain	In range	2	25	1.0	24.644	1
	$EF_{Norm,Shear}$	Min.	70.357	392.841	1.0	67.291	1
	$EF_{Norm,ComVol}$	Min.	63.665	5208.335	1.0	2571.347	0.513
	$EF_{Norm,Comp}$	Min.	32.527	492.684	1.0	39.565	0.985
	$EF_{Norm,Trac}$	Min.	3.574	1000.113	1.0	1.282	1
	$EF_{Norm,Tens}$	Min.	115.47	457.292	1.0	292.62	0.482
					Overall Desirability		0.753
Gent	E	In range	0.6	3.7	1.0	2.144	1
	inv1	In range	63	79.5	1.0	76.465	1
	$EF_{Norm,Shear}$	Min.	45.332	269.846	1.0	45.527	0.999
	$EF_{Norm,ComVol}$	Min.	520.843	7934.909	1.0	3690.353	0.573
	$EF_{Norm,Comp}$	Min.	46.923	341.163	1.0	46.641	1
	$EF_{Norm,Trac}$	Min.	2.125	21.072	1.0	2.145	0.999
	$EF_{Norm,Tens}$	Min.	35.373	485.943	1.0	261.921	0.497
					Overall Desirability		0.777
Ogden	k1	In range	0	0.5	1.0	0.254	1
	k2	In range	-0.5	-0.125	1.0	-0.261	1
	$EF_{Norm,Shear}$	Min.	46	309.481	1.0	70.573	0.907

$EF_{Norm,ComVol}$	Min.	399.743	8071.295	1.0	3139.578	0.643
$EF_{Norm,Comp}$	Min.	19.145	421.35	1.0	55.628	0.909
$EF_{Norm,Trac}$	Min.	4.182	31.899	1.0	4.644	0.983
$EF_{Norm,Tens}$	Min.	27.798	522.41	1.0	282.316	0.485
				Overall Desirability		0.759

Table S26. Adjustment of the Ci constants of the Hyper-elastic models for the PUR material.

Model	C_i and $EF_{Norm,k}$	Goal	min	max	Importance	Value	Desirability
Mooney-rivlin	C10	In range	-0.25	1.25	1.0	0.982	1
	C01	In range	-0.3	1	1.0	-0.056	1
	C11	In range	0	0.5	1.0	0.005	1
	$EF_{Norm,Shear}$	Min.	112.854	1654.109	1.0	111.625	1
	$EF_{Norm,ComVol}$	Min.	249.253	34970.176	1.0	13303.154	0.624
	$EF_{Norm,Comp}$	Min.	73.712	2566.555	1.0	276.739	0.919
	$EF_{Norm,Trac}$	Min.	1.163	266.489	1.0	18.319	0.935
	$EF_{Norm,Tens}$	Min.	152.375	1039.498	1.0	421.419	0.697
					Overall Desirability		0.821
Arruda-boyce	Nkt	In range	0.26	0.9	1.0	0.643	1
	Chain	In range	2	25	1.0	3.75	1
	$EF_{Norm,Shear}$	Min.	203.456	825.433	1.0	460.967	0.586
	$EF_{Norm,ComVol}$	Min.	274.31	5512.494	1.0	3407.264	0.402
	$EF_{Norm,Comp}$	Min.	87.588	626.636	1.0	252.565	0.694
	$EF_{Norm,Trac}$	Min.	9.289	987.47	1.0	547.094	0.45
	$EF_{Norm,Tens}$	Min.	578.934	1051.183	1.0	775.986	0.583
					Overall Desirability		0.532
Gent	E	In range	0.6	3.7	1.0	2.982	1
	inv1	In range	63	79.5	1.0	70.645	1
	$EF_{Norm,Shear}$	Min.	353.641	857.605	1.0	470.45	0.768
	$EF_{Norm,ComVol}$	Min.	249.261	8239.069	1.0	6276.983	0.246
	$EF_{Norm,Comp}$	Min.	74.689	665.016	1.0	207.921	0.774
	$EF_{Norm,Trac}$	Min.	7.661	34.656	1.0	7.662	1
	$EF_{Norm,Tens}$	Min.	684.051	1076.193	1.0	775.157	0.768
					Overall Desirability		0.645
Ogden	k1	In range	0	0.5	1.0	0.329	1
	k2	In range	-0.5	-0.125	1.0	-0.499	1
	$EF_{Norm,Shear}$	Min.	315.165	897.24	1.0	453.004	0.763
	$EF_{Norm,ComVol}$	Min.	703.902	8375.454	1.0	6235.196	0.279
	$EF_{Norm,Comp}$	Min.	33.614	691.354	1.0	137.955	0.841
	$EF_{Norm,Trac}$	Min.	9.621	41.194	1.0	9.179	1
	$EF_{Norm,Tens}$	Min.	663.501	1107.37	1.0	769.026	0.762
					Overall Desirability		0.671

Table S27. Adjustment of the Ci constants of the Hyper-elastic models for the EVA material.

Model	C_i and $EF_{Norm,k}$	Goal	min	max	Importance	Value	Desirability
Mooney-rivlin	C10	In range	-0.25	1.25	1.0	0.572	1
	C01	In range	-0.3	1	1.0	-0.292	1
	C11	In range	0	0.5	1.0	0.002	1
	$EF_{Norm,Shear}$	Min.	61.501	1893.316	1.0	153.139	0.95
	$EF_{Norm,ComVol}$	Min.	225.662	20789.563	1.0	1471.67	0.939
	$EF_{Norm,Comp}$	Min.	78.213	3245.309	1.0	137.191	0.981
	$EF_{Norm,Trac}$	Min.	1.193	69.898	1.0	3.567	0.965
	$EF_{Norm,Tens}$	Min.	71.945	1798.992	1.0	192.881	0.93
					Overall Desirability		0.952
Arruda-boyce	Nkt	In range	0.26	0.9	1.0	0.567	1
	Chain	In range	2	25	1.0	15.054	1
	$EF_{Norm,Shear}$	Min.	73.393	380.402	1.0	187.217	0.629
	$EF_{Norm,ComVol}$	Min.	65.893	3164.011	1.0	1531.532	0.527
	$EF_{Norm,Comp}$	Min.	52.119	766.343	1.0	228.846	0.753
	$EF_{Norm,Trac}$	Min.	1.087	24.657	1.0	2.771	0.929
	$EF_{Norm,Tens}$	Min.	88.508	409.325	1.0	215.548	0.604
					Overall Desirability		0.674
Gent	E	In range	0.6	3.7	1.0	2.237	1
	inv1	In range	63	79.5	1.0	63.1	1
	$EF_{Norm,Shear}$	Min.	65.828	410.551	1.0	160.167	0.726
	$EF_{Norm,ComVol}$	Min.	263.955	4795.415	1.0	2412.535	0.526
	$EF_{Norm,Comp}$	Min.	13.739	614.821	1.0	331.24	0.472
	$EF_{Norm,Trac}$	Min.	1.829	9.326	1.0	1.84	0.999
	$EF_{Norm,Tens}$	Min.	51.687	437.976	1.0	199.245	0.618
					Overall Desirability		0.644
Ogden	k1	In range	0	0.5	1.0	0.361	1
	k2	In range	-0.5	0.062	1.0	-0.119	1
	$EF_{Norm,Shear}$	Min.	77.888	447.312	1.0	181.346	0.72
	$EF_{Norm,ComVol}$	Min.	286.864	4877.019	1.0	1985.402	0.63
	$EF_{Norm,Comp}$	Min.	12.6	695.009	1.0	297.922	0.582
	$EF_{Norm,Trac}$	Min.	1.284	11.388	1.0	2.182	0.911
	$EF_{Norm,Tens}$	Min.	55.985	474.442	1.0	217.144	0.615
					Overall Desirability		0.682

Table S28. Adjustment of the Ci constants of the Hyper-elastic models for the SBR material.

Model	C_i and $EF_{Norm,k}$	Goal	min	max	Importance	Value	Desirability
Mooney-rivlin	C10	In range	-0.25	1.25	1.0	0.112	1
	C01	In range	-0.3	1	1.0	0.152	1
	C11	In range	0	0.5	1.0	0.005	1
	$EF_{Norm,Shear}$	Min.	91.287	1795.679	1.0	162.006	0.959
	$EF_{Norm,ComVol}$	Min.	391.249	34759.81	1.0	2329.807	0.944
	$EF_{Norm,Comp}$	Min.	35.444	3060.174	1.0	167.375	0.956
	$EF_{Norm,Trac}$	Min.	2.145	293.328	1.0	4.091	0.993
	$EF_{Norm,Tens}$	Min.	101.068	1774.579	1.0	275.728	0.896
					Overall Desirability		0.949
Arruda-boyce	Nkt	In range	0.26	0.9	1.0	0.579	1
	Chain	In range	2	25	1.0	17.354	1
	$EF_{Norm,Shear}$	Min.	93.858	281.635	1.0	127.498	0.821
	$EF_{Norm,ComVol}$	Min.	63.944	5302.129	1.0	2677.111	0.501
	$EF_{Norm,Comp}$	Min.	57.417	581.207	1.0	97.294	0.924
	$EF_{Norm,Trac}$	Min.	4.017	1013.368	1.0	-58.919	1
	$EF_{Norm,Tens}$	Min.	128.999	433.737	1.0	245.892	0.616
					Overall Desirability		0.747
Gent	E	In range	0.6	3.7	1.0	1.899	1
	inv1	In range	63	79.5	1.0	79.46	1
	$EF_{Norm,Shear}$	Min.	96.5	309.763	1.0	119.881	0.89
	$EF_{Norm,ComVol}$	Min.	427.05	8028.703	1.0	3091.726	0.649
	$EF_{Norm,Comp}$	Min.	54.818	429.685	1.0	91.734	0.902
	$EF_{Norm,Trac}$	Min.	3.275	33.698	1.0	8.386	0.832
	$EF_{Norm,Tens}$	Min.	102.337	462.388	1.0	275.889	0.518
					Overall Desirability		0.741
Ogden	k1	In range	0	0.5	1.0	0.124	1
	k2	In range	-0.5	-0.125	1.0	-0.426	1
	$EF_{Norm,Shear}$	Min.	94.375	343.729	1.0	126.842	0.87
	$EF_{Norm,ComVol}$	Min.	493.537	8165.088	1.0	2831.922	0.695
	$EF_{Norm,Comp}$	Min.	32.54	509.873	1.0	119.565	0.818
	$EF_{Norm,Trac}$	Min.	3.405	45.155	1.0	4.209	0.981
	$EF_{Norm,Tens}$	Min.	103.401	498.855	1.0	284.985	0.541
					Overall Desirability		0.765

S8. Validation

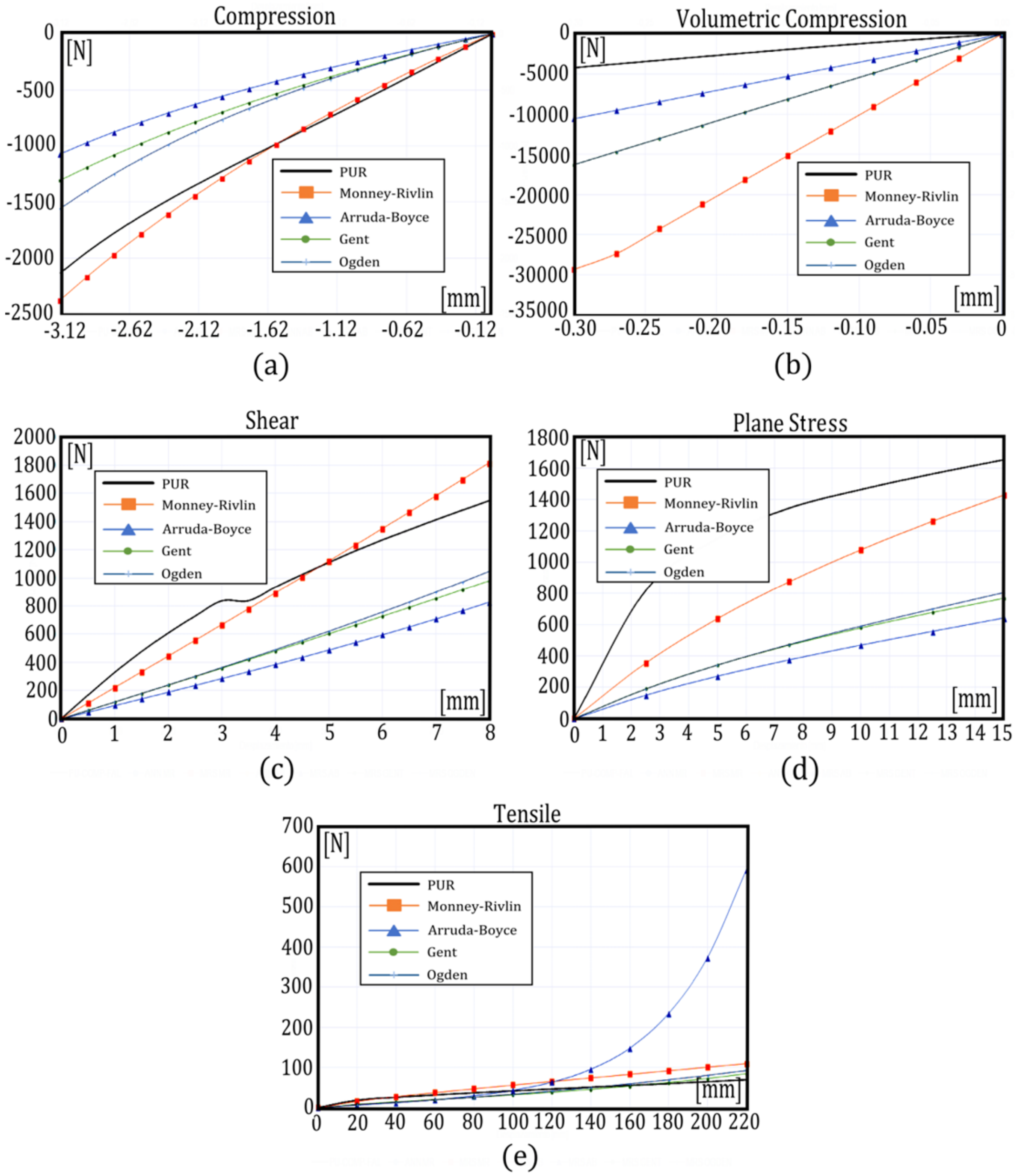

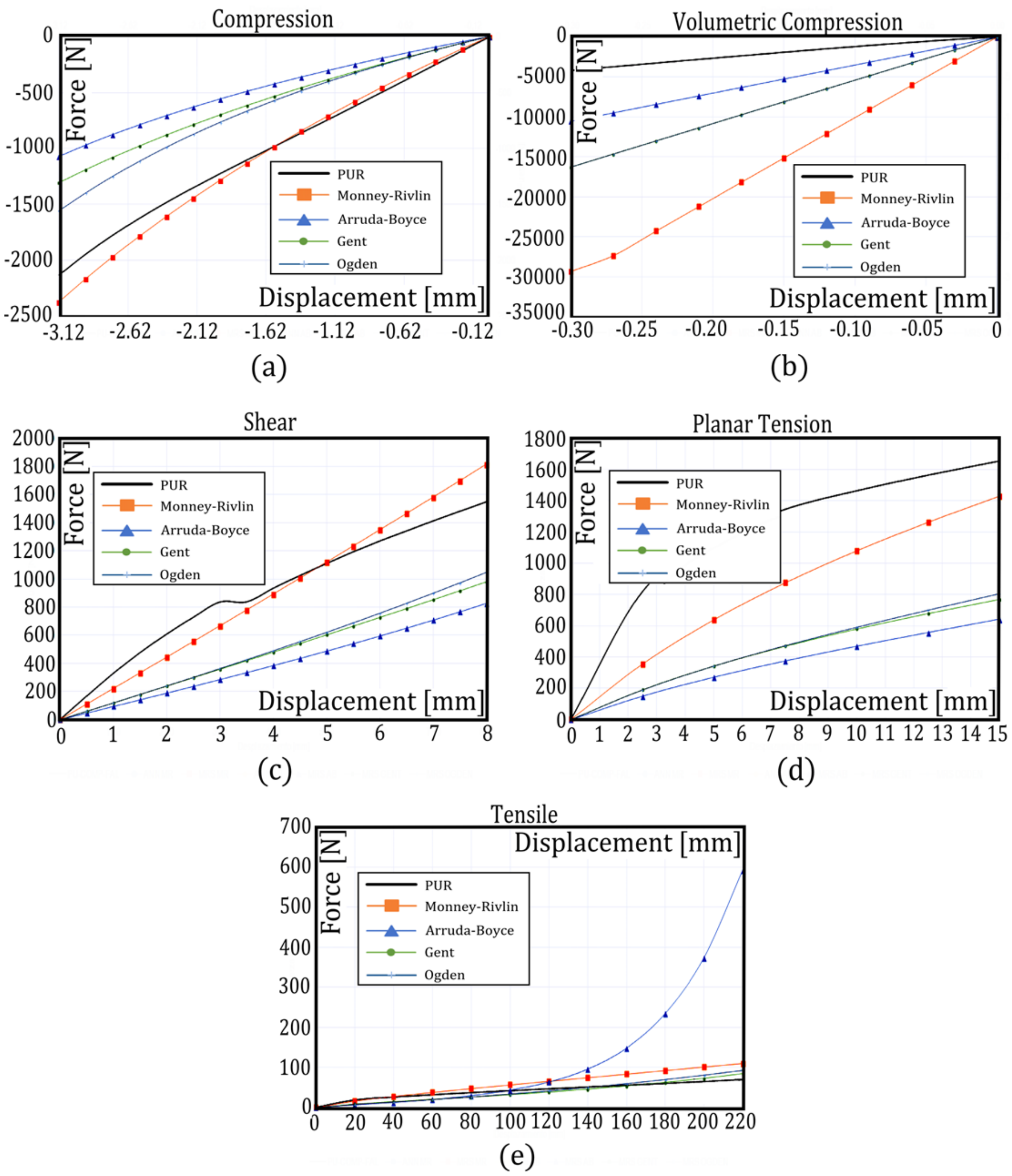

Figure S14. Force-displacement curve obtained from the FE simulations when the optimal constants Ci are compared to the force-displacement obtained experimentally for the Hyper-elastic Mooney-Rivlin model for PUR material in standardized tests: (a) Compression, (b) Volumetric Compression, (c) Shear, (d) Plane Stress and (e) Tensile.

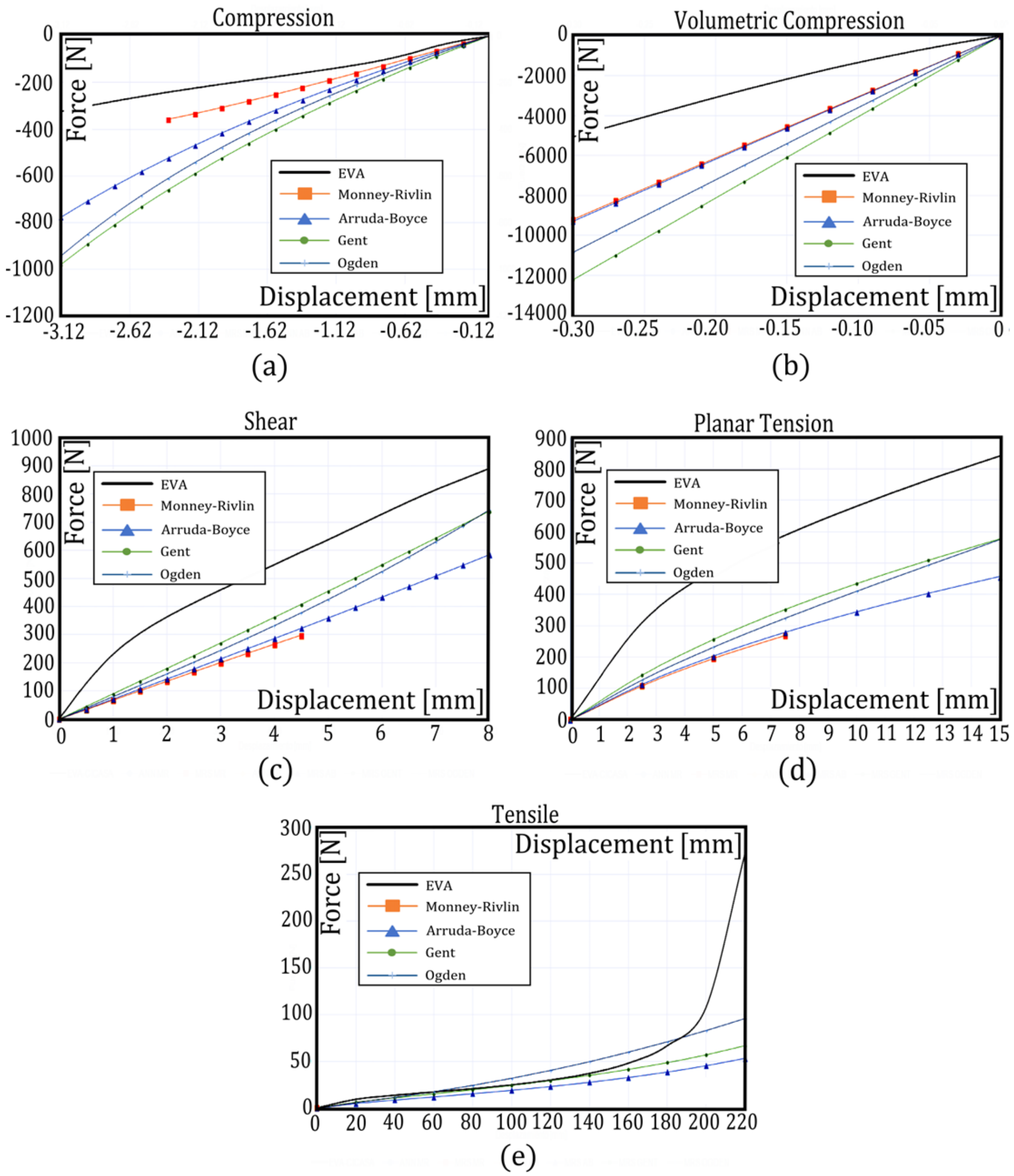

Figure S15. Force-displacement curve obtained from the FE simulations when the optimal constants Ci are compared to the force-displacement obtained experimentally for the Hyper-elastic Mooney-Rivlin model for EVA material in standardized tests: (a) Compression, (b) Volumetric Compression, (c) Shear, (d) Plane Stress and (e) Tensile.

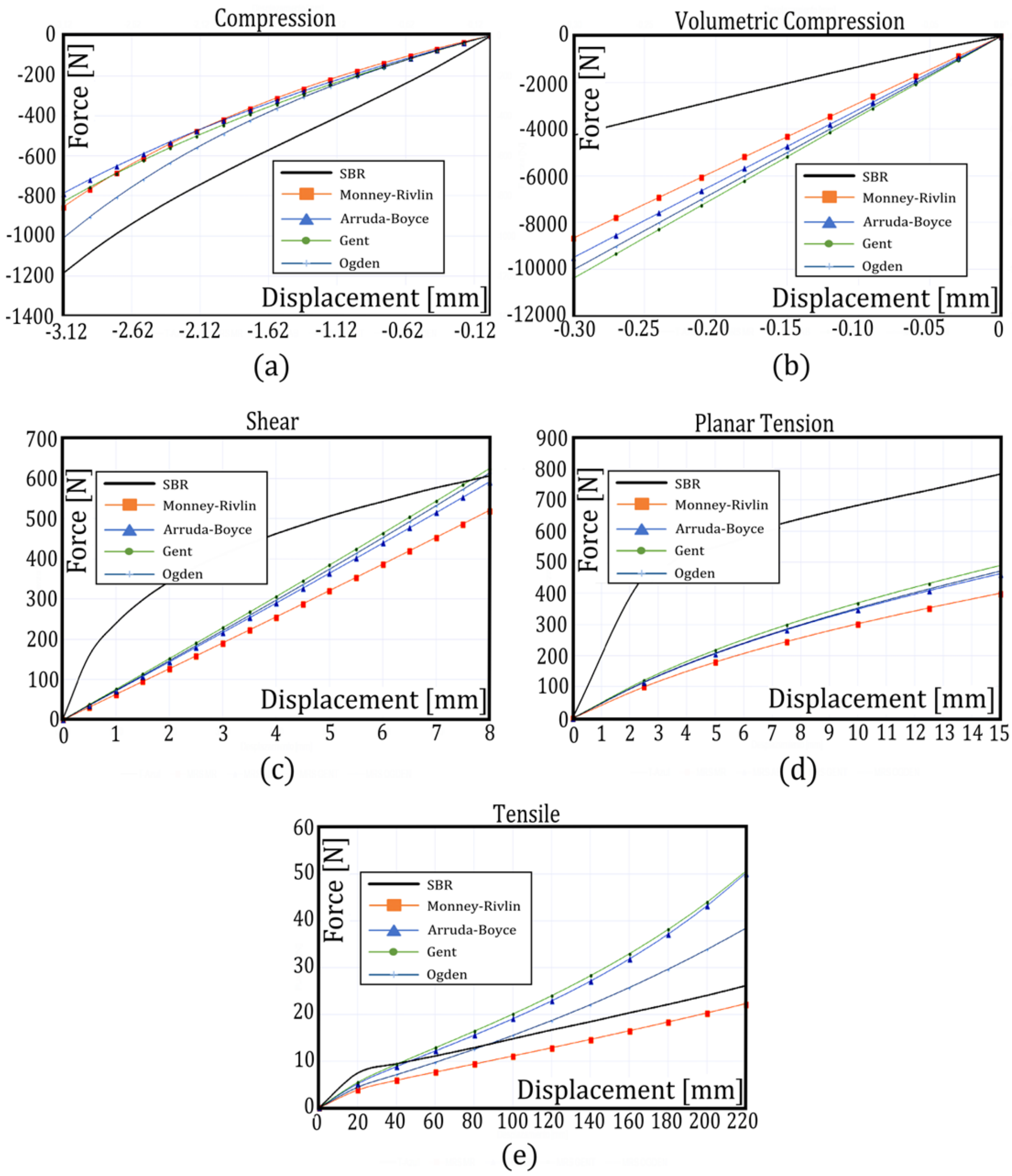

Figure S16. Force-displacement curve obtained from the FE simulations when the optimal constants Ci are compared to Vs the force-displacement obtained experimentally for Hyper-elastic Mooney-Rivlin model for SBR material in standardized tests: (a) Compression, (b) Volumetric Compression, (c) Shear, (d) Plane Stress and (e) Tensile.

© 2019 by the authors. Submitted for possible open access publication under the terms and conditions of the Creative Commons Attribution (CC BY) license (http://creativecommons.org/licenses/by/4.0/).
